# Whole-genome sequencing of bladder cancers reveals somatic *CDKN1A* mutations and clinicopathological associations with mutation burden

**DOI:** 10.1038/ncomms4756

**Published:** 2014-04-29

**Authors:** J.-B. Cazier, S.R. Rao, C.M. McLean, A.L. Walker, B.J. Wright, E.E.M. Jaeger, C. Kartsonaki, L. Marsden, C. Yau, C. Camps, P. Kaisaki, The Oxford-Illumina WGS500 Consortium, Christopher Allan, Moustafa Attar, John Bell, David Bentley, John Broxholme, David Buck, Jean-Baptiste Cazier, Richard Copley, Richard Cornall, Peter Donnelly, Simon Fiddy, Angie Green, Lorna Gregory, Russell Grocock, Edouard Hatton, Chris Holmes, Linda Hughes, Peter Humburg, Sean Humphray, Alexander Kanapin, Zoya Kingsbury, Julian Knight, Sarah Lamble, Stefano Lise, Lorne Lonie, Gerton Lunter, Hilary Martin, Lisa Murray, Davis McCarthy, Gil McVean, Alistair Pagnamenta, Paolo Piazza, Guadelupe Polanco, Peter Ratcliffe, Andy Rimmer, Natasha Sahgal, Jenny Taylor, Ian Tomlinson, Amy Trebes, Andrew Wilkie, Ben Wright, Chris Yau, J. Taylor, J.W. Catto, I.P.M. Tomlinson, A.E. Kiltie, F.C. Hamdy

**Affiliations:** 1 Bioinformatics Group, University of Oxford, Old Road Campus Research Building, Oxford OX3 7DQ, UK; 2 Cancer Research UK, Oxford Cancer Research Centre, University of Oxford, Old Road Campus Research Building, Oxford OX3 7DQ, UK; 3 Botnar Research Centre, University of Oxford, Oxford OX3 7LD, UK; 4 Nuffield Department of Surgical Sciences, University of Oxford, Old Road Campus Research Building, Oxford OX3 7DQ, UK; 5 Gray Institute for Radiobiology and Oncology, Department of Oncology, University of Oxford, Old Road Campus Research Building, Oxford OX3 7DQ, UK; 6 Bioinformatics and Statistical Genetics Core, Wellcome Trust Centre for Human Genetics, Oxford OX3 7BN, UK; 7 Molecular and Population Genetics Laboratory, Wellcome Trust Centre for Human Genetics, Oxford OX3 7BN, UK; 8 Yau Group, Wellcome Trust Centre for Human Genetics, Oxford OX3 7BN, UK; 9 NIHR Comprehensive Biomedical Research Centre, Wellcome Trust Centre for Human Genetics, Oxford OX3 7BN, UK; 10 Academic Urology Unit, University of Sheffield, Sheffield S10 2JF, UK; 11 These authors contributed equally to this work; 12 The Wellcome Trust Centre for Human Genetics, Roosevelt Drive, Oxford OX3 7BN, UK; 13 Office of the Regius Professor of Medicine, Richard Doll Building, Roosevelt Drive, Oxford OX3 7LF, UK; 14 Illumina Cambridge Ltd., Chesterford Research Park, Little Chesterford, Essex CB10 1XL, UK; 15 NIHR Oxford Biomedical Research Centre, Oxford OX3 9DU, UK.; 16 Weatherall Institute of Molecular Medicine, University of Oxford; John Radcliffe Hospital Headington, Oxford OX3 9DS, UK; 17 Imperial College London, South Kensington Campus, London SW7 2AZ, UK

## Abstract

Bladder cancers are a leading cause of death from malignancy. Molecular markers might predict disease progression and behaviour more accurately than the available prognostic factors. Here we use whole-genome sequencing to identify somatic mutations and chromosomal changes in 14 bladder cancers of different grades and stages. As well as detecting the known bladder cancer driver mutations, we report the identification of recurrent protein-inactivating mutations in *CDKN1A* and *FAT1.* The former are not mutually exclusive with *TP53* mutations or *MDM2* amplification, showing that CDKN1A dysfunction is not simply an alternative mechanism for p53 pathway inactivation. We find strong positive associations between higher tumour stage/grade and greater clonal diversity, the number of somatic mutations and the burden of copy number changes. In principle, the identification of sub-clones with greater diversity and/or mutation burden within early-stage or low-grade tumours could identify lesions with a high risk of invasive progression.

## Abstract (web summary)

Bladder cancer is a complex genetic disease and a common cause of death due to malignancy. Here, the authors carry out whole-genome sequencing of 14 bladder cancers to characterize the genomic landscape of the disease and show that mutational burden is associated with tumour progression in these samples.

## Graphical abstract

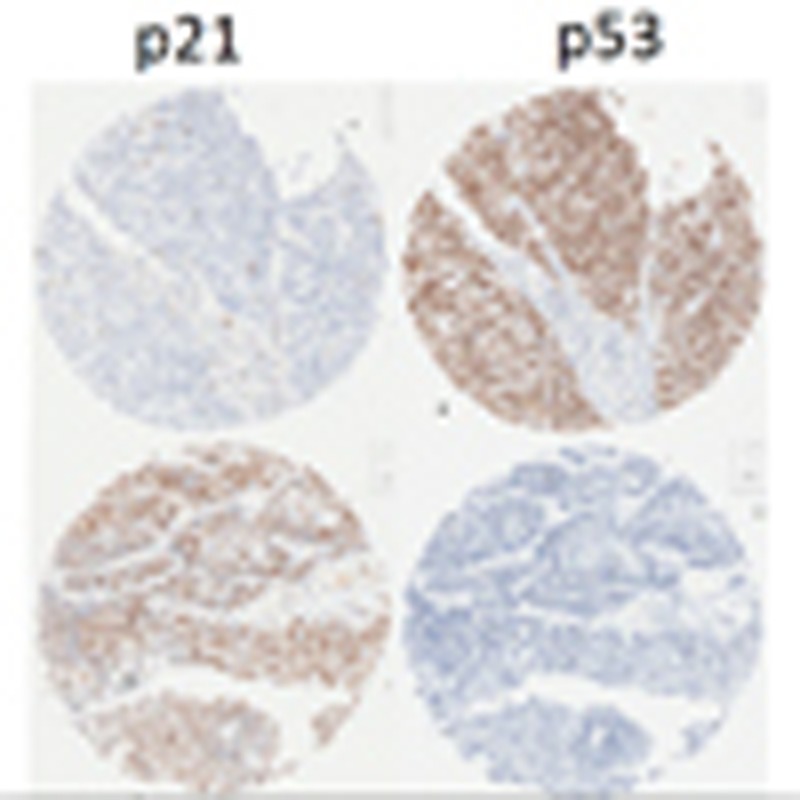

Bladder cancer is the seventh most common cancer in the developed world, with tobacco smoking a major cause ( http://www.wcrf.org/cancer_statistics/developed_countries_cancer_statistics.php). Management of non-muscle-invasive and/or low-risk disease (pTa, papillary, or flat carcinoma *in situ*) focuses on prevention of progression and identification of patients likely to develop high-risk tumours (pT1G3, high grade, invading lamina propria) in order to offer them early, aggressive intervention, including radical cystectomy^1^. However, there is a likelihood of overtreating some pTa or pT1 tumours that would never have progressed to the more life-threatening muscle-invasive disease (pT2+, invading muscularis propria). Two different genetic T1G3 sub-types have been reported, one derived from pTa disease with *FGFR3* and *HRAS* overexpression and/or mutations, and the other from flat carcinoma *in situ* with *TP53*, *RB1* and *PTEN* mutations and/or loss^2^. Whole-exome sequencing of bladder tumours has recently identified mutations of chromatin-remodelling genes, such as *KDM6A* and *MLL3* (ref. 3), and of *STAG2,* which encodes a protein involved in sister chromatid cohesion^4^,^5^,^6^. Moreover, four mutation signatures, principally comprising different combinations of C:G>T:A and G:C>C:G substitutions, have been detected in some bladder cancers, although their origins remain unclear^7^. Whole-genome sequence data at sufficiently high depth for comprehensive variant calling have not previously been reported for bladder cancers.

Here we perform whole-genome sequencing of bladder cancers of various stages and grades to search for driver mutations, chromosome-scale somatic changes, mutation signatures and clonal structures. We report the identification of mutations in the p53 effector *CDKN1A* (p21^WAF1/CIP1^) and the protocadherin *FAT1,* describe the genomic landscape of the set of cancers, and detect associations between tumour stage and measures of mutation burden.

## Results

### Overview of the bladder cancer genome

Our discovery set for whole-genome sequencing comprised 14 bladder cancers (Table 1), which were sequenced at a median depth of ~80 × , alongside paired constitutional DNA from peripheral blood. Making use of the Stampy and Platypus programs for mapping, aligning and calling variants, the number of somatic base substitution mutations (single nucleotide variants (SNVs)) called with high confidence ranged from 27,490 to 121,016 per cancer (median=88,215). Of these, a median of 219 changes (range 82–585) were potentially functional (non-synonymous, splice site, stop-gain or stop-loss) substitutions within protein-coding regions. The mean non-synonymous:synonymous mutation ratio was 1.76. Other mutation summary statistics are shown in Table 2 and Supplementary Table 1.

**Figure 1:**
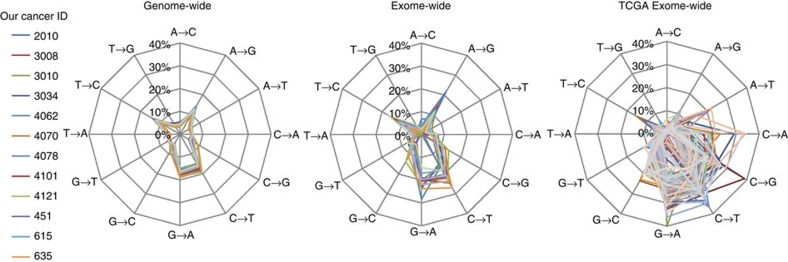
Somatic SNV spectrum genome**-**wide. The proportions of somatic SNVs of each type are shown for each of our cancers (whole genome or exome) in comparison with the TCGA bladder cancer data (exomes).

The genome-wide SNV spectrum (Fig. 1) showed no single predominant mutation type, with C:G>T:A changes being most common, followed by T:A>C:G and C:G>G:C. This mutation spectrum was broadly similar across all 14 tumours (Fig. 1; Supplementary Fig. 1) and was significantly different from all the four recently described bladder cancer mutation signatures (1b, 2, 5 and 13)^7^. However, our mutation spectrum was similar to signature 1b, related to spontaneous demethylation of methyl cytosine, across most substitutions apart from G:C>A:T and C:G>G:C. The signature profiles of the somatic mutations in our samples differed subtly at the exomic and genome-wide levels (Fig. 1). C:G>T:A changes, for example, were more common in the exome than whole genome (41 versus 33%, respectively), perhaps reflecting the greater G:C content in the former.

The number of high-confidence somatic insertion–deletion (indel) mutations of a few base pairs (bp) ranged from 5,118 to 26,407 per tumour (median=21,704). There was a strong correlation between the numbers of SNVs and indels in each cancer (details not shown). A median of 31 indels (range 6–54) per tumour were potentially functional within coding regions (frameshift, stop-gain, stop-loss or splice site).

We searched for regional SNV and indel hotspots (including ‘kataegis’) genome-wide within 1 Mb windows (Supplementary Fig. 2). Most of the hotspot regions identified lay near centromeres and telomeres, probably reflecting the increased difficulty in mapping and calling variants there, but possibly resulting from impaired access of DNA repair machinery to these heterochromatic sites. We also identified several euchromatic mutation hotspots, but these could all be accounted for by (i) large duplicated regions prone to variant miscalling (generally present in many tumours) or (ii) local copy number increases (generally present in single cancers).

We validated a subset of mutations using an Ion Torrent 150 cancer-gene panel (see Methods). Ninety-seven per cent of variants reported using the Illumina-Stampy–Playtpus pipeline were confirmed by the Ion Torrent technology.

### Structural variation

All 14 cancers with genome-wide sequence data were analysed for copy number changes using OncoSNP-Seq. In addition, a sub-set of 10 cancers was analysed using Illumina Omni1M SNP arrays, with high concordance between the methods (median 75.6%). Somatic copy number changes were present in all 14 genomes (Supplementary Fig. 3; Supplementary Data 1). The proportion of the genome showing copy number changes ranged from 12% (#4101) to 69% (#3034). Frequent large-scale copy number changes (found in six or more tumours) included gains of 1q, 3q and 8q, as well as loss of chromosome 9. Very few regions of high-level copy number gain were found, an exception being amplification around the *CCND1* oncogene on chr11q13.3 and at the *FGFR3* locus on chr4p16.3, each in a single cancer (#4121 and #615, respectively). Two cancers (#615 and #3008) had acquired homozygous deletions at the *CDKN2A* locus (chr9p21.3), and other homozygous deletions present in single cancers involved genes such as *PPARGC1A, RFX3* and *FAT1* (Supplementary Table 2). With the exception of *CDKN2A* and *FAT1* (see below), we found no good evidence from our own or The Cancer Genome Atlas (TCGA) mutation data to support the notion that any of the homozygously deleted genes acted as bladder cancer tumour suppressors.

We specifically searched for chromosome arms that had undergone chromothripsis, which we defined for convenience as evidence of at least 10 copy number and/or loss of heterozyosity (LOH) transitions on a single arm. Cancer #745, which had the most chromosomally unstable genome of the tumours analysed, showed chromothripsis involving chromosome arms 3p, 5q, 6p and X (Supplementary Fig. 4). Three other cancers had one or two arms with chromothripsis, but no recurrent region was observed across the set of 14 tumours.

Using BreakDancer followed by specific quality filtering (see Methods), we found between 618 and 3,550 predicted inter-chromosomal breakpoints in each tumour. To reduce complexity, we focussed on anchor points lying within gene boundaries, resulting in a range of 18–86 inter-chromosomal changes per cancer (Supplementary Data 2), although no anchor points lay within an exon.

### Driver genes

**Figure 2:**
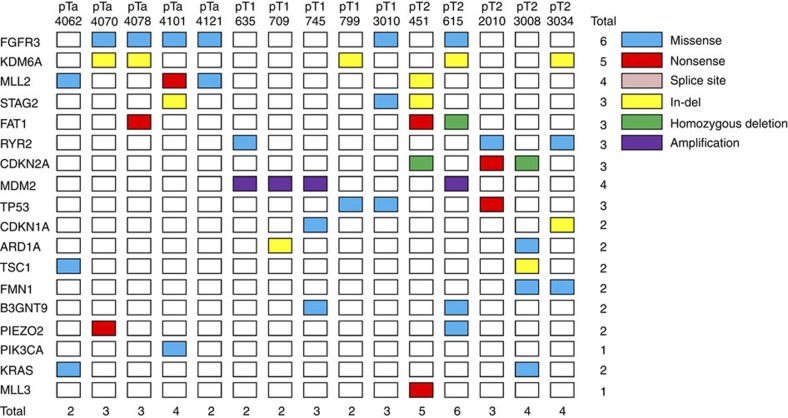
Summary of known and putative somatic-driver mutations after filtering in each cancer. *MDM2* amplification is included in the figure for comparison with *TP53* and *CDKN1A.* The established driver mutations *PIK3CA* and *MLL3* are included despite being present in only one tumour to allow comparison with the presence of other mutations. Note that only mutations passing our filtering criteria are shown here, and that other potentially pathogenic mutations of lesser predicted functional effects may exist in these cancers.

For discovery of new bladder cancer-driver mutations, we restricted our analysis to protein-coding regions. In addition to our discovery set cancers, exome sequence data from one additional stage pTa bladder cancer were available, making a total of 15 bladder cancers analysed for driver gene detection. We filtered somatic mutations to exclude all base substitutions with moderate or benign predicted functional effects (Sorting Intolerant From Tolerant (SIFT) score >0.2 and Polyphen2 score <0.8). All protein-truncating and splice-site mutations were retained. We then removed from this list genes that were mutated in only one cancer. We inspected all mutant sequencing reads manually and excluded any of evidently poor local quality. Nineteen genes survived this process (Table 3; Fig. 2), including several—for example, *TP53*, *FGFR3*, *ARID1A* and *KDM6A*—which at the time of analysis had previously been identified as bladder cancer-related genes^4^,^5^,^8^,^9^.

Prioritized genes that had not previously been reported as bladder cancer drivers included *STAG2, B3GNT9, FAT1* and *CDKN1A.* We chose *STAG2, MLL2/KMT2D* and *CDKN1A* for further replication testing, based on the presence of at least one protein-truncating mutation and the paucity of mutations previously reported in cancers. Furthermore, all three genes were excellent functional candidates. STAG2 protein is required for the cohesion of sister chromatids after DNA replication and interacts directly with RAD21 in the cohesin complex. MLL2 is a histone methyltransferase. CDKN1A (p21^WAF1/CIP1^) is a regulator of progression in G_1_ and S phases of the cell cycle^10^, and is controlled by p53 in response to a variety of stress stimuli. p21 expression changes have been found in multiple tumour types, but mutations have very rarely been reported. We validated the *STAG2* and *CDKN1A* changes detected by whole-genome sequencing using Sanger sequencing in the discovery set of tumours, and then undertook replication testing in a set of 35 additional bladder cancers (Supplementary Table 3). Four further protein-truncating and missense *CDKN1A* mutations were found (Supplementary Table 4), and of the total of six *CDKN1A-*mutant cancers in our set, two (33%) showed LOH. One of the 35 replication cancers had a somatic mutation in *STAG2.* This was a splice-site change that we showed to affect mRNA splicing (Supplementary Fig. 5). Subsequent to us performing this work, *STAG2-* and *MLL2/KMT2D*-driver mutations were reported in bladder cancers by other groups^4^,^5^,^6^, and we did not proceed to replication test *MLL2/KMT2D* in our own samples.

Two of our 15 sequenced cancers had acquired nonsense mutations in *FAT1* (Table 3), which encodes a large protocadherin that contains multiple transmembrane repeats. One of these two cancers showed LOH at *FAT1*. In addition, we detected a third cancer with predicted homozygous deletion of *FAT1* (Supplementary Table 2). Although testing of *FAT1* was impractical in our replication samples owing to moderate DNA quantity and the large size of the *FAT1* gene, we performed *in silico* replication using publicly available data from 99 bladder cancers in the TCGA consortium. That group’s data showed two tumours with protein-truncating *FAT1* mutations and a further cancer with a missense change, p.Ile2276Met, predicted to have severe functional effects (SIFT=0.05, Polyphen2=0.87).

We then examined all the bladder cancer-driver genes for mutations outside protein-coding regions. Two SNV mutations in untranslated regions were found, one in the 3′UTR of *ARID1A* and the other in the 3′UTR of *FGFR3*, but neither had predicted effects on miRNA binding or mRNA structure (details not shown). No promoter mutations were detected. We then examined 50 kb regions around the driver genes for somatic mutations that might disrupt regulatory elements. On the basis of the reasoning that the most functionally profound changes were likely to involve severe disruption of regulatory regions or motifs, we focused on indels of ≥5 bp. A single change fulfilling these criteria was found, a 13-bp deletion upstream of *FGFR3* (chr4:1,754,064; 5′-TCCCCCGCCCCAAC-3′>T) that removed a site shown to be methylated in multiple tissue types ( http://genome.ucsc.edu/).

Pathway analysis (Supplementary Fig. 6) reassuringly found ‘bladder cancer’ to be first in the list of mutated gene sets. Less expected were the findings that mutations in Wnt signalling, adherens junction and Notch signalling pathways were over-represented, given that none of the top driver mutations played principal roles in any of these processes.

### The *MDM2* amplicon

**Figure 3:**
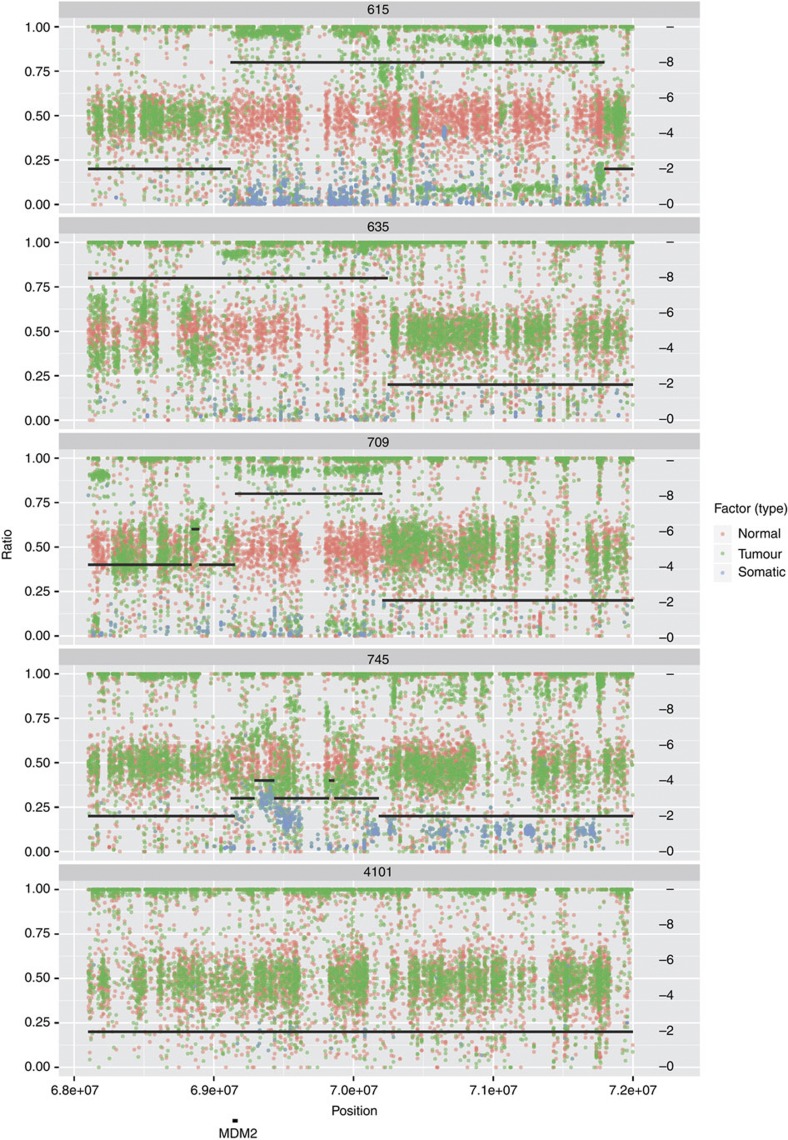
The *MDM2* amplicon. SNV allele frequencies (left-hand y axis) across each of the amplicons in the four cancers (#615, #635, #709 and #745) with copy number gain around *MDM2* are shown. The red dots correspond to variants present in the constitutional DNA, green to the same variants in the tumours and blue to somatic SNVs. Estimated copy number in each region (right-hand y axis) is shown as a black line for guidance, although close inspection of the allelic frequency changes suggests that the level of amplicon complexity is not fully captured. A tumour without *MDM2* region amplification (#4101) is shown for comparison. The position of *MDM2* (chr12:69,201,971–69,239,320) is shown below the plots.

Our copy number assessment had identified a small, recurrent duplication/amplification on chromosome 12 (minimal region 69.1–70.0 Mb) that involved the *MDM2* gene, a known driver oncogene in bladder cancer^11^ and presumed target of the copy number gain (Figs 2 and 3). None of our cancers, whether with or without copy number gain at this site, and no TCGA cancers harboured additional activating mutations in *MDM2*. In addition to *MDM2,* genes contained within the amplicons included *NUP107, CPM, CPSF6, LYZ, YEATS4* and *FRS2.* The chr12 duplication was present only in invasive lesions and, as expected, was mutually exclusive with *TP53* mutations. The availability of whole-genome sequence allowed us to investigate the amplicon structure in detail, using copy number predictions and allele frequency data for both germline polymorphisms and somatic mutations (Fig. 3). The amplicons in all four cancers had a proximal boundary close to chr12:69.1 Mb, although only two cancers appeared to share an identical breakpoint at this site. The distal amplicon boundary was more variable, ranging from chr12:70.2 to chr12:71.9 Mb (Fig. 3). We noted that two regions of homology, to chromosomes 5q and 8q, lay close to the breakpoint sites, but we found no evidence of translocations between the *MDM2* region and these sites. It appeared that all the amplicons resulted from local structural rearrangements.

In none of the four cancers was the region of amplification structurally simple. Estimated copy number at *MDM2* varied from 3 to 8. However, total copy number and allelic ratios varied along each amplicon, with intercalated regions of high- and low-level amplification. *MDM2* was located in a region of high-level copy number (CN=8) in three of the four tumours (#615, #635 and #709), and within a region of single copy gain in the remaining tumour (#745). Allele-specific assessment within the amplicons (Fig. 3) showed severe allelic asymmetry in the three cancers with high-level gain, suggesting that a single copy of chromosome 12 had become destabilized in the region. Somatic SNV and, particularly, indel frequencies were grossly elevated above background and had independently been identified as regions of possible kataegis (Supplementary Fig. 2). The elevation in indel mutation numbers could not solely be accounted for by the increased total copy number, suggesting that it might be linked to the region’s instability in some way, although errors in mapping of chimaeric reads could not be excluded. The SNV mutation spectrum within the amplicon did not differ significantly from the genome-wide spectrum of each cancer (Fig. 1). Overall, these data strongly suggested that a single copy of chromosome 12 had acquired a fundamentally destabilized region around *MDM2* in at least three, and possibly four, tumours.

### TP53 and CDKN1A protein expression in bladder cancers

Immunohistochemistry (IHC) was performed on 46 additional bladder cancer patients for both p53 and p21 proteins. Using cutoff points of 5, 10 or 20% of tumour cells displaying nuclear immunoreactivities for both proteins, we observed no significant correlation between *TP53* and *CDKN1A* expression (Supplementary Table 5; Supplementary Fig. 7). The results remained similar with full semi-quantitative scores.

### Evolution and clonal structure

**Figure 4:**
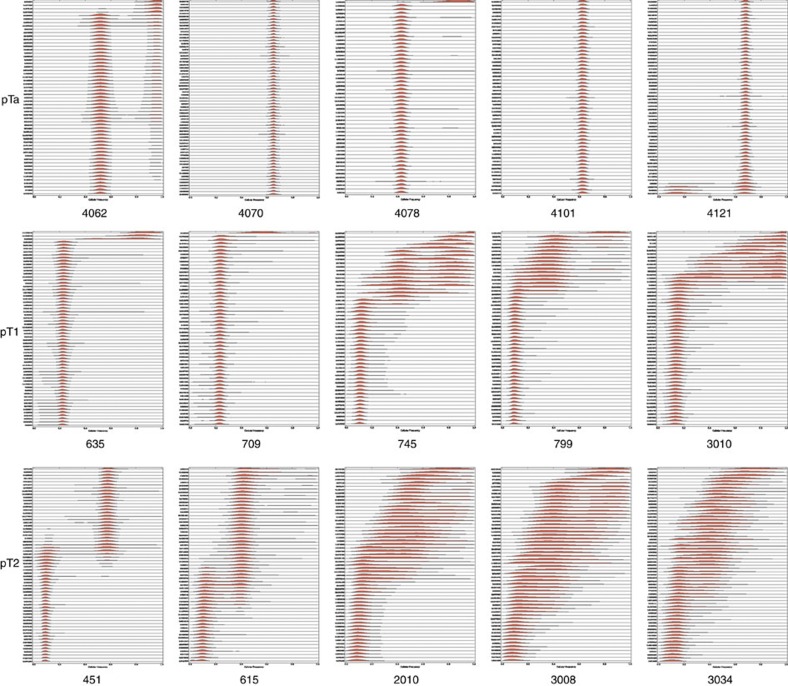
Clonality across the tumours. Summary of the frequency distributions of the key somatic mutations plus 50 random SNVs (*y* axis) for each tumour. The *x* axis shows the frequency of each mutant between 0 and 1. See Supplementary Data 3 for more details.

Clonal structure was evaluated using Pyclone, incorporating the bladder cancer-driver mutations and 50 random SNVs (Fig. 4; Supplementary Data 3). Two pTa tumours (#4070 and #4101) showed only a single clonal cluster and three cancers (#4078, #709 and #4121) appeared monoclonal apart from one outlier. The other cancers showed good evidence of multiple clones, with a maximum number of 36 clonal clusters in tumour 3008. Tumour 451 was notable for its predicted early divergence into two clones, each distinct from the other but with high levels of similarity within each sub-clone. We tested the hypothesis that cancers with greater genetic diversity (larger numbers of sub-clones) would also have higher SNV/indel burden and/or a greater proportion of the genome showing copy number aberrations, and found that both were the case (Spearman’s *ρ*=0.83, *P*=0.0002 and *ρ*=0.61, *P*=0.021, respectively).

### Clinicopathological-molecular associations

The number of cancers analysed by whole-genome sequencing was too low to reliably analyse associations between clinicopathological variables and specific mutations, although we noted that *FGFR3* mutations were more common in pTa tumours, as expected. All invasive (pT1 and pT2) lesions had *TP53, CDKN1A* or *CDKN2A* mutations or *MDM2* amplification, and none of these genes had changes in pTa tumours. We also tested for differences in mutation burden among the non-invasive, early invasive and muscle-invasive tumour groups. Since no measure of clonal structure or mutation burden differed significantly between pT1 and pT2 cancers (*P*>0.15, *N*=10, Wilcoxon test), we combined these groups for further analysis. We found that the number of somatic mutations, the proportion of the genome affected by copy number changes (a surrogate for chromosomal instability) and the number of predicted sub-clones were all significantly positively associated with increasing stage (*P*=0.0047, *P*=0.0046 and *P*=0.060, respectively; Wilcoxon test, pTa versus pT1/pT2). Grade (G1,2 versus G3), which was positively correlated with stage, was similarly associated with the same three molecular burden variables (*P*=0.0041, *P*=0.0062 and *P*=0.0082). Age, sex and smoking status (ever versus never) were not associated with any of the three measures of mutation burden and diversity.

Mutation spectra (proportions of different types of SNVs) were not in general associated with clinicopathological variables, including age and smoking history. One possible exception was the proportion of T:A>G>C changes, which was positively associated with higher stage (*P*=0.037, Kruskal–Wallis test).

## Discussion

Our whole-genome sequencing of bladder carcinomas of various sub-types has confirmed the known bladder cancer-driver genes, including our independent discovery of *STAG2-*driver mutations that have recently been reported by other groups^4^,^5^,^6^ (Table 3; Fig. 2). We additionally identified and replicated mutations in *CDKN1A* that are strongly predicted to impair protein function, in both our discovery and replication data sets. In some cases, these *CDKN1A* changes were accompanied by loss of heterozygosity. Our findings were further supported by inspection of TCGA exome sequencing data from 99 bladder cancers, which at the time of analysis in October 2013 showed two somatic SNVs in *CDKN1A,* one predicted to have strongly deleterious effects (p.Gly61Val, SIFT 0.00, Polyphen2 0.997) and the other to have possibly damaging effects (p.Asp136His, SIFT=0.11, Polphen2=0.553). Some years ago, Lacombe *et al.*^12^ reported a single bladder cancer, out of 27 analysed, with a protein-truncating *CDKN1A* mutation, and we additionally note that contemporaneous TCGA bladder cancer exome sequencing data (January 2014) show 18 *CDKN1A-*mutant bladder cancers of a total of 130 analysed^13^. We conclude that *CDKN1A* is a tumour suppressor gene in bladder carcinoma, and we note that its inactivation is a bladder-specific change, since >1% of cancers of other sites harbour *CDKN1A* mutations ( http://cancer.sanger.ac.uk/cosmic/gene/overview?ln=Cdkn1a).

*CDKN1A* and *CDKN2A* mutations were mutually exclusive in both our and TCGA data. Although p21, the protein encoded by *CDKN1A,* acts downstream of p53, we found one cancer (#1992) from our full set of 50 tumours to have both *CDKN1A* (p.Tyr77del) and *TP53* (p.Arg213Pro) mutations (details not shown) and another (#745) to have *CDKN1A* mutation (p.Trp65Arg) and low-level *MDM2* amplification (Supplementary Table 4). Interestingly, p21 protein has variably been described as having oncogenic and anti-oncogenic effects in a number of cancer types^14^. The genetic data strongly suggest that loss of p21 function promotes the growth of bladder carcinomas. Our genetic data also strongly suggest that loss of p21 function may augment defects caused by inactivation of p53, but does not act as a simple substitute. Our IHC data support this contention. Cancers with low or absent p53 might have various underlying causes (including not only protein-destabilizing *TP53* mutations, *MDM2* amplification, chr9p21 deletions involving p14^ARF^, but also a normal p53 pathway. However, stabilization of p53 protein is generally accompanied by pathogenic missense *TP53* mutations, and these cancers would not generally be expected to activate p21 expression^15^. The finding by ourselves and others^16^ that a proportion of bladder cancers expresses both p53 and p21 suggests that p21 can be activated by a p53-independent pathway in these tumours, providing a rationale for some of them acquiring both p21 and p53 mutation.

In addition to identifying *CDKN1A* mutations, we have highlighted inactivating *FAT1* mutations as possible bladder cancer drivers, on the basis of protein-truncating mutations and deletions in our own and TCGA data. *FAT1* is a tumour suppressor in *Drosophila* and an excellent candidate for having a similar role in humans. It functions in cell adhesion and is expressed in the urinary tract. However, *FAT1* is an extremely large gene, and hence it is not yet possible to assign driver or passenger status to the mutations we have found. We additionally identified recurrent mutations in *RYR2, FMN1, B3GNT9* and *PIEZO2* in our discovery set of cancers, but the importance of these genes for bladder carcinogenesis remains uncertain.

The mutation spectra of our bladder cancers did not closely match any of the 22 specific signatures previously reported, although there were similarities to signature 1b (ref. 7). Specifically, we did not find evidence of a carcinogen signature or a preponderance of G:C>C:G mutations reported in some bladder cancers^17^. The differences between the two data sets might in part result from the smaller size of our cohort, or from differences in the types of tumour sequenced, but we suggest that the G:C>C:G mutation signature is not common in bladder cancer.

The use of genome-wide deep sequencing allowed us to perform extensive work on structural variations, including copy number changes and translocations. Although no recurrent translocations with intragenic breakpoints were found, several focal regions of deletion and gain were detected. Specifically, we characterized the structure of the recurrent amplification around *MDM2*, which was found in 4 of 10 invasive tumours. This analysis showed that in most of these tumours, there was heterogeneity in copy number across the amplicon. Although there was no evidence of activating *MDM2* mutations, amplification in most cancers was greatly asymmetric with respect to constitutional polymorphisms, suggesting that one copy of chromosome 12 became locally destabilized, leading to the observed rearrangements and, perhaps, an elevated frequency of small indels. Breakdancer analysis did not indicate the translocation of the *MDM2* amplicon to any other chromosome, although we cannot exclude its location on double-minute chromosomes.

There were clear associations between tumour stage and molecular features. In general, invasive tumours had larger burdens of mutations of all types, including SNVs and copy number changes. The invasive tumours also showed greater clonal diversity. With the possible exception of T:A>G>C changes, there was no clear association between stage and SNV mutation spectrum, suggesting that invasion was not driven by mutagen exposure or specific defects in DNA repair (although defects causing chromosomal instability remained a possible cause of invasive behaviour). It will be of interest for larger future studies to assess associations between molecular phenotypes, such as mutation burden, and other measures of bladder cancer behaviour, including prognosis. Furthermore, it will be important to test the scenario that some early-stage bladder cancers harbour sub-clones with p53 or p21 inactivation, and/or greater diversity and/or mutation burden, thus allowing the identification of tumours with a high risk of progression to invasive disease and permitting more appropriate and intensive management.

## Methods

### Sample description

Ethics approval was obtained from South Yorkshire Research Ethics Committee (project 10/H1310/73), Oxfordshire Research Ethics Committee C (project 09/H0606/5), Leeds (East) Research Ethics Committee (project 04/Q1206/62) and NRES Committee London-Bromley (project 13/LO/0540).

The discovery set of tumour cancers with whole-genome sequence data comprised 14 bladder cancers, paired with peripheral blood, that had been collected from unrelated individuals presenting to the Urology Department, Royal Hallamshire Hospital, Sheffield, between June 2008 and September 2011. Four cancers were of low-grade papillary morphology (pTaG1-2: #4062, #4070, #4101 and #4121), five were high grade invading the lamina propria (pT1G3: #635, #709, #745, #799 and #3010, with #635 and #3010 subsequently becoming muscle invasive) and five were muscle invasive (pT2-pT3: #451, #615, #2010, #3008 and #3034). Exome sequence data were available from a further low-grade cancer, #4078. All tumours were sampled at transurethral resection or cystectomy and had not previously received any other therapy. The presence of at least 70% cancer cells in the tumour specimens was confirmed by routine histological assessment. Genomic DNA was extracted from each tumour and paired blood sample using standard methods.

The replication set comprised 35 frozen biopsies of bladder carcinomas (16 pTa or pTis, 10 pT1G3, 9 pT2) from patients who had donated samples to the Oxford Radcliffe Biobank. Each sample was microdissected to maximize the amount of tumour, using haematoxylin and eosin-stained slides as a guide. After confirming that each specimen included a majority of cancer cells, DNA was extracted using standard methods of digestion with Laird’s lysis buffer plus proteinase K, followed by phenol–chloroform extraction and ethanol precipitation. For selected cancers where mutations were detected, the somatic origin of those changes was confirmed by microscope-guided dissection of normal tissue from the tumour specimen.

The IHC set comprised samples from 46 patients with muscle-invasive bladder cancer, treated with radical radiotherapy at Leeds Teaching Hospitals NHS Trust from January 2006 to February 2009 as described in and one further patient. The median patient age was 76.5 years (range 57.8–86.5); 33 were male (72%) and 13 female (28%). Suitable invasive tumour areas were identified by a consultant histopathologist (SB) on haematoxylin and eosin sections from formalin-fixed paraffin-embedded bladder chips obtained at pre-radiotherapy transurethral resection. Up to five 0.6 mm tissue microarray (TMA) cores per patient were embedded into a single recipient block, from which 0.4 μM sections were taken for IHC staining.

### Whole-genome sequencing

Sequencing in the discovery set was performed as part of the WGS500 project using a dedicated pipeline. Samples were quantified using the Qubit system (Invitrogen) and sequencing libraries constructed from 1 μg DNA using the NEBNext DNA Sample Prep Master Mix Set 1 Kit. Ligation of adaptors was performed using 6 μl of the Illumina Multiplexing Sample Preparation Oliogonucleotide Kit. Libraries were size selected using 2% gel electrophoresis and the distribution of fragments in the purified fraction was determined using the Tapestation 1DK system (Agilent/Lab901). Each library was PCR enriched using the following custom primers:

Multiplex PCR primer 1.0: 5′-AATGATACGGCGACCACCGAGATCTACACTCTTTCCCTACACGACGCTCTTCCGATCT-3′; index primer: 5′-CAAGCAGAAGACGGCATACGAGAT[INDEX]CAGTGACTGGAGTTCAGACGTGTGCTCTTCCGATCT-3′.

Indexes were 8 bp long and part of an indexing system developed in-house. Four independent PCR reactions per sample were prepared using 25% volume of the pre-PCR library each. After eight cycles of PCR (cycling conditions as per Illumina recommendations), the four reactions were pooled and purified with AmpureXp beads. The final size distribution was determined using the Tapestation 1DK system (Agilent/Lab901). The concentration of each library was determined by the Agilent qPCR Library Quantification kit. Samples were sequenced using the Illumina HiSeq2000 platform as paired 100 bp reads with Chemistry version 3.0, with the aim of a target average coverage of 30 × for the blood DNA and 60 × for the tumours.

After removal of PCR duplicates using Picard, reads were mapped with Stampy version 1.0.12 (r975)^18^ onto the Human Reference Genome (GRCh37d5/hg19). SNVs and small insertion–deletions (indels) were called with Platypus version 0.1.8 (ref. 19) using the tumour-normal pairs of bam files together to ensure comparable calls at every locus. Variants were only called if they were assigned a sufficiently high posterior probability (Phred score of 5). Additional filters were used to remove variants in low-quality reads. Although we removed the allele bias to increase sensitivity, we add filters on the number of high-quality reads at a location, carrying the variants and overall. Similarly, we made use of the difference between log likelihood ratios of each potential genotype to increase certainty. Finally, we made sure that the automatic call matched the data by expert visual inspection of the mapped reads onto the reference genome using read direction colouring on top of the standard Integrative Genomics Viewer (IGV) scheme ( http://www.broadinstitute.org/igv/alignmentdata/). Variants were annotated with ANNOVAR (RefSeq gene models) using: single nucleotide polymorphism database (dbSNP) (132); 1,000 genomes project allele frequencies (November 2011); University of California Santa Cruz (UCSC) segmental duplication scores; and UCSC 46 species conservation scores. Candidate variants were annotated with predictions of functional importance from SIFT and PolyPhen2.

Owing to the possibility of copy number changes and intra-tumour genetic heterogeneity, we did not apply the default strand and allele bias filters to the data. However, we selected only variants at sites with at least 10 reads. First calls were compared between matched constitutional and tumour samples to identify somatic mutations. Known variants present at frequency >0.05 in the 1,000 Genome project, dbSNP or in the Exome Sequencing Project were also removed. Variants identified in constitutional DNA from any of the other non-cancer sequencing projects of the WGS500 consortium (29 million variants across 284 samples) were discarded as being more likely due to systematic error in our pipeline than genuine somatic mutation.

### Ion Torrent sequencing

A custom 150 cancer-gene exome panel (details available on request) was used for technical validation of the whole-genome sequencing in the 15 bladder carcinomas. Approximately 100 ng DNA was used for sequencing. Mean read depth was 1,380. Variants present in the genome and exome sequence data were assessed alongside the equivalent Ion Torrent data, using both automatic calls in the Torrent Server output and visual inspection using the IGV.

### Targeted sequencing

Validation and replication were performed with bidirectional Sanger sequencing of the coding regions of *CDKN1A* and of prioritized regions (exons 8–11 and 17–25) of *STAG2* (Supplementary Table 6). In addition, well-established methods were used to screen for mutations in exons 5–8 of *TP53.* Since neither *CDKN1A* nor *STAG2* was present on the Ion Torrent cancer-gene exome panel, we also sequenced these genes in the 15 cancers with genome or exome sequence data. The somatic origin of any variation from the human reference sequence was confirmed by analysis of paired DNA from blood or normal urothelium. All PCR reaction conditions are available on request. Mutations were analysed with Mutation Surveyor V3.97 and confirmed by inspection of electropherograms.

### Splice-site mutation analysis

To assess effects on mRNA splicing of the *STAG2* mutation identified in the replication set of cancers, frozen bladder cancer was homogenized using the gentleMACS homogenizer (Miltenyi Biotec) and RNA isolated using TRIzol (Life Technologies). Bladder or pooled RNA from the First Choice Human RNA panel (Ambion) was used as a negative control. The RNA was treated with DNase I (Fermentas) to eliminate contaminating genomic DNA, and cDNA synthesis was performed using the iScript cDNA synthesis kit (Bio-Rad). Subsequently, PCR was performed on tumour or control cDNA using Phusion High Fidelity PCR master-mix (Thermo Scientific) and the products examined by minigel electrophoresis (Supplementary Table 6).

### Structural variants

For analysis of copy number, LOH and ploidy, we used the OncoSNP-Seq^7^ program, which identifies variation based on read depth and allelic fraction under various models of stromal contamination and ploidy. After careful assessment of the data, we systematically chose the model with average ploidy of the non-neoplastic part of the tumour closest to 2. Results were compiled across the samples using the online version of GREVE to highlight recurrence of changes^20^.

Breakdancer version 1.1.2 with default parameters was used to identify potential rearrangements, focusing on inter-chromosomal translocations. Results were first filtered for a score of 99, a minimum of 10 total supporting read pairs covering the breakpoints. Looking for functional rearrangement, we then reduced the list to breakpoints within genes and exons (Supplementary Data 2).

### Single nucleotide polymorphism arrays

Ten tumour:normal pairs (451, 615, 635, 709, 745, 799, 2010, 3008, 3010 and 3034) were genotyped using the Illumina Omni1 SNP array. The data were analysed using Genome Studio and OncoSNP^21^, and used to test the validity of the next-generation sequencing calls as well as the evaluation of copy number variation.

### Prediction of effects of exonic mutations outside coding regions

MicroRNA binding was assessed by the TargetScan 6.2 program for mammals ( http://www.targetscan.org/) across conserved and partly conserved miRNA families. In addition, we examined the sno/miRNA track from the Human Genome Browser ( http://genome.ucsc.edu) based on miRBase Release 15.0 (April 2010) and snoRNABase Version 3. mRNA structure was assessed using RNAfold (http http://rna.tbi.univie.ac.at/), using the default ‘minimum free energy and partition function option. In both cases, mutant and reference sequences were compared.

### Clonal structure

Allelic heterogeneity, and thus clonal structure, was evaluated using PyClone version 0.12.3.1 (ref. 22). Allelic frequencies of selected somatic mutations were obtained using the number of reads and the number of reads carrying a variant as the total copy number. The copy number value at each of these loci and the tumour content were obtained from OncoSNP-Seq model selection as described above. We performed the clonal structure assessment using the three most likely values of each cancer’s DNA index from OncoSNP-Seq—the result from the estimate with the greatest likelihood is presented herein. Other DNA index estimates produced modest quantitative differences in clonal structure (data not shown), but these were insufficient to alter qualitative conclusions regarding clonality or to affect the analysis of clonal structure and clinicopathological features of the cancers. For each sample, a specific set of genome-wide somatic mutation was selected to characterize its heterogeneity: 50 randomly selected somatic mutations across the genome were added to the candidate somatic mutations.

### Pathway analysis

Pathway analysis was performed using the online version of Intogen^23^ using the annotated, quality filtered, somatic mutations from all 15 samples with genome-wide and exome-wide sequence data.

### Immunohistochemistry

IHC analysis of p21 and p53 was carried out using a standard avidin–biotin–peroxidase technique. Briefly, TMA sections were dewaxed and rehydrated through xylene, graded ethanol and water. Endogenous peroxidase activity was blocked in 3% hydrogen peroxide for 20 min before heat-induced epitope retrieval at 110 °C for 1 min, in citrate buffer (10 mM citric acid, 0.05% Tween 20, pH 6.0) for p21 or Tris–EDTA buffer (10 mM Tris base, 1 mM EDTA solution, 0.05% Tween 20, pH 9.0) for p53 sections, and samples allowed to cool at room temperature to enhance antigen retrieval. After washing with running water and TBS, endogenous protein-binding activity was quelled using avidin–biotin blocking agent (Vector, Peterborough, UK) and 10% normal goat serum (Dako, Cambridgeshire, UK). Sections were incubated with primary antibody diluted in 1% BSA (VWR International, Leicestershire, UK) at optimized dilutions: anti-p21 (1:20, 12D1, Cell Signalling), anti-p53 (1:50,000, DO-1, Santa-Cruz), at 4 °C overnight. The Dako REAL Detection System Peroxidase/DAB+ Rabbit/Mouse kit was then employed to determine immunoreactivity, as per manufacturer’s instructions, with 1:50 (p21) and 1:200 (p53) dilutions of Dako REAL DAB+ Chromogen to Dako REAL HRP Substrate Buffer. Sections were washed, and then counterstained for 15 s in filtered Harris Haematoxylin (VWR, Leicestershire, UK), before further washing, dehydration and mounting in dibutylphthalate xylene (Leica, Peterborough, UK).

Slides were scanned using the Aperio ScanScope CS2 digital slide scanner at × 40 magnification and viewed using Aperio Image Scope viewing software. Analysis of nuclear staining was made by two independent scorers by measuring the percentage of positively stained tumour cells. A consensus was reached for intensity by taking the mean if scores were within 20% of each other; otherwise, images were reviewed and a consensus reached.

### TCGA data

TCGA somatic exomic mutation data from 99 ‘bladder urothelial carcinoma’ samples were obtained from the TCGA data portal ( https://tcga-data.nci.nih.gov/tcga/) on 31st October 2013.

## Additional information

**Accession codes:** Whole genome sequence data for 14 bladder cancers have been deposited at the European Genome-phenome Archive (EGA, http://www.ebi.ac.uk/ega/), which is hosted by the EBI, under the accession code EGAS00001000738.

**How to cite this article:** Cazier, J.-B. *et al.* Whole-genome sequencing of bladder cancers reveals somatic *CDKN1A* mutations and clinicopathological associations with mutation burden. *Nat. Commun.* 5:3756 doi:10.1038/ncomms4756 (2014).

## Supplementary Material

Supplementary Figures, Tables and References Supplementary Figures 1-7, Supplementary Tables 1-6 and Supplementary References

Supplementary Data 1 OncoSNP-Seq analysis

Supplementary Data 2 The locations of putative translocation breakpoints within exons across the discovery samples

Supplementary Data 3 Pyclone analysis

## Figures and Tables

**Table 1:** Demographic and clinicopathological features of the patients and their bladder cancers.

**Tumour no.**	**Age, years**	**Gender**	**Smoker?**	**Occupation**	**Stage**	**Grade**	**Type**	**Background urothelium**	**Cystectomy?**	**Follow-up to September 2013**
4062	69	F	Ex-	Not known	pTa	G2	Papillary	Normal	No	No recurrence
4070	74	M	Passive	Steel works	pTa	G2	Papillary	Normal	No	Recurrence, G1pTa
4078	56	M	Current	Epoxy resin worker	pTa	G2	Papillary	Normal	No	Did not attend follow-up
4101	81	M	Ex-	Sheep farmer	pTa	G1	Papillary	Normal	No	No recurrence
4121	71	F	Never	Supervisor	pTa	G2	Papillary	Normal	No	Died due to metastatic bladder cancer
635	85	M	Ex-	Plumber	pT1	G3	Papillary/solid	Cis	No	Died
709	73	F	Current	Farm worker	pT1	G3	Papillary/solid	Not sampled	No	Alive
745	83	F	Ex-	Cleaner	pT1 N0	G3	Papillary/solid	Cis	Yes	Alive
799	87	M	Ex-	Coal mining	pT1	G3	Papillary/solid	Normal	No	Died
3010	78	F	Ex-	Dry cleaner	pT1	G3	Papillary with squamous differentiation	Not sampled	No	Died
451	78	M	Ex-	Engineer	pT2	G3	Papillary/solid	Not sampled	No	Died
615	75	F	Ex-	Secretary	pT2	G2	Papillary	Normal	No	Died
2010	76	F	Ex-	Warehouse packer	pT3 N2	G3	Solid with squamous differentiation	Low-grade dysplasia	Yes	Died
3008	82	F	Ex-	Steel works	pT3b	G3	Papillary/solid with squamous differentiation	Cis	Yes	Died
3034	77	F	Never	Office worker	pT3a N0	G3	Solid	Cis	Yes	Alive

cis, carcinoma *in situ*; F, female; M, male.

Stage and grade are shown according to TNM 7th edition. Samples were removed at transurethral resection, except where cystectomy is indicated. Detailed follow-up data are shown for those presenting with pTa disease, whereas binary survival data are shown for those with invasive disease.

**Table 2:** Summary genome profiles of the cancers.

**Tumour no.**	**Type**	**Tumour %**	**ESM**	**GSM**	**CNV %**	**No. of CNVs**	**Clonality**
4062	pTa	70	146	34,966	14	654	2
4070	pTa	90	137	33,861	14	658	1
4078	pTa	80	179	—	—	—	2
4101	pTa	80	154	32,608	12	669	1
4121	pTa	70	235	40,678	19	729	2
635	pT1	70	920	113,970	42	633	2
709	pT1	70	541	110,745	39	741	2
745	pT1	80	427	122,353	67	888	20
799	pT1	80	272	123,701	47	629	13
3010	pT1	90	350	139,673	33	812	16
451	pT2	90	385	111,643	20	644	5
615	pT2	90	892	108,578	31	703	3
2010	pT2	70	482	122,547	53	710	29
3008	pT2	80	402	117,965	24	557	36
3034	pT2	80	495	99,990	69	1,233	4

clonality, number of sub-clonal clusters estimated by Pyclone; CNV %, per cent of genome affected by copy number changes; ESM, number of exomic somatic mutations; GSM, number of genomic somatic mutations; no. of somatic CNVs, number of discrete CNVs; tumour %, percentage of tumour cells in the sample.

Note that only exome sequence data were available for #4078.

**Table 3:** Known bladder cancer-driver mutations and other mutations in genes fulfilling the filtering criteria for further analysis.

**Tumour no.**	**Chr**	**Position (B37)**	**Gene**	**Details**	**LOH**
709	1	27,099,037	* ARID1A *	NM_006015:c.3453_3454insA:p.S1151fs	0
3008	1	27,106,378	* ARID1A *	NM_006015:c.A5989G:p.N1997D	1
635	1	237,765,394	* RYR2 *	NM_001035:c.G4666C:p.E1556Q	0
3034	1	237,791,238	* RYR2 *	NM_001035:c.C6298T:p.R2100W	0
2010	1	237,947,202	RYR2	NM_001035:c.G12190A:p.E4064K	0
4070	4	1,803,568	* FGFR3 *	NM_000142:c.C746G:p.S249C	0
4121	4	1,803,568	* FGFR3 *	NM_000142:c.C746G:p.S249C	0
615	4	1,803,568	* FGFR3 *	NM_000142:c.C746G:p.S249C	0
4078	4	1,806,099	* FGFR3 *	NM_000142:c.A1118G:p.Y373C	1
3010	4	1,806,099	* FGFR3 *	NM_000142:c.A1118G:p.Y373C	1
4101	4	1,806,153	* FGFR3 *	NM_000142:c.C1172A:p.A391E	0
4078	4	187,522,477	* FAT1 *	NM_005245:c.T11586A:p.Y3862X	1
451	4	187,542,642	* FAT1 *	NM_005245:c.G5098T:p.E1700X	0
3034	6	36,652,054	* CDKN1A *	NM_000389:c.176_177insG:p.L59fs	0
745	6	36,652,071	* CDKN1A *	NM_000389:c.T193A:p.W65R	1
2010	9	21,974,695	* CDKN2A *	NM_000077:c.132_133insA:p.Y44_G45delinsX	1
4062	9	135,776,983	* TSC1 *	NM_001162427:c.C2342T:p.S781F	1
4062	9	135,781,212	* TSC1 *	NM_001162427:c.C1600G:p.P534A	1
4062	9	135,781,386	* TSC1 *	NM_001162427:c.C1426G:p.Q476E	1
3008	9	135,781,446	* TSC1 *	NM_001162427:c.1365delC:p.P455fs	1
4062	12	49,427,530	* MLL2 *	NM_003482:c.G10958C:p.G3653A	0
4062	12	49,427,912	* MLL2 *	NM_003482:c.G10678A:p.D3560N	0
4121	12	49,431,499	* MLL2 *	NM_003482:c.G9640C:p.E3214Q	0
4121	12	49,431,871	* MLL2 *	NM_003482:c.G9268C:p.E3090Q	0
4121	12	49,431,937	* MLL2 *	NM_003482:c.G9202A:p.E3068K	0
4101	12	49,434,561	* MLL2 *	NM_003482:c.6991delC:p.L2331X	0
4101	12	49,438,067	* MLL2 *	NM_003482:c.C5104T:p.R1702X	0
451	12	49,439,934	* MLL2 *	NM_003482:c.4607_4608insA:p.S1536fs	0
451	12	49,445,797	* MLL2 *	NM_003482:c.1668delG:p.P556fs	0
3008	15	33,261,489	* FMN1 *	NM_001103184:c.G1744A:p.V582I	0
3034	15	33,359,823	* FMN1 *	NM_001103184:c.C263T:p.S88L	1
615	16	67,183,392	* B3GNT9 *	NM_033309:c.C997T:p.H333Y	0
615	16	67,183,539	* B3GNT9 *	NM_033309:c.C850G:p.P284A	0
745	16	67,184,036	* B3GNT9 *	NM_033309:c.T353A:p.L118Q	1
3010	17	7,577,094	* TP53 *	NM_001126115:c.C448T:p.R150W	1
2010	17	7,577,127	* TP53 *	NM_001126115:c.G415A:p.E139K	1
799	17	7,578,395	* TP53 *	NM_001126115:c.C139T:p.H47Y	0
799	17	7,578,475	* TP53 *	NM_001126115:c.C59T:p.P20L	0
615	18	10,784,798	* PIEZO2 *	NM_022068:c.G2476C:p.D826H	0
4070	18	11,066,210	* PIEZO2 *	NM_022068:c.C76T:p.R26X	—
615	X	44,820,628	* KDM6A *	NM_021140:c.326_329del:p.109_110del	—
4070	X	44,922,729	* KDM6A *	NM_021140:c.1591_1606del:p.531_536del	—
3034	X	44,941,984	* KDM6A *	NM_021140:c.3234_3235insT:p.P1078fs	—
4078	X	44,942,842	* KDM6A *	NM_021140:c.3422_3423insC:p.S1141fs	—
799	X	44,949,105	* KDM6A *	NM_021140:c.3666_3667insG:p.A1222fs	—
4101	X	123,196,821	* STAG2 *	NM_006603:c.1709delC:p.A570fs	—
451	X	123,200,065	* STAG2 *	NM_006603:c.2137_2138insA:p.Y713_K714delinsX	—
3010	X	123,202,462	* STAG2 *	NM_006603:c.T2314C:p.C772R	—

Chr, chromosome; LOH, loss of heterozyosity.

The details of the mutations are shown as: reference transcript: DNA change: protein change. For LOH: 1, present; 0, absent.

## References

[b1] Denzinger S. *et al.* Early versus deferred cystectomy for initial high-risk pT1G3 urothelial carcinoma of the bladder: do risk factors define feasibility of bladder-sparing approach? Eur. Urol. 53, 146–152 (2008). 17624657 10.1016/j.eururo.2007.06.030

[b2] Goebell P. J. & Knowles M. A. Bladder cancer or bladder cancers? Genetically distinct malignant conditions of the urothelium. Urol. Oncol. 28, 409–428 (2010). 20610279 10.1016/j.urolonc.2010.04.003

[b3] Gui Y. *et al.* Frequent mutations of chromatin remodeling genes in transitional cell carcinoma of the bladder. Nat. Genet. 43, 875–878 (2011). 21822268 10.1038/ng.907 PMC5373841

[b4] Balbas-Martinez C. *et al.* Recurrent inactivation of STAG2 in bladder cancer is not associated with aneuploidy. Nat. Genet. 45, 1464–1469 (2013). 24121791 10.1038/ng.2799 PMC3840052

[b5] Guo G. *et al.* Whole-genome and whole-exome sequencing of bladder cancer identifies frequent alterations in genes involved in sister chromatid cohesion and segregation. Nat. Genet. 45, 1459–1463 (2013). 24121792 10.1038/ng.2798 PMC7512009

[b6] Solomon D. A. *et al.* Frequent truncating mutations of STAG2 in bladder cancer. Nat. Genet. 45, 1428–1430 (2013). 24121789 10.1038/ng.2800 PMC3875130

[b7] Alexandrov L. B. *et al.* Signatures of mutational processes in human cancer. Nature 500, 415–421 (2013). 23945592 10.1038/nature12477 PMC3776390

[b8] Cappellen D. *et al.* Frequent activating mutations of FGFR3 in human bladder and cervix carcinomas. Nat. Genet. 23, 18–20 (1999). 10471491 10.1038/12615

[b9] Sidransky D., Boyle J., Koch W. & van der Riet P. Oncogene mutations as intermediate markers. J. Cell. Biochem. Suppl. 17F, 184–187 (1993). 8412191 10.1002/jcb.240531026

[b10] Gartel A. L. & Radhakrishnan S. K. Lost in transcription: p21 repression, mechanisms, and consequences. Cancer Res. 65, 3980–3985 (2005). 15899785 10.1158/0008-5472.CAN-04-3995

[b11] Lianes P. *et al.* Altered patterns of MDM2 and TP53 expression in human bladder cancer. J. Natl Cancer Inst. 86, 1325–1330 (1994). 8064890 10.1093/jnci/86.17.1325

[b12] Lacombe L. *et al.* Analysis of p21WAF1/CIP1 in primary bladder tumors. Oncol. Res. 8, 409–414 (1996). 9114433

[b13] Cancer Genome Atlas Research Network. Comprehensive molecular characterization of urothelial bladder carcinoma. Nature 507, 315–322 (2014). 24476821 10.1038/nature12965 PMC3962515

[b14] Abbas T. & Dutta A. p21 in cancer: intricate networks and multiple activities. Nat. Rev. Cancer 9, 400–414 (2009). 19440234 10.1038/nrc2657 PMC2722839

[b15] Liu Y. & Bodmer W. F. Analysis of P53 mutations and their expression in 56 colorectal cancer cell lines. Proc. Natl Acad. Sci. USA 103, 976–981 (2006). 16418264 10.1073/pnas.0510146103 PMC1327731

[b16] Lu M. L. *et al.* Impact of alterations affecting the p53 pathway in bladder cancer on clinical outcome, assessed by conventional and array-based methods. Clin. Cancer Res. 8, 171–179 (2002). 11801555

[b17] Lawrence M. S. *et al.* Mutational heterogeneity in cancer and the search for new cancer-associated genes. Nature 499, 214–218 (2013). 23770567 10.1038/nature12213 PMC3919509

[b18] Lunter G. & Goodson M. Stampy: a statistical algorithm for sensitive and fast mapping of Illumina sequence reads. Genome Res. 21, 936–939 (2011). 20980556 10.1101/gr.111120.110 PMC3106326

[b19] Lise S. *et al.* Recessive mutations in SPTBN2 implicate β-III spectrin in both cognitive and motor development. PLoS Genet. 8, e1003074 (2012). 23236289 10.1371/journal.pgen.1003074 PMC3516553

[b20] Cazier J. B., Holmes C. C. & Broxholme J. GREVE: Genomic Recurrent Event ViEwer to assist the identification of patterns across individual cancer samples. Bioinformatics 28, 2981–2982 (2012). 22962342 10.1093/bioinformatics/bts547 PMC3496338

[b21] Yau C. *et al.* A statistical approach for detecting genomic aberrations in heterogeneous tumor samples from single nucleotide polymorphism genotyping data. Genome Biol. 11, R92 (2010). 20858232 10.1186/gb-2010-11-9-r92 PMC2965384

[b22] Roth A. *et al.* PyClone: statistical inference of clonal population structure in cancer. Nat. Methods 11, 396–398 (2014). 24633410 10.1038/nmeth.2883 PMC4864026

[b23] Gundem G. *et al.* IntOGen: integration and data mining of multidimensional oncogenomic data. Nat. Methods 7, 92–93 (2010). 20111033 10.1038/nmeth0210-92

