# Supplementary material for: Whole-genome sequencing of bladder cancers reveals somatic CDKN1A mutations and clinicopathological associations with mutation burden
Source: Nat Commun. 2014 Apr 29;5:3756. doi: 10.1038/ncomms4756 (PMC4010643; doi:10.1038/ncomms4756)
Supplement: Supplementary Figures, Tables and References — Supplementary Figures 1-7, Supplementary Tables 1-6 and Supplementary References [file ncomms4756-s1.pdf]

Supplementary Figures

Supplementary Figure 1. Somatic mutation spectra in our 14 samples compared with the four bladder cancer-specific signatures identified by Alexandrov *et al*[1].
The proportions of cancers with each of the 6 grouped SNV mutation types are shown for our 14 samples (all shown in black for clarity) and the Alexandrov *et al* signatures (dashed coloured lines). Our combined signature differs significantly from each of the Alexandrov signatures (P<0.01), but note some similarity to Signature 1b.

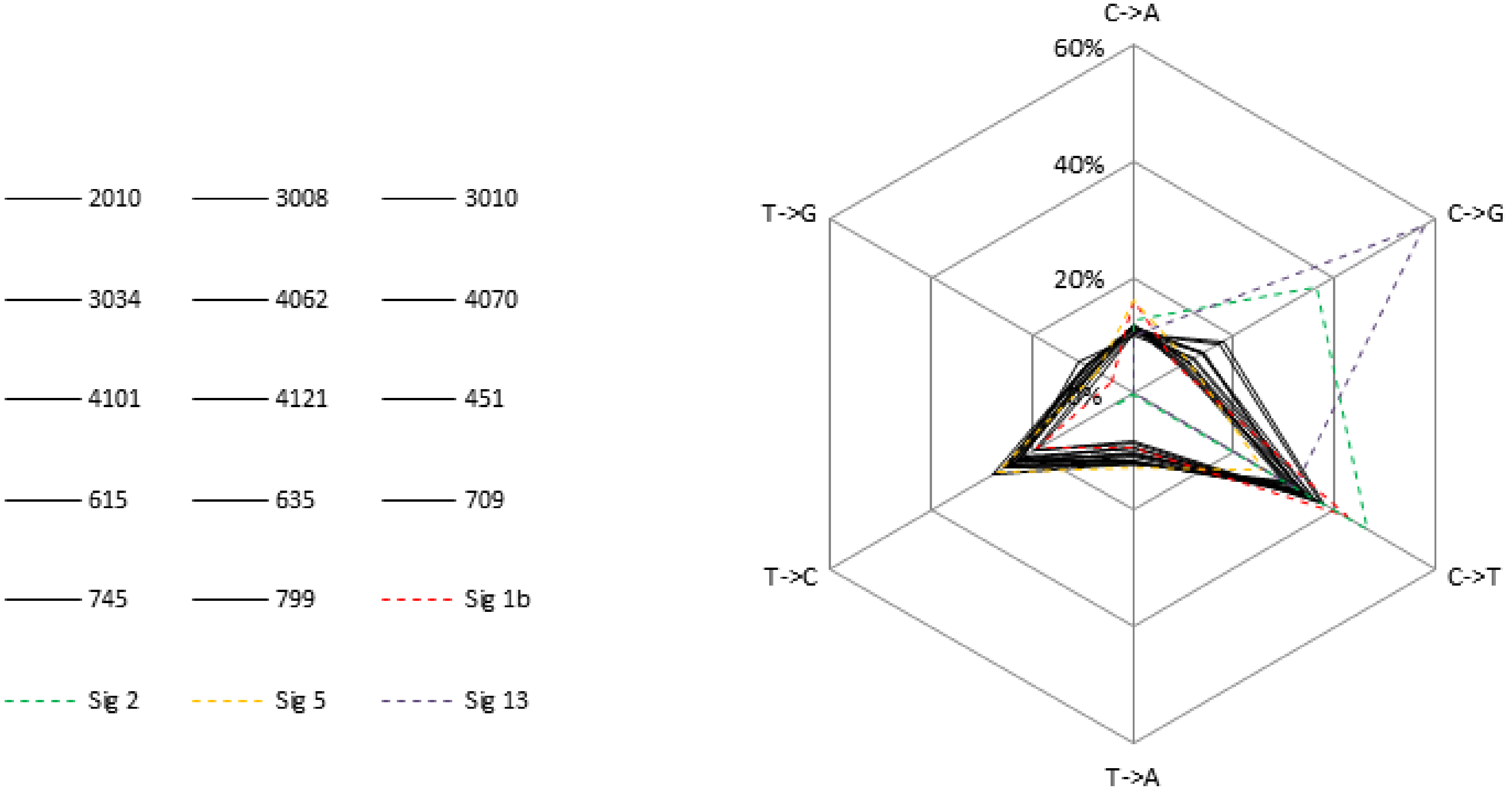

Supplementary Figure 2. Somatic mutation frequencies genome-wide

SNV (above zero) and indel (below zero) numbers (in 1Mb windows) are shown for each cancer with whole-genome sequence data.

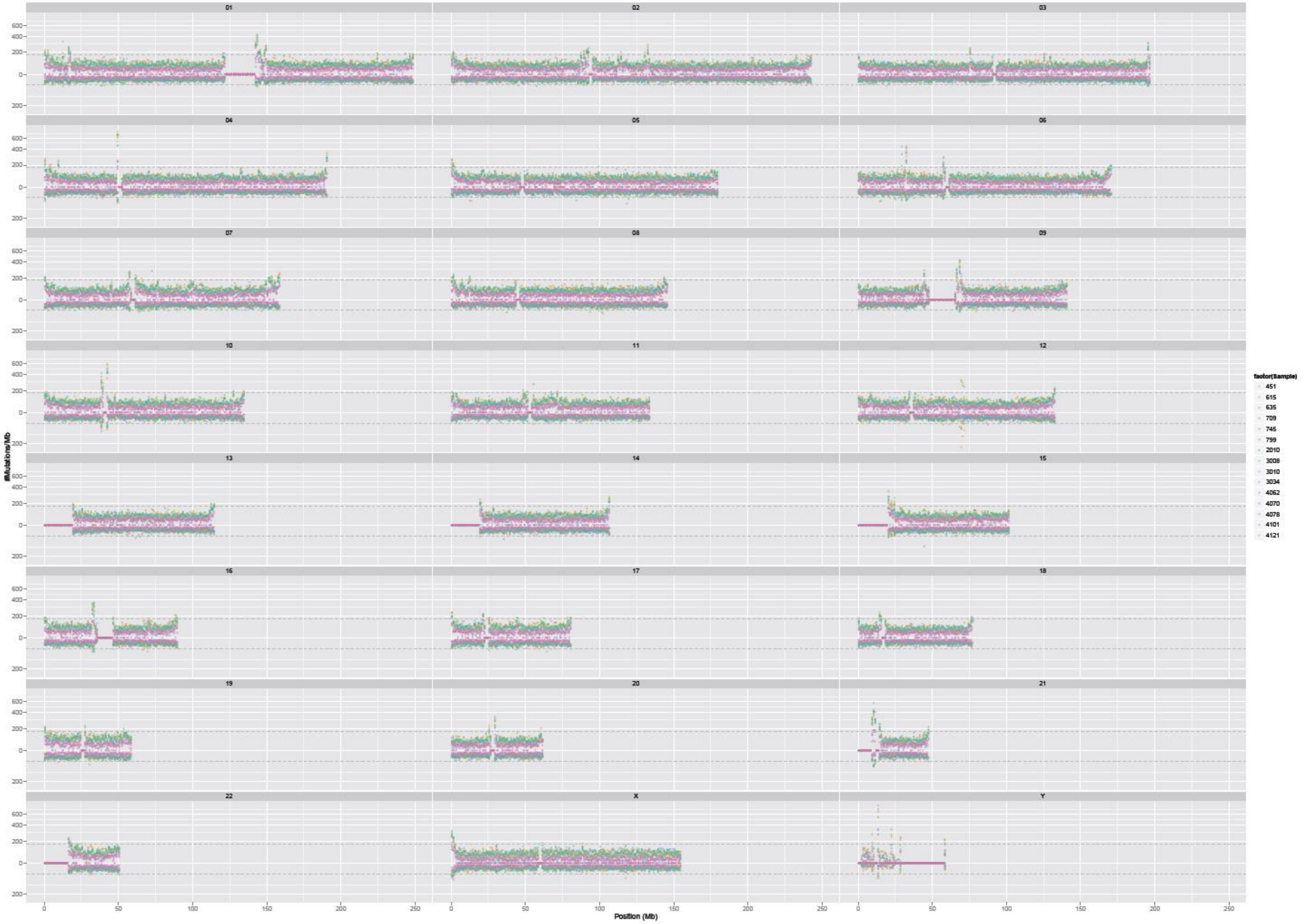

Supplementary Figure 3. Genome-wide summary of the copy number changes and loss of heterozygosity.

Copy number changes and LOH are summarised across the genome-wide sequenced discovery set samples, as generated with GREVE[2]. Each sample is represented by a vertical bar: Loss (red), Gain (green), Copy Neutral LOH (blue). Samples are shown as per the order in Table 1, from the ideogram outward.

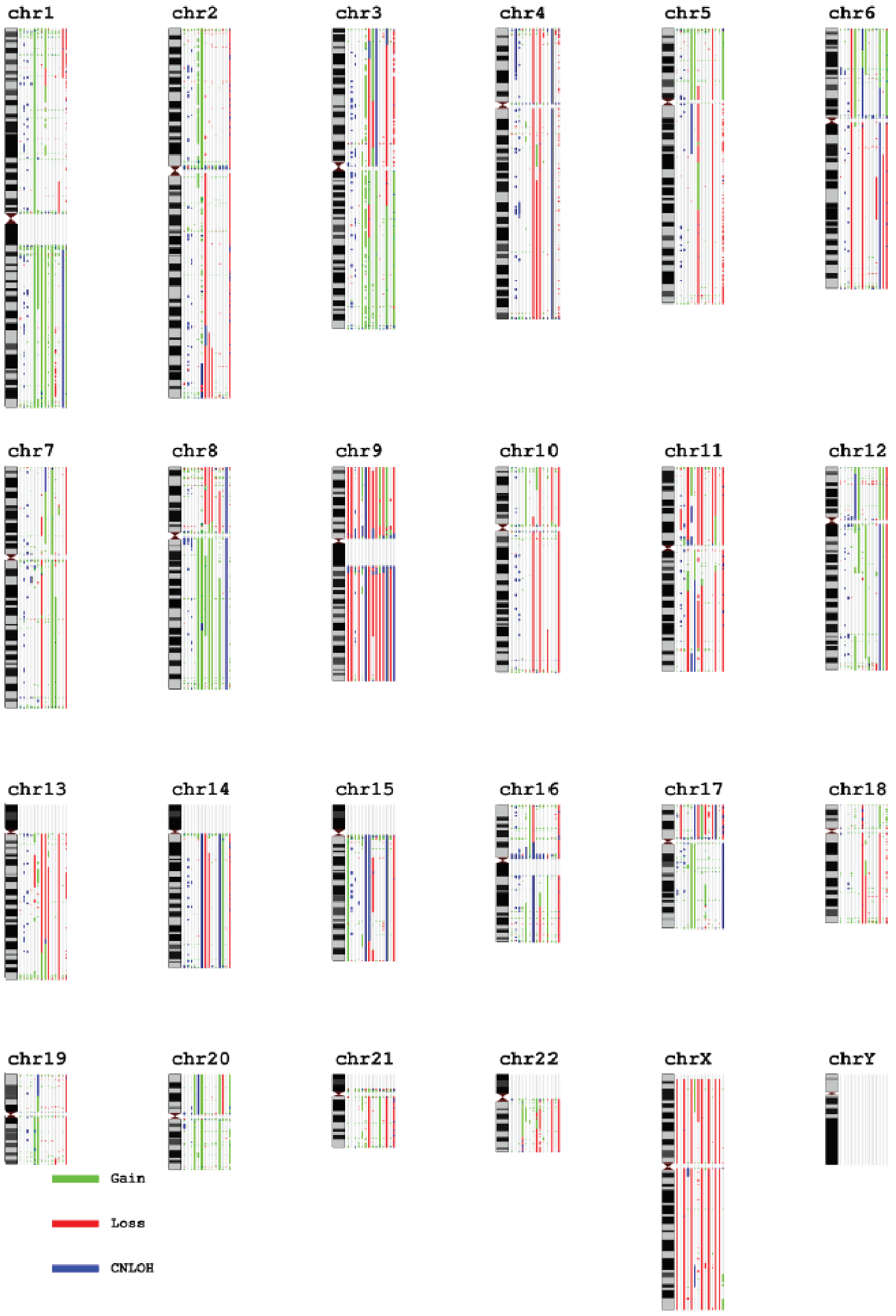

Supplementary Figure 4. Examples of chromothripsis.

Genome studio output is shown for chromosome arms 3p (#745)

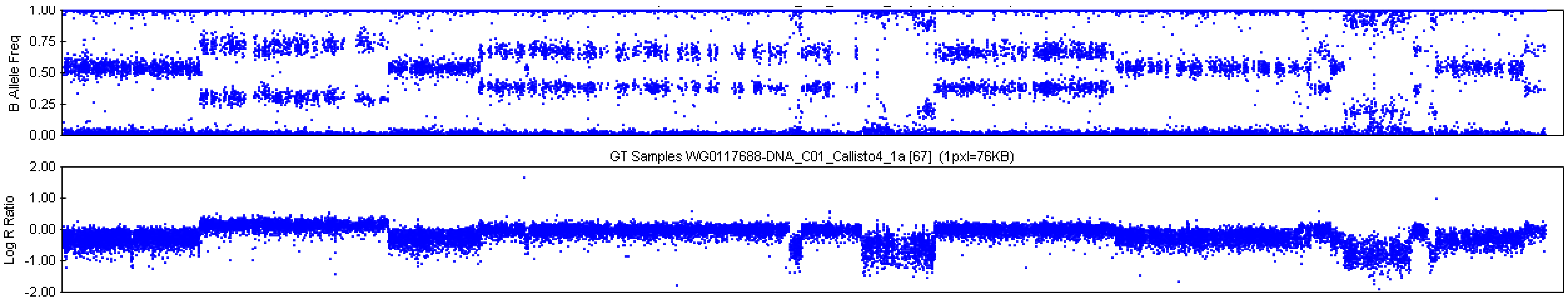

and 9p (#799)

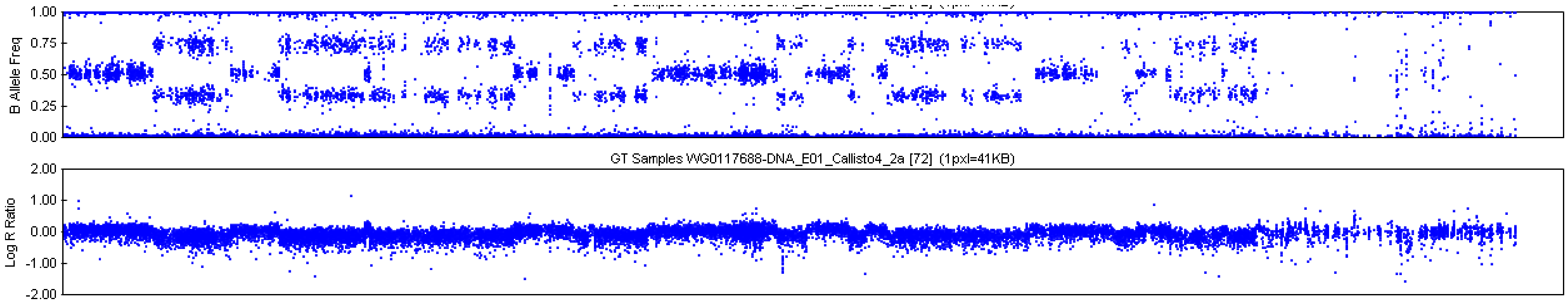

Supplementary Figure 5. Splicing defect caused by *STAG2* mutation in sample #2056.

The image shows a ~250bp PCR product from duplicate assessments of sample #2056 in lanes 2 and 3. Lane 4 is a reduced loading of #2056 in which the 257bp product falls below detection threshold. Two wildtype control cancers analysed alongside #2056 are shown in lanes 5 and 6, with 100bp ladder in lanes 1 and 7. The ~250bp product corresponds to the expected size if splicing of intron 18 fails owing to the STAG2 splice site mutation (that is, 136bp trans-intron cDNA amplicon + 121bp intron = 257bp). This intron retention is predicted to lead to a premature STOP codon 13 amino acids after the upstream splice site. The proportion of mutant *STAG2* transcripts affected in this way is difficult to assess given the presence of contaminating normal cells in the tumour and the presence of the mutation in a heterozygous, and potentially aneusomic, state, but it appears that splicing efficiency is reduced rather than abolished.

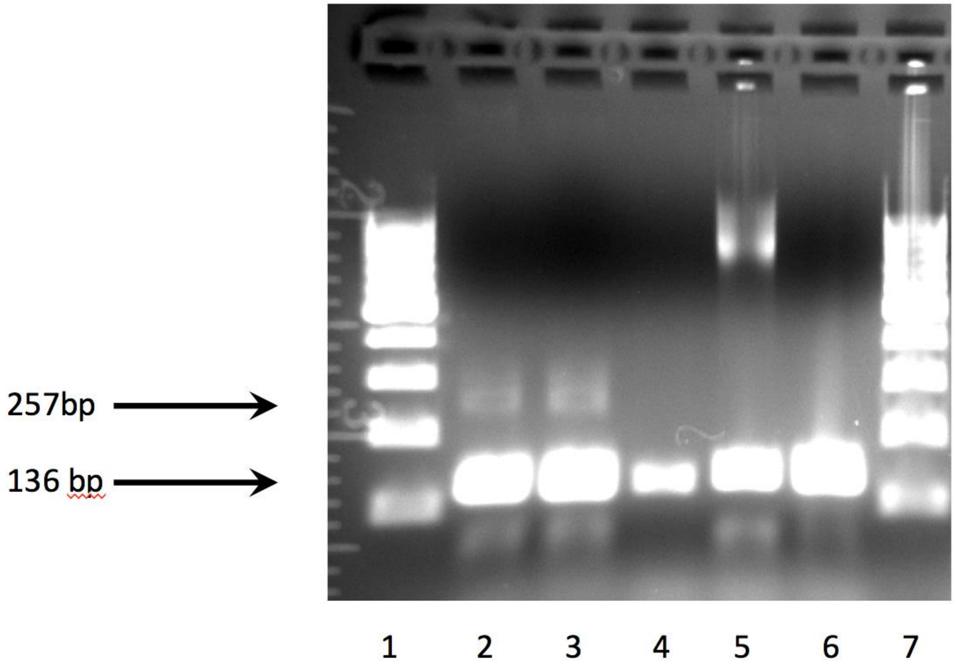

Supplementary Figure 6. Intogen pathway analysis

Top pathways in the list of somatic mutations in our 15 samples with whole-genome or exome sequence data as calculated by IntoGen (http://www.intogen.org/).

pathway	fm-bias	found/studied	mut-freq
Bladder cancer	1.914E-10	13 / 14	0.929
Wnt signaling pathway	1.528E-7	11 / 14	0.786
Adherens junction	1.528E-7	12 / 14	0.857
Notch signaling pathway	2.835E-7	11 / 14	0.786
Hematopoietic cell lineage	9.996E-7	12 / 14	0.857
Pathways in cancer	1.096E-6	14 / 14	1
PI3K-Akt signaling pathway	2.25E-6	14 / 14	1
HTLV-I infection	2.305E-6	14 / 14	1
Chronic myeloid leukemia	4.547E-6	12 / 14	0.857
Transcriptional misregulation in cancer	6.415E-6	12 / 14	0.857
Epstein-Barr virus infection	6.415E-6	14 / 14	1
Regulation of actin cytoskeleton	8.125E-6	14 / 14	1
Tight junction	1.596E-5	13 / 14	0.929
Focal adhesion	1.831E-5	13 / 14	0.929
Viral carcinogenesis	2.333E-5	14 / 14	1
Melanoma	4.252E-5	13 / 14	0.929
Melanogenesis	6.325E-5	11 / 14	0.786
TGF-beta signaling pathway	8.027E-5	12 / 14	0.857
Endocytosis	9.142E-5	14 / 14	1
Glioma	9.147E-5	11 / 14	0.786

Supplementary Figure 7. Immunohistochemical detection of p21 and p53 in two pT2G3 bladder tumours.

Patients studied using immunohistochemistry were as described in Choudhury et al[3] with the addition of one further patient. Here, Patient A's tumour shows few positive nuclei (7%) for p21 but strong nuclear p53 staining in 97% of tumour cells. Unstained cells in p53 section are stromal. Patient B's tumours shows 90% positive nuclear staining for p21 but only 3% of tumour cells positive for p53. Magnification x100, scale bar = 100 μM

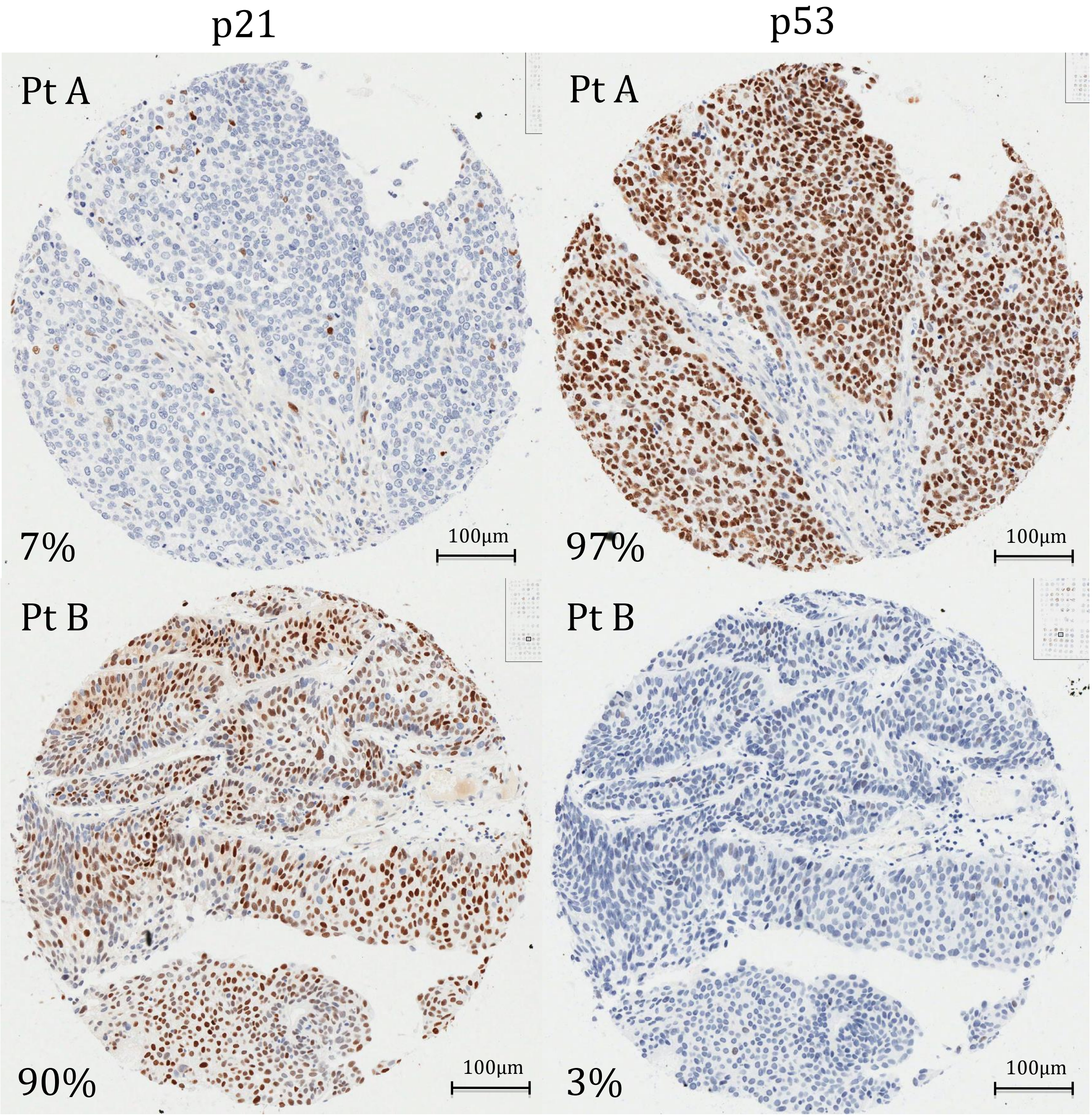

Supplementary Tables

Supplementary Table 1. Full summary genome data from the Discovery set of cancers.

Columns 1-2: Sample ID and pathological stage. Column 3: Percentage of tumour cells in the sample. Columns 4 and 5: the number of variants from the reference sequence (SNVs plus indels) observed exome- (EM) and genome-wide (GM). Columns 6 and 7: the number of somatic mutations (SNVs plus indels) observed exome- (ESM) and genome-wide (GSM). Columns 8 and 9: the number of CNVs and the proportion of the genome affected by any CNV. Columns 10-11: the number of filtered putative structural change breakpoints (#BP) and genic (#GBP).

1	2	3	4	5	6	7	8	9	10	11
**Sample**	**Type**	**Tumour%**	**GM**	**EM**	**ESM**	**GSM**	**No. CNVs**	**Size (%)**	**#BP**	**#GBP**
**4062**	pTa	70	4,626,578	57,461	146	34,966	654	14%	940	30
**4070**	pTa	90	4,622,984	55,236	137	33,861	658	14%	1179	25
**4101**	pTa	80	4,665,347	52,990	154	32,608	669	12%	1097	20
**4121**	pTa	70	4,654,880	63,815	235	40,678	729	19%	1126	32
**635**	pT1	70	5,075,728	147,005	920	113,970	633	42%	1892	25
**709**	pT1	70	5,106,213	144,277	541	110,745	741	39%	3011	86
**745**	pT1	80	5,063,585	127,627	427	122,353	888	67%	2685	28
**799**	pT1	80	5,115,178	131,560	272	123,701	629	47%	618	18
**3010**	pT1	90	5,131,426	128,346	350	139,673	812	33%	2562	55
**451**	pT2	90	5,047,283	120,027	385	111,643	644	20%	3550	70
**615**	pT2	90	5,116,797	126,941	892	108,578	703	31%	2101	31
**2010**	pT3	70	5,139,498	129,465	482	122,547	710	53%	2088	46
**3008**	pT3	80	5,090,404	135,219	402	117,965	557	24%	2594	66
**3034**	pT3	80	5,089,153	110,346	495	99,990	1233	69%	1671	32

Supplementary Table 2. Genes present in homozygously deleted regions

*=regarded as principal target of deletion in this study based on literature

Tumour ID	Chromosome	Position (Mb)	Genes
451	4	2.7-5.7	TNIP2, SH3BP2, GRK4, STK32B & others
	5	13.4-13.8	DNAH5, TRIO, ANKH
	9	21.8-22.3	MTAP, CDKN2A*, CDKN2B
615	3	4.6-5.7	ITPR1, ARL8B, EDEM1
	4	186.1-188.2	FAT1*
745	4	22.9-25.2	PPARGC1A, DHX15, LGI2 & others
	9	3.2-3.7	RFX3
	11	111.3-119.8	NCAM1, CADM1, CXCR5 & others
3008	9	21.4-22.2	MTAP, CDKN2A*, CDKN2B

Supplementary Table 3. Replication set clinical data

Sample	Age	Gender	Smoking	Occupation; exposure	Stage	Grade	Growth pattern	treatment
2060	61	m	ex-	Civil Servant	pTa	G1	Papillary	
1988	62	m	ex-	Manager	pTa	G1	Papillary	
2072	84	m	ex-	Bus driver car delivery; diesel fumes 20yrs	pTa	G1	Papillary	
2073	65	m	ex-	Builder	pTa	G1	Papillary	
1954	66	m	ex-	Painter decorator; paints/dyes 40yrs diesel fumes 7yrs	pTa	G1	Papillary	BCG
2056	74	f	ex-	Factory Worker	pTa	G1	Papillary	
2074	86	m	ex-	School Master	pTa	G1-2 LG	Papillary	
2071	81	m	ex-	Maintenance; asbestos	pTa	G1-2 LG	Papillary	
1942	86	f	ex-	Blanket Factory worker 55yrs	pTa	G2	Papillary	
1991	53	f	ex-	Petrol station worker 20yrs	pTa	G2	Papillary	
2070	90	f	never	Shop assistant/cleaner	pTa	G2	Papillary	
2053	62	f	ex-	Admin	pTa	G2	Papillary	
2051	69	m	ex-	Barbershop owner 50yrs	pTa	G2	Papillary	
1937	88	m	ex-	Gardener; Diesel fumes	pTa	G2 HG	Papillary	
2077	81	m	ex-	Chemical Plant Designer; chemicals	pTa	G3	Papillary/Solid	
2061	84	m	never	Wine Trader 25yrs	pTis	G3	Papillary	
1941	63	m	current	Accountant; Rubber	pT1	G2	Papillary	
1987	61	m	ex-	Company Director	pT1	G2	Papillary	
2050	79	m	ex-	Carpenter	pT1	G2	Papillary	
1961	84	m	never	Shop Proprietor	pT1	G3	Papillary	
2062	58	m	never	Garage receptionist; dyes, paints, diesel fumes	pT1	G3	Papillary	cystectomy
2065	60	m	ex-	Car mechanic taxi driver; diesel fumes 38 yrs	pT1	G3	Sarcomatoid TCC	
1992	62	m	ex-	Head Artist	pT1	G3	Papillary/Solid	
2058	69	m	never	Painter decorator; paints and dyes	pT1	G3	Papillary/Solid	
2054	55	m	ex-	Carpenter; plastics	pT1	G3	Solid	
2063	87	m	ex-	Bank Manager	pT1	G3	Papillary	
2049	76	m	ex-	Driving instructor; diesel fumes 30yrs	pT2	G3	Not stated	
1985	59	m	NK	NK	pT2	G3	Papillary	cystectomy
2067	69	m	ex-	Firefighter	pT2	G3	Papillary	
2055	67	m	ex-	Computer engineer	pT2	G3	Solid	
1947	85	m	ex-	Car Factory Worker	pT2	G3	Papillary/Solid	
1959	56	m	never	Window Cleaner	pT2	G3	Solid Squamous cell	
2052	81	f	ex-	Civil Servant	pT2	G3	Solid Squamous cell	
1938	84	m	ex-	Driver	pT2	G3	Papillary	
2057	67	m	never	Commercial Manager	pT2	G3	Solid	

Supplementary Table 4. *CDKN1A, TP53* and *STAG2* mutations in the replication set

**Gene**	**Sample#**	**Mutation site**	SIFT	Polyphen2
CDKN1A	2077	p.Met1Val	0.02	0.064
CDKN1A	1961	p.Cys39X		
CDKN1A	2056	p.Gly139Cys	0.10	0.947
CDKN1A	1992	p.Tyr77del		
TP53	1992	p.Arg213Pro	0.00	1.000
TP53	2076	p.Arg213Pro	0.00	1.000
TP53	1938	p.Ser215Thr	0.00	1.000
TP53	2065	p.Glu331X		
STAG2	2056	c.1732-1 G>T		

Supplementary Table 5. Association between p53 and p21 protein expression. Positive expression of either protein was scored using a cut-off of 20% of tumour cell nuclei stained. Number of cancers in each category are shown.

		p21	
		-	+
p53	-	6	6
	+	14	21

P=0.74, N=47, Fisher's exact test

Supplementary Table 6. PCR primers for validation and replication of mutations in STAG2 and CDKN1A.

All primers were for Sanger sequencing except the last pair, which were used to check the effect of the *STAG2* splice site mutation.

CDKN1A-exon2-part1-F	CTGAGGTGACACAGCAAAGC
CDKN1A-exon2-part1-R	CTGCCTCCTCCCAACTCAT
CDKN1A-exon2-part2-F	GACACCACTGGAGGGTGACT
CDKN1A-exon2-part2-R	TCTGGGCCTGTTTCCTTCTA
CDKN1A-exon2-mid-F	GCGACTGTGATGCGCTAAT
CDKN1A-exon2-mid-R	CAGGTCCACATGGTCTTCCT
CDKN1A-exon3-part1-F	CCCCTCAAGAGACAGAGTGG
CDKN1A-exon3-part1-R	GCCAGGGTATGTACATGAGGA
STAG2-exon8-F	TCATGCATTCTAAATGAAATTGCT
STAG2-exon8-R	TCATCTCAAATCTAAGACAATATGCAG
STAG2-exon9-F	TTGTGTCTGTTAGATTAGTTTCACCA
STAG2-exon9-R	CAAGTTGGTCACACAATAGCC
STAG2-exon10-F	GGTGTTCATTTGGTTGTCTTCC
STAG2-exon10-R	TCCCTACACCACGAAATATGC
STAG2-exon11-F	GTTTTTCCTTCCCCCATTCA
STAG2-exon11-R	CACGCCGATGTGTAGAAAGA
STAG2-exon17-F	TGGGATGCTGAGGGTTTTAG
STAG2-exon17-R	TTGTTTCATGGCTAAAATGGAA
STAG2-exon18-F	TGAAATTTTTGTGTCCATCTCTT
STAG2-exon18-R	CTGAGGCAACTGCAACAAGT
STAG2-exon19-F	CGTGGCCCTTCCTCAGTTAT
STAG2-exon19-R	GGTTTCCTTTCTTAAAATACGTTCC
STAG2-exon20-F	TTTCCATGGTGGTATGGTCA
STAG2-exon20-R	TGCTAGGGACTATCACCAAGAC
STAG2-exon21-F	CCCAGCCATATTGCCTTAAA
STAG2-exon21-R	CTTTAAAATGCCCCCACAAC
STAG2-exon22-F	TGCATGTTTTGTGGGTTATG
STAG2-exon22-R	TGCAGTGCGTGAATAACAATC
STAG2-exon23-F	TGACCTTTCAAAGTGGGATTT
STAG2-exon23-R	AGAAACAAGCAACATTTCACTG
STAG2-exon24-F	TGCAGAAGTAGAGTTAATAAAGCTATG
STAG2-exon24-R	CAAGATATTTCTGCTTTGCTCA
STAG2-exon25-F	GTTGGATTCTGTTTCTGTGTTAAA
STAG2-exon25-R	CCTGTGTTTCACGTTTTTAAGG
STAG2-E18/19INT-F	CAGCAAAGGAGAAGAAGACACA
STAG2-E18/19INT-R	TACTGAGGCAACTGCAACAA

Supplementary References

1. Alexandrov, L.B. et al. Signatures of mutational processes in human cancer. Nature 500, 415-21 (2013).
2. Cazier, J.B., Holmes, C.C. & Broxholme, J. GREVE: Genomic Recurrent Event ViEwer to assist the identification of patterns across individual cancer samples. Bioinformatics 28, 2981-2 (2012).
3. Choudhury, A. et al. MRE11 expression is predictive of cause-specific survival following radical radiotherapy for muscle-invasive bladder cancer. Cancer Res., 70, 7017-26. (2010)
