# Supplementary material for: Whole-genome sequencing of bladder cancers reveals somatic CDKN1A mutations and clinicopathological associations with mutation burden
Source: Nat Commun. 2014 Apr 29;5:3756. doi: 10.1038/ncomms4756 (PMC4010643; doi:10.1038/ncomms4756)

**Supplementary Data 1. OncoSNP-Seq analysis**

Copy number variation and loss of heterozygosity are shown for each of the discovery set samples with whole-genome sequence, as identified by OncoSNP-Seq for the most likely model. The first row shows the average coverage across the genome. The second row contains the allelic ratio at each of the selected Single Nucleotide Variant. The following rows present the inferred copy number at increasingly finer scale.
See Yau, C. *et al*. OncoSNP-SEQ: a statistical approach for the identification of somatic copy number alterations from next-generation sequencing of cancer genomes. *Bioinformatics* ***29,*** *2482-4* (2013)

4062

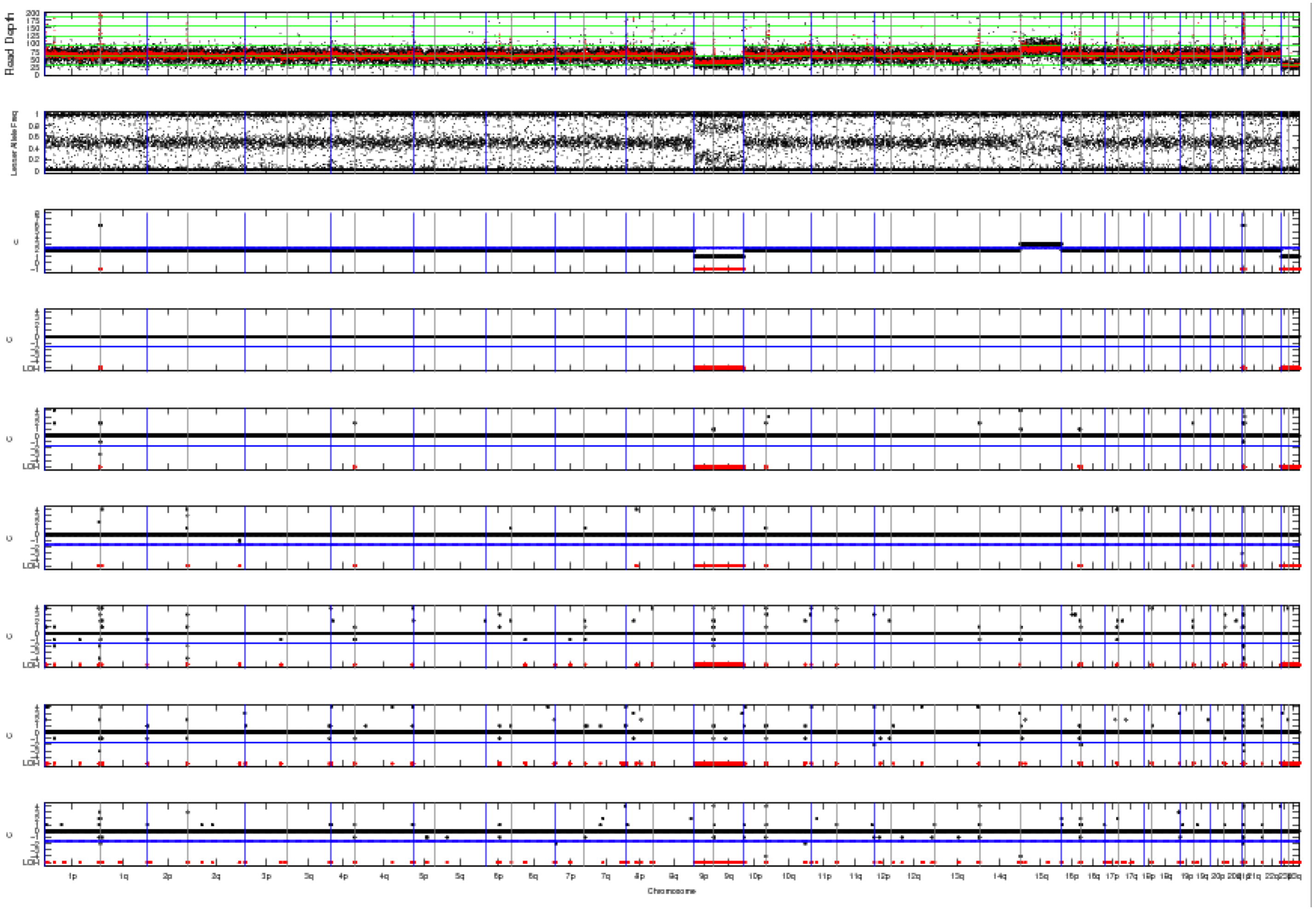

4070

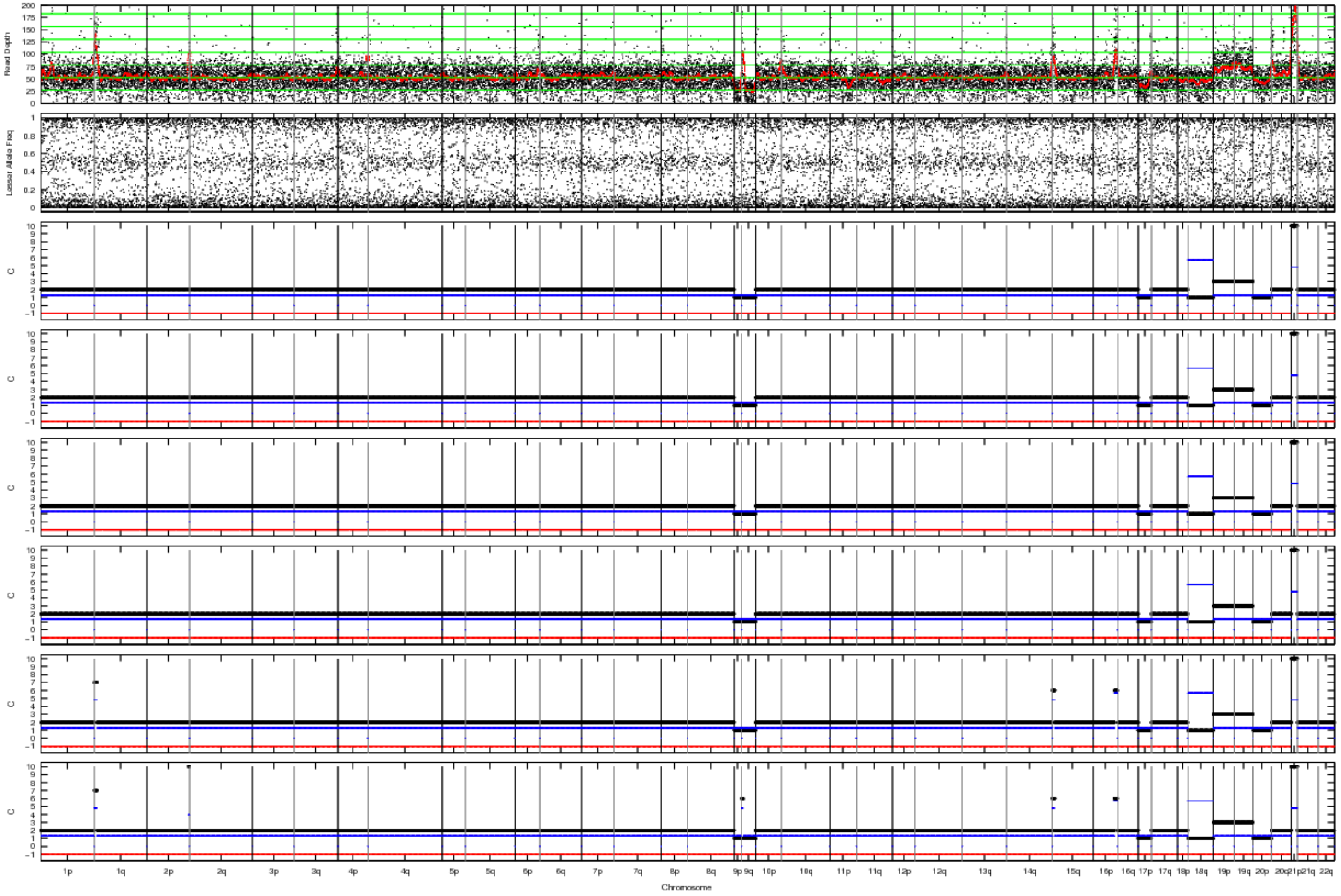

Read Depth
Lesser Allele Freq
C
1p 1q 2p 2q 3p 3q 4p 4q 5p 5q 6p 6q 7p 7q 8p 8q 9p 9q 10p 10q 11p 11q 12p 12q 13q 14q 15q 16p 16q 17p 17q 18p 18q 19p 19q 20p 20q 21p 21q 22q
Chromosome

4101

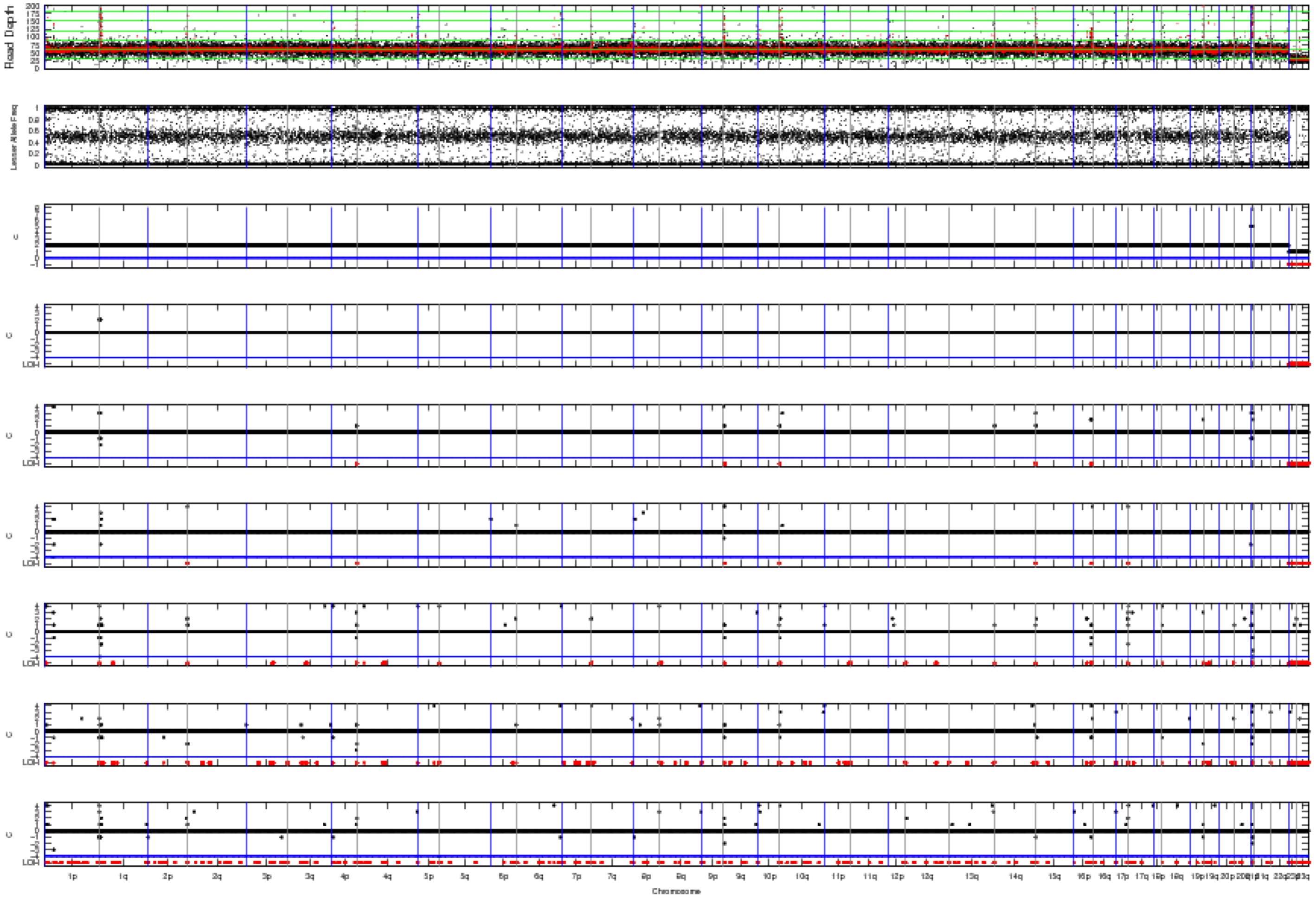

4121

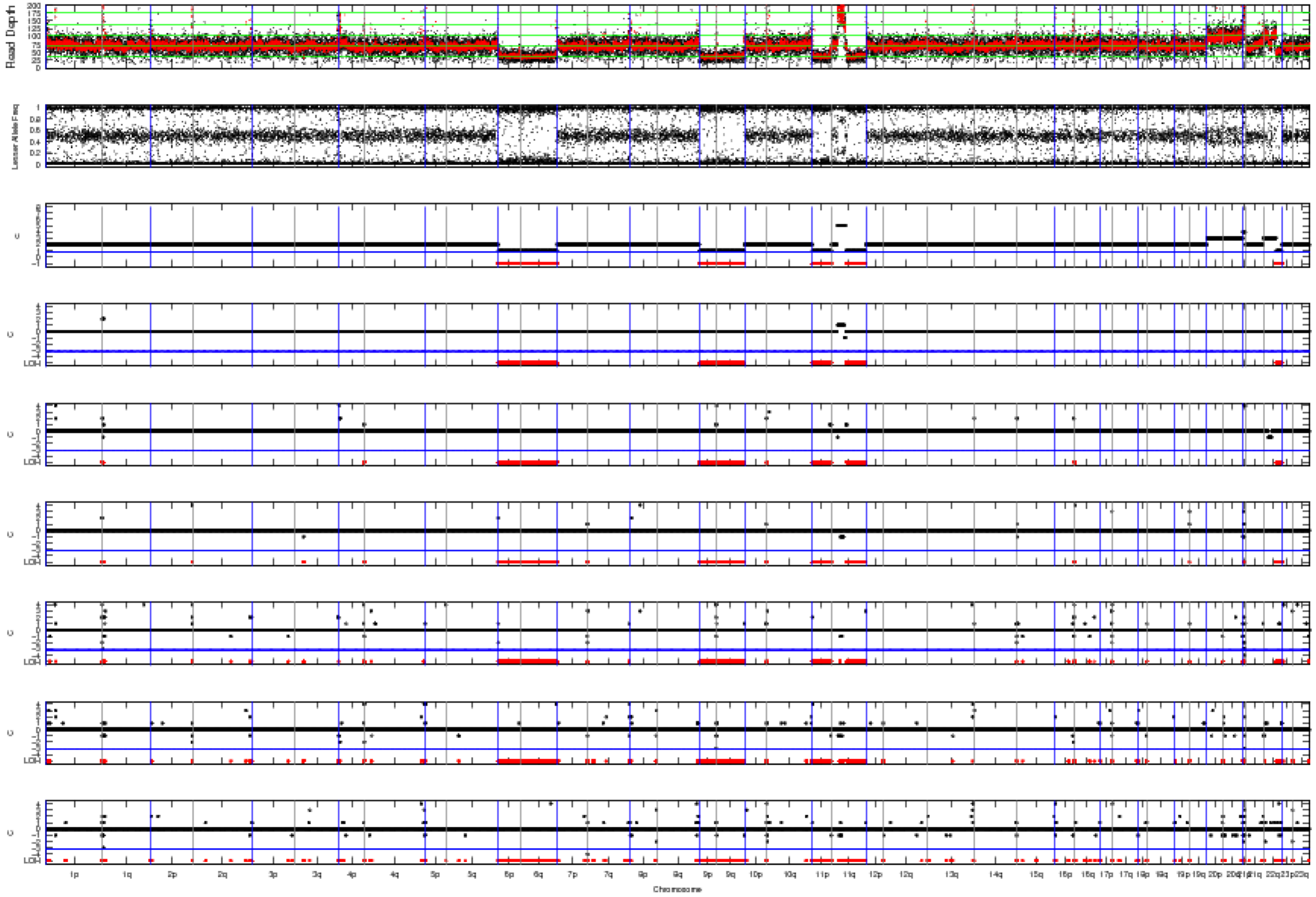

635

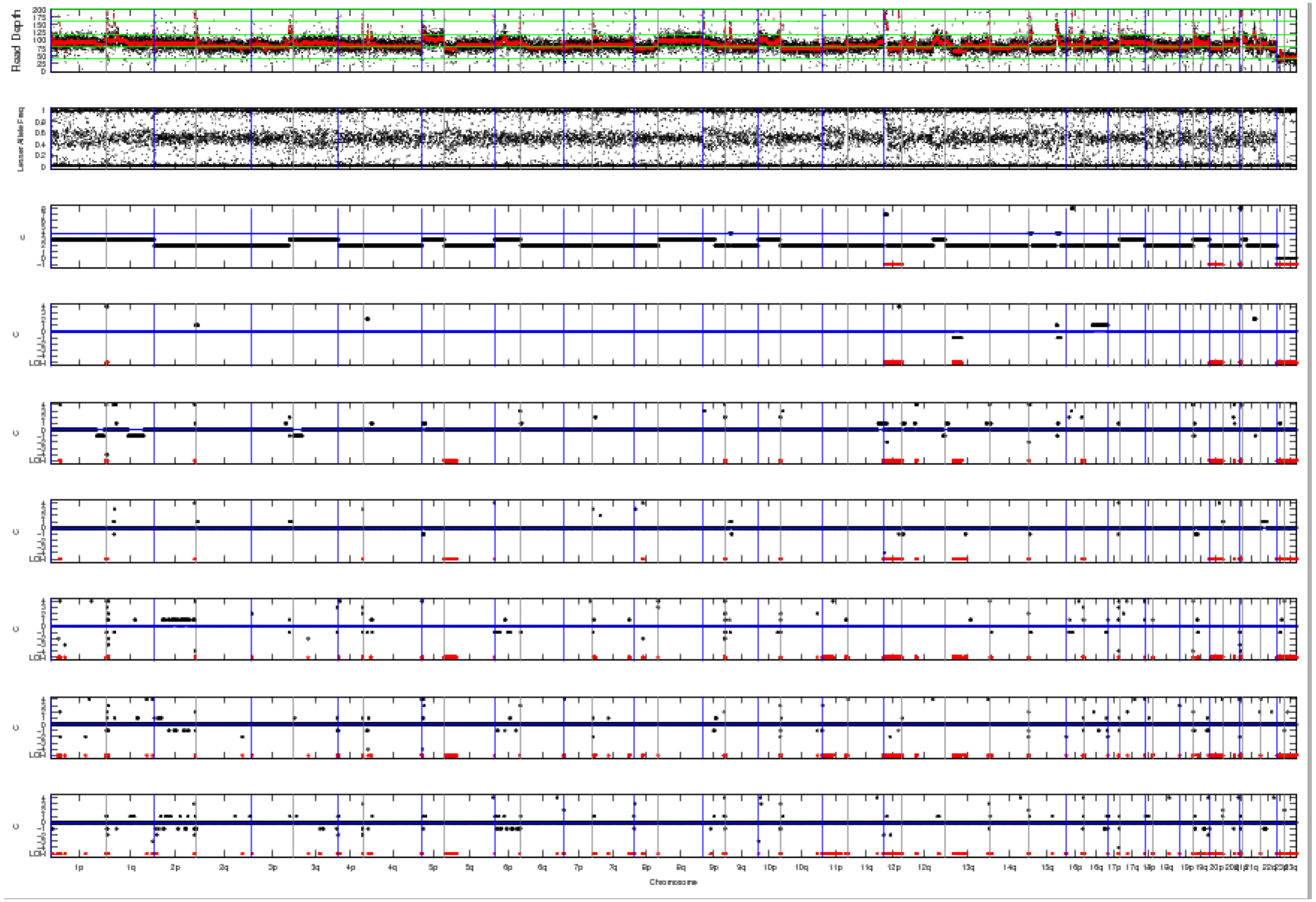

709

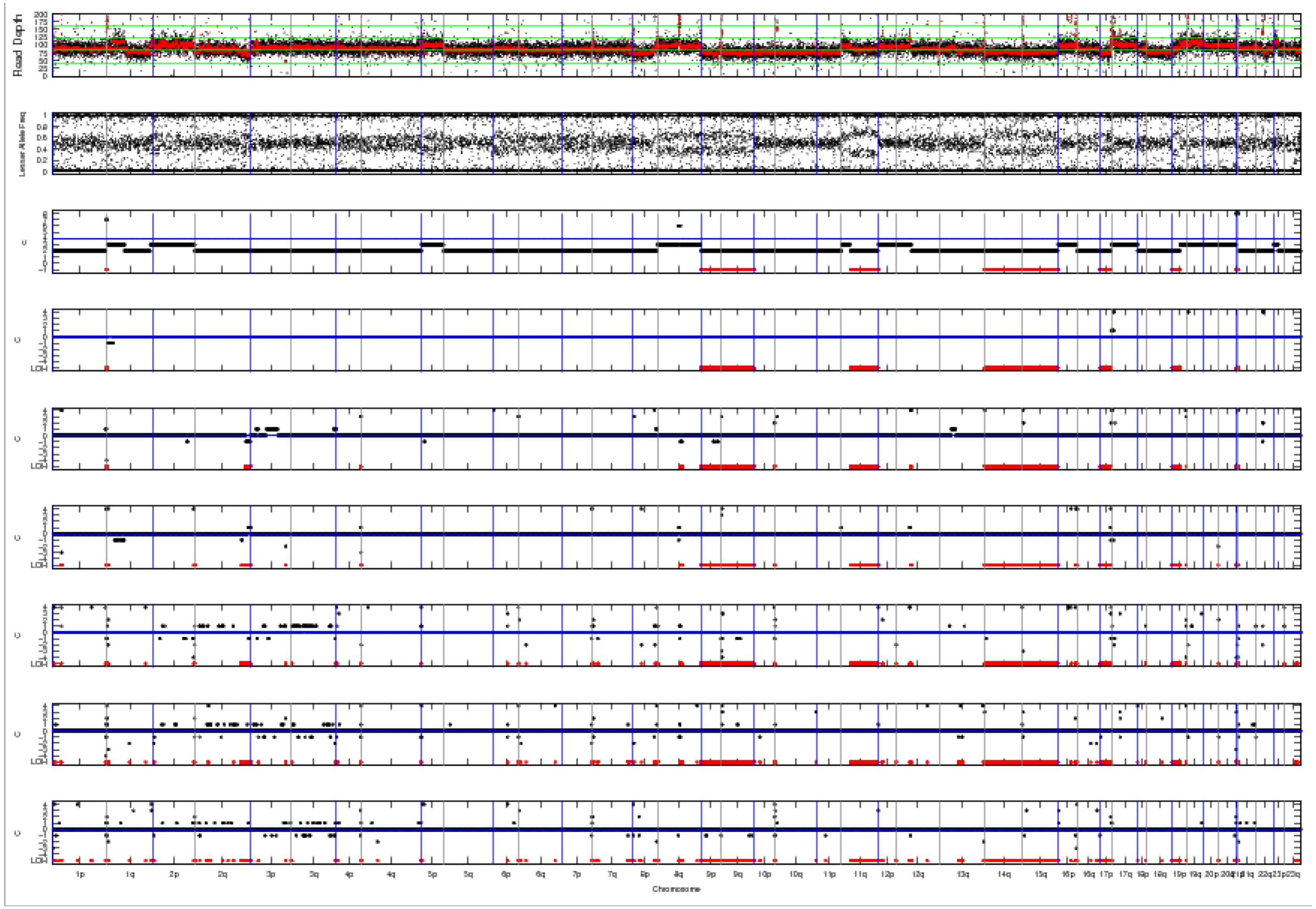

Read Depth
Lesser Allele Freq
1p
1q
2p
2q
3p
3q
4p
4q
5p
5q
6p
6q
7p
7q
8p
8q
9p
9q
10p
10q
11p
11q
12p
12q
13q
14q
15q
16p
16q
17p
17q
18p
18q
19p
19q
20p
21q
22q
23p
23q
Chromosome

745

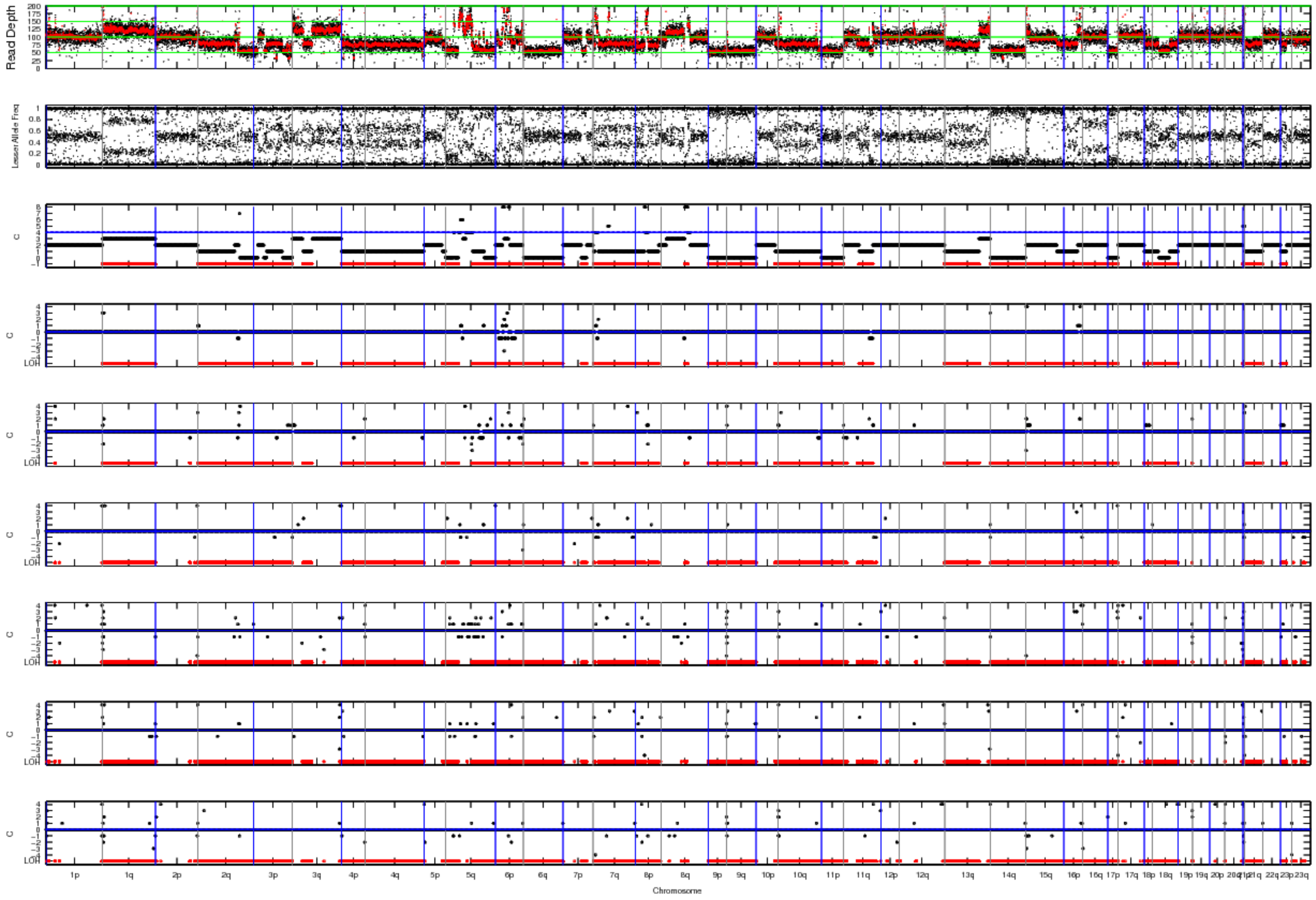

Read Depth
Lesser Allele Freq
Chromosome
1p
1q
2p
2q
3p
3q
4p
4q
5p
5q
6p
6q
7p
7q
8p
8q
9p
9q
10p
10q
11p
11q
12p
12q
13q
14q
15q
16p
16q
17p
17q
18p
18q
19p
19q
20p
21q
22q
23p
23q
LOH

799

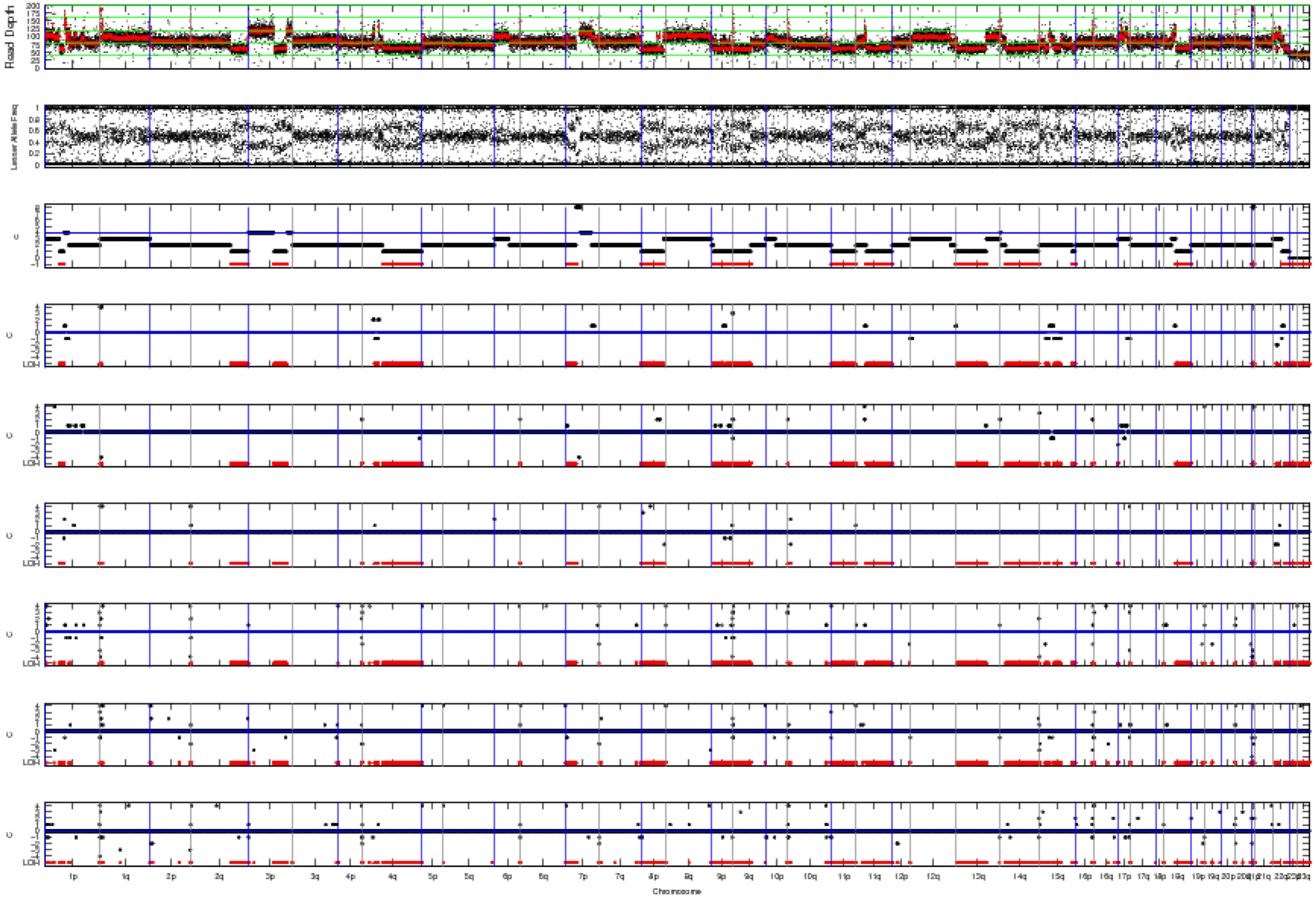

3010

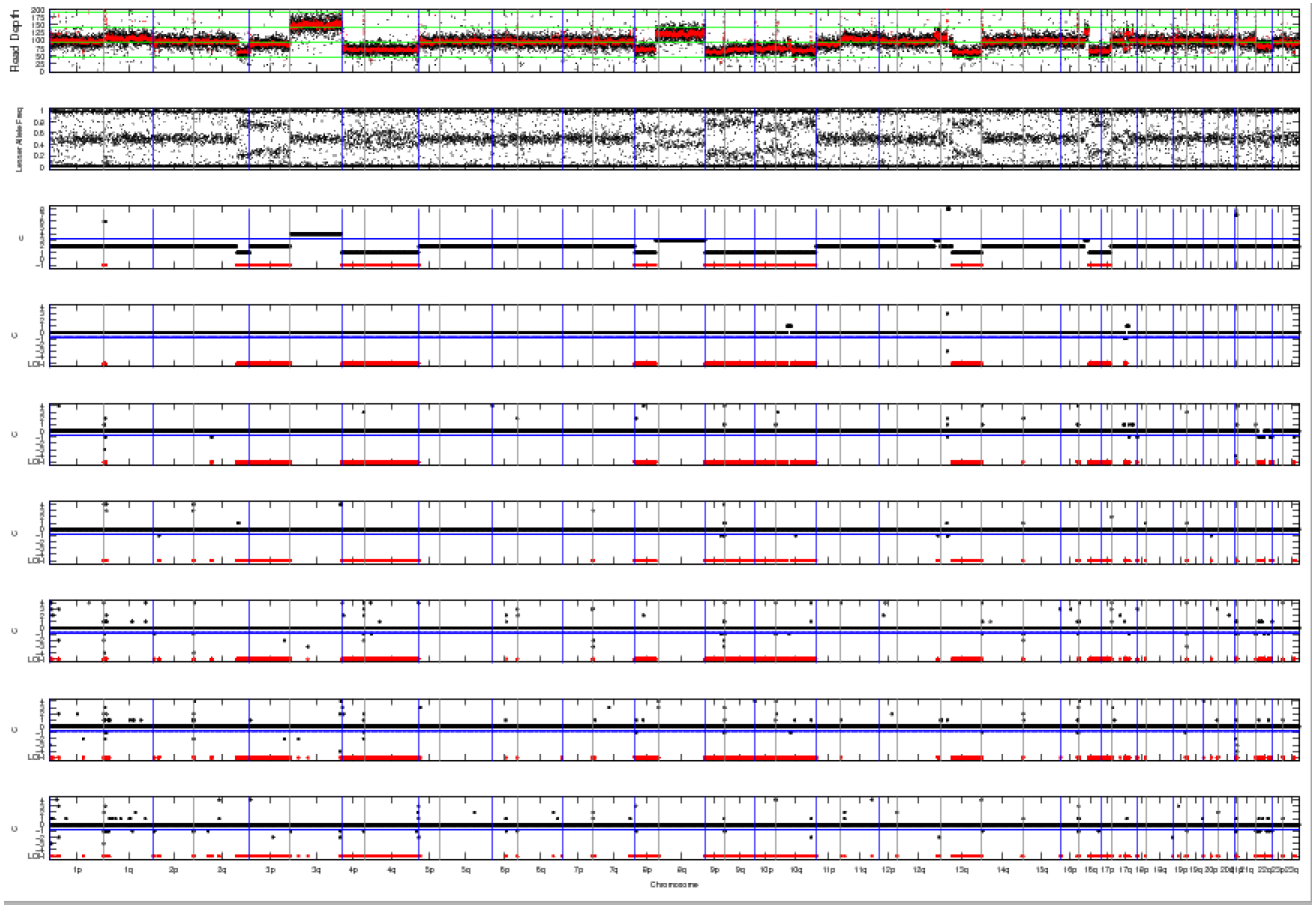
Read Depth
Lesser Allele Freq
Chromosome
1p
1q
2p
2q
3p
3q
4p
4q
5p
5q
6p
6q
7p
7q
8p
8q
9p
9q
10p
10q
11p
11q
12p
12q
13q
14q
15q
16p
16q
17p
17q
18p
18q
19p
19q
20p
20q
21q
22q

451

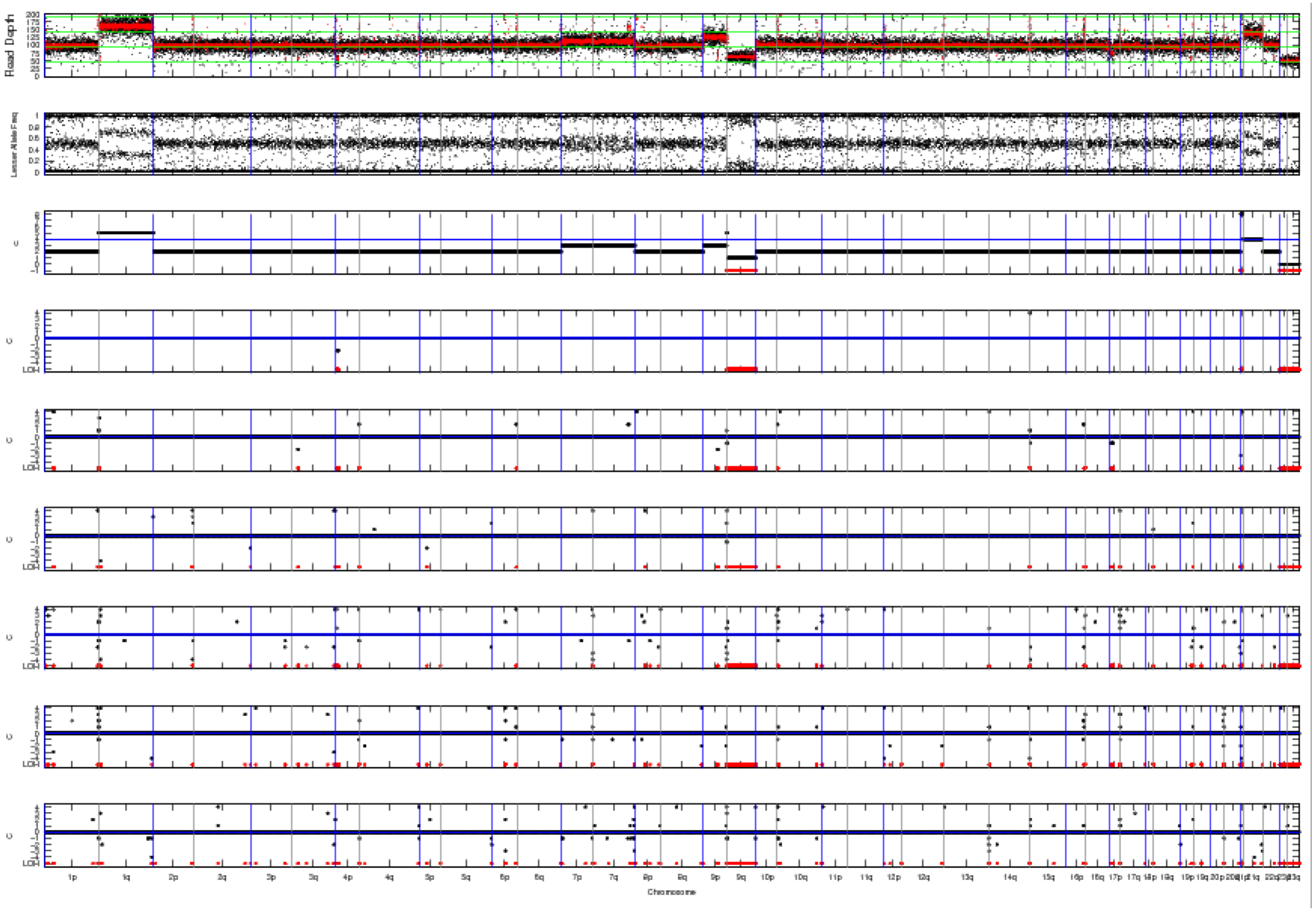
Read Depth
Lesser Allele Freq
Chromosome
1p
1q
2p
2q
3p
3q
4p
4q
5p
5q
6p
6q
7p
7q
8p
8q
9p
9q
10p
10q
11p
11q
12p
12q
13q
14q
15q
16p
16q
17p
17q
18p
18q
19p
19q
20p
22q

615

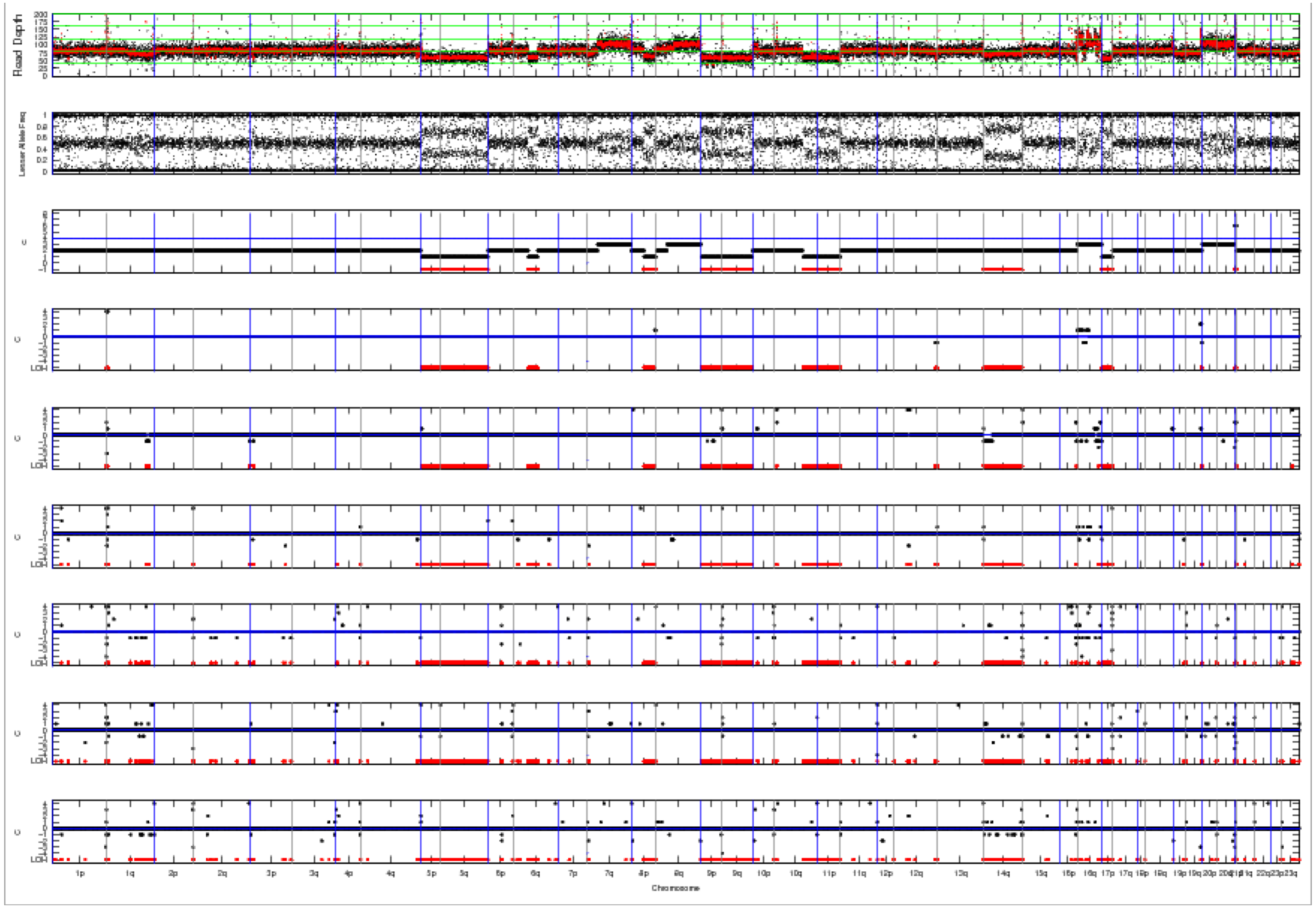

Read Depth
Lesser Allele Freq
1p
1q
2p
2q
3p
3q
4p
4q
5p
5q
6p
6q
7p
7q
8p
8q
9p
9q
10p
10q
11p
11q
12p
12q
13q
14q
15q
16p
16q
17p
17q
18p
18q
19p
19q
20p
Chromosome

2010

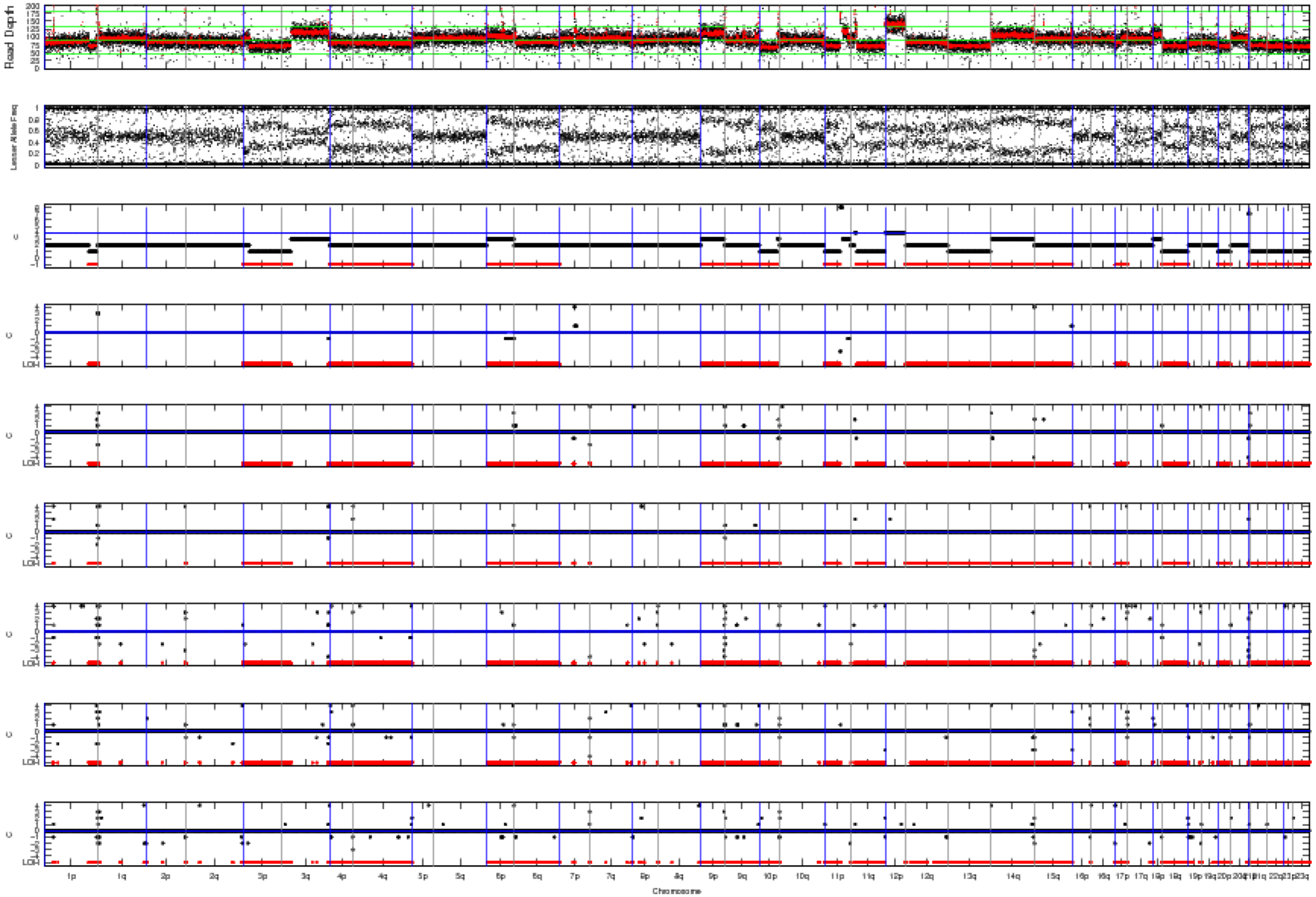

3008

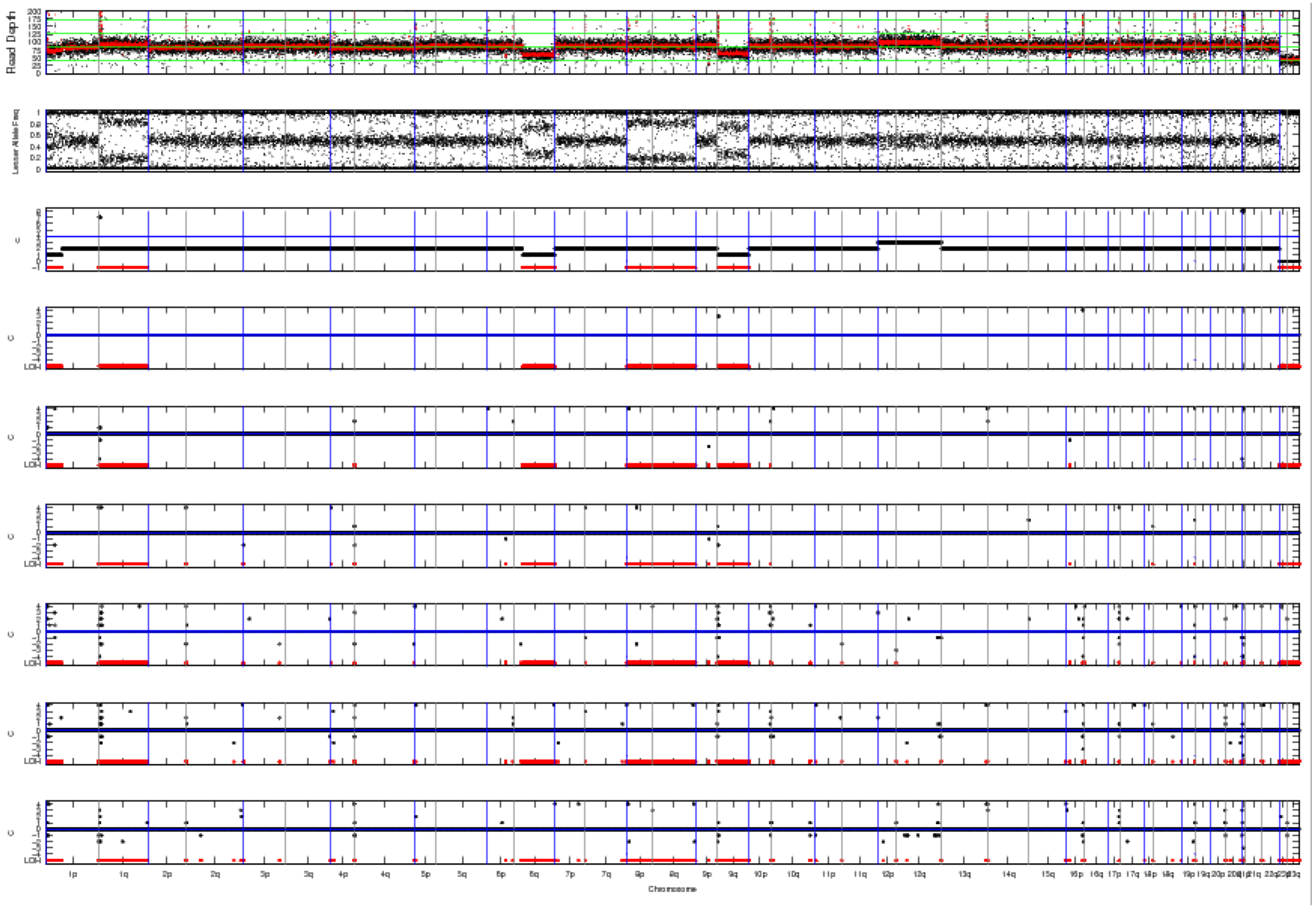

Read Depth
Lesser Allele Freq
1p
1q
2p
2q
3p
3q
4p
4q
5p
5q
6p
6q
7p
7q
8p
8q
9p
9q
10p
10q
11p
11q
12p
12q
13q
14q
15q
16p
16q
17p
17q
18p
18q
Chromosome

3034

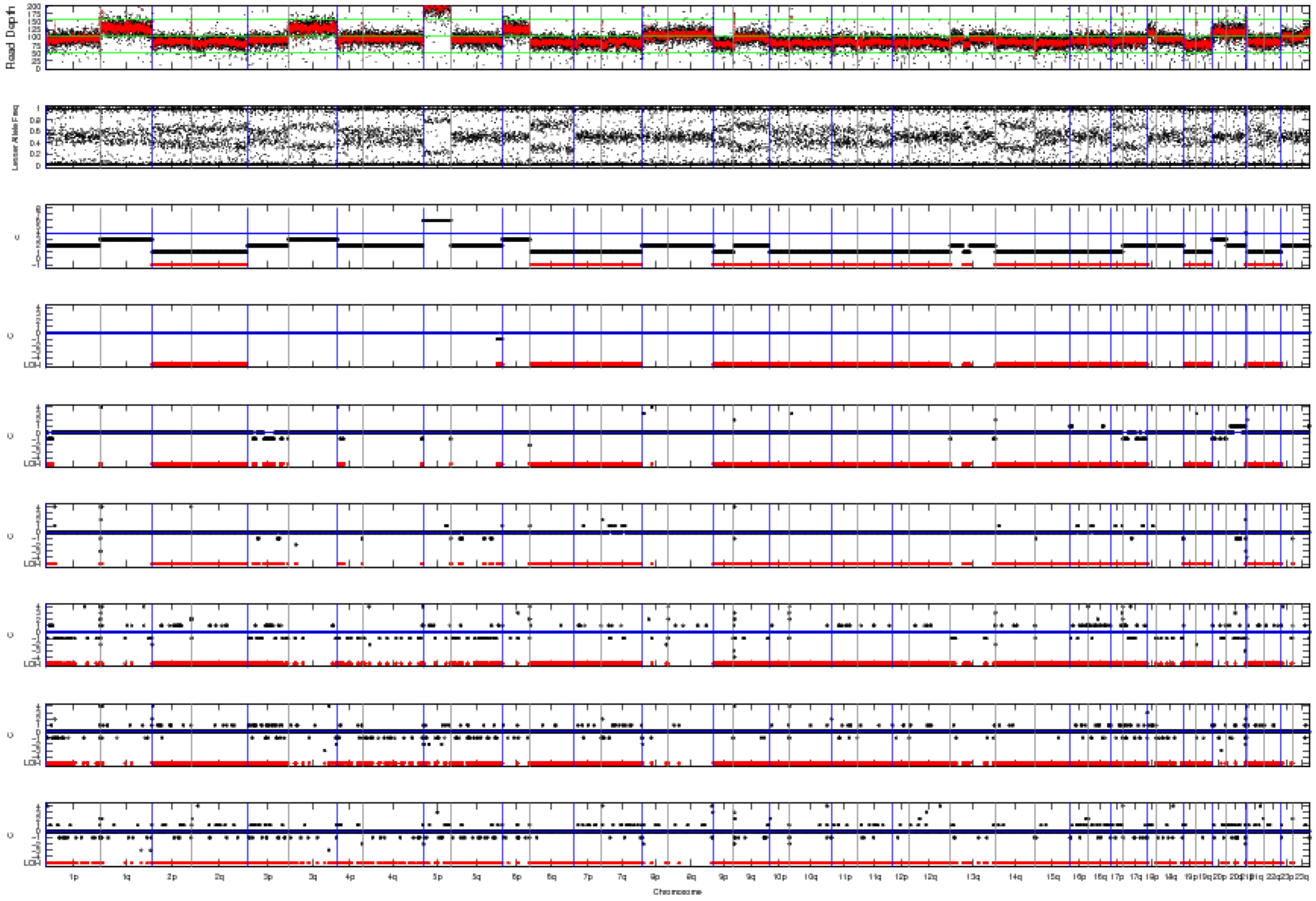

Supplement: Supplementary Data 1 — OncoSNP-Seq analysis [file ncomms4756-s2.pdf]
