# Supplementary material for: Whole-genome sequencing of bladder cancers reveals somatic CDKN1A mutations and clinicopathological associations with mutation burden
Source: Nat Commun. 2014 Apr 29;5:3756. doi: 10.1038/ncomms4756 (PMC4010643; doi:10.1038/ncomms4756)

Supplementary Data 3. Pyclone analysis

Detailed clonal frequency obtained with Pyclone is shown for the discovery set cancers. Each page corresponds to a different sample. The first figure contains the allelic frequencies of the key somatic mutations present in that sample, plus 50 random somatic mutations. The second figure contains the clustering of the similarity matrix between the somatic mutations.

See Shah, S. P. *et al*. The clonal and mutational evolution spectrum of primary triple-negative breast cancers. *Nature,* **486** 395-399 (2012)

4062

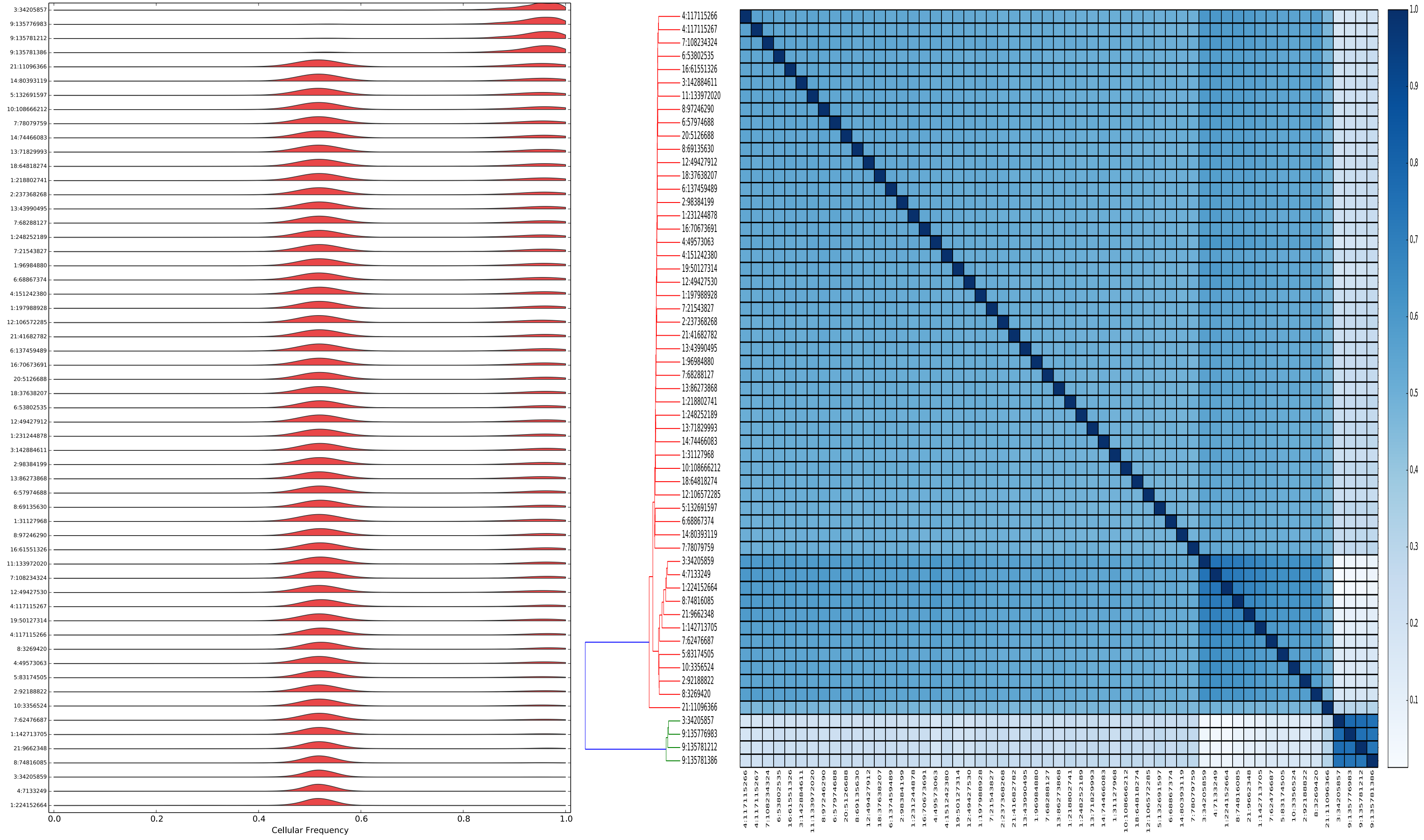

4070

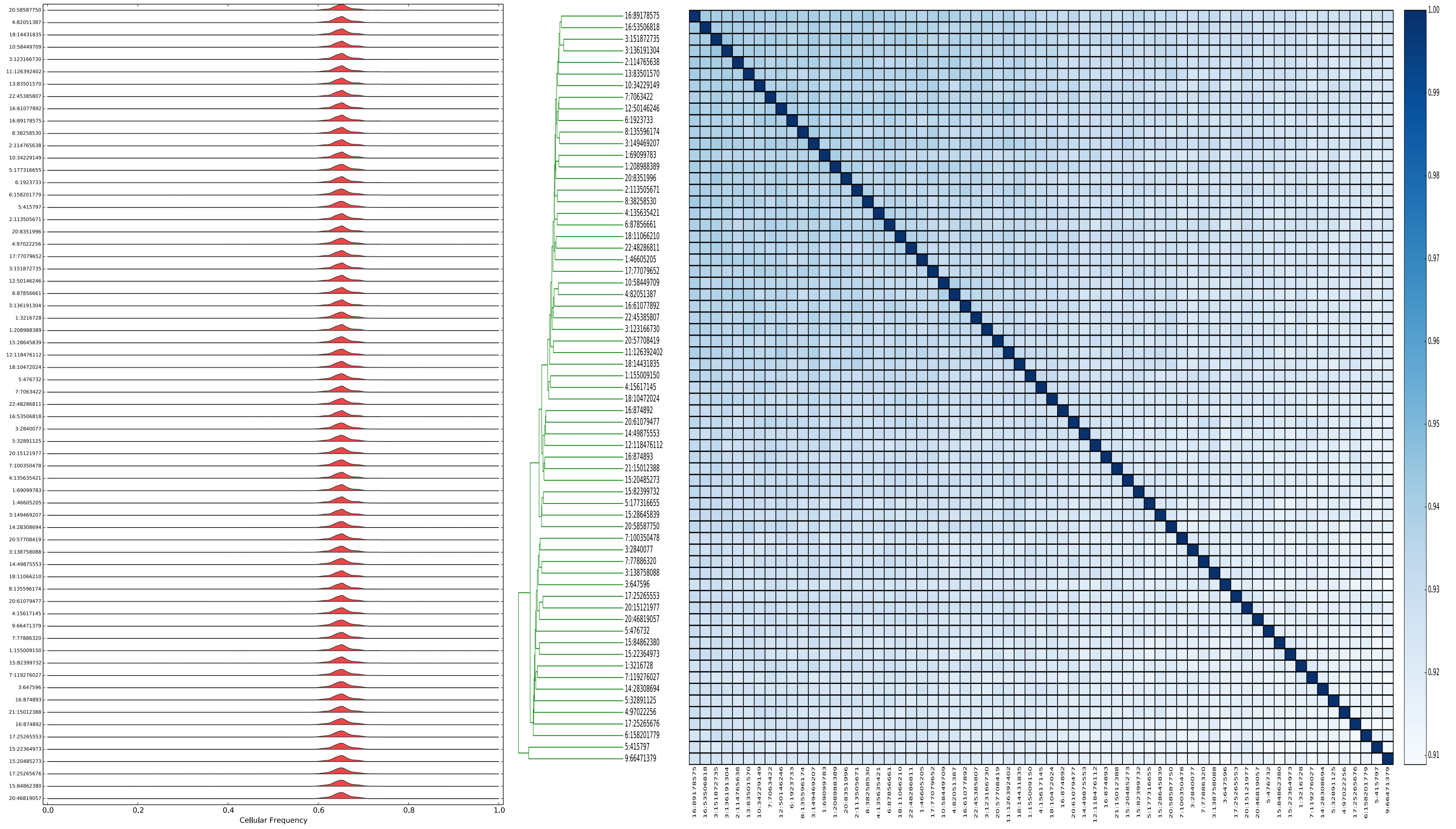

4078

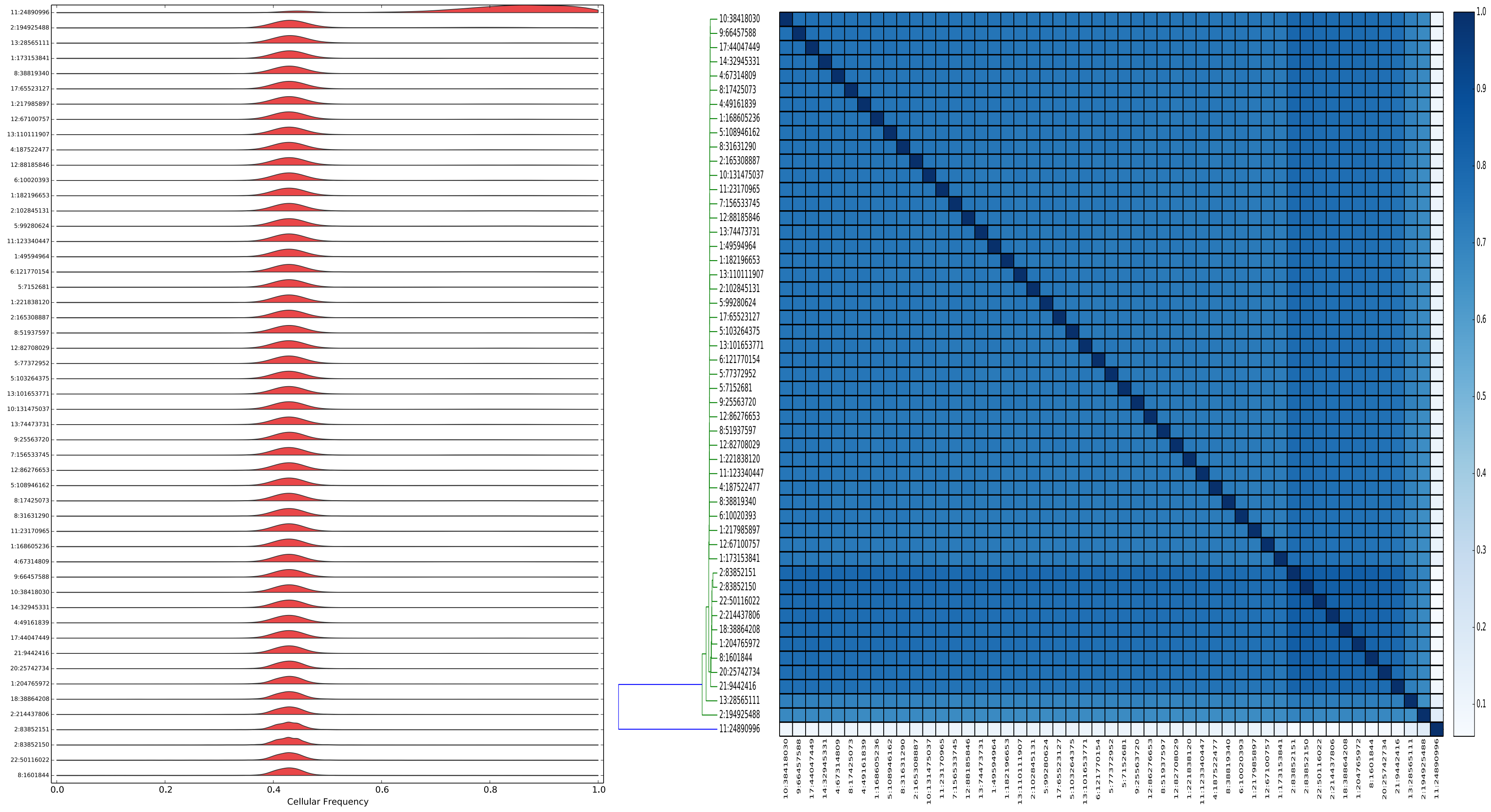

4101

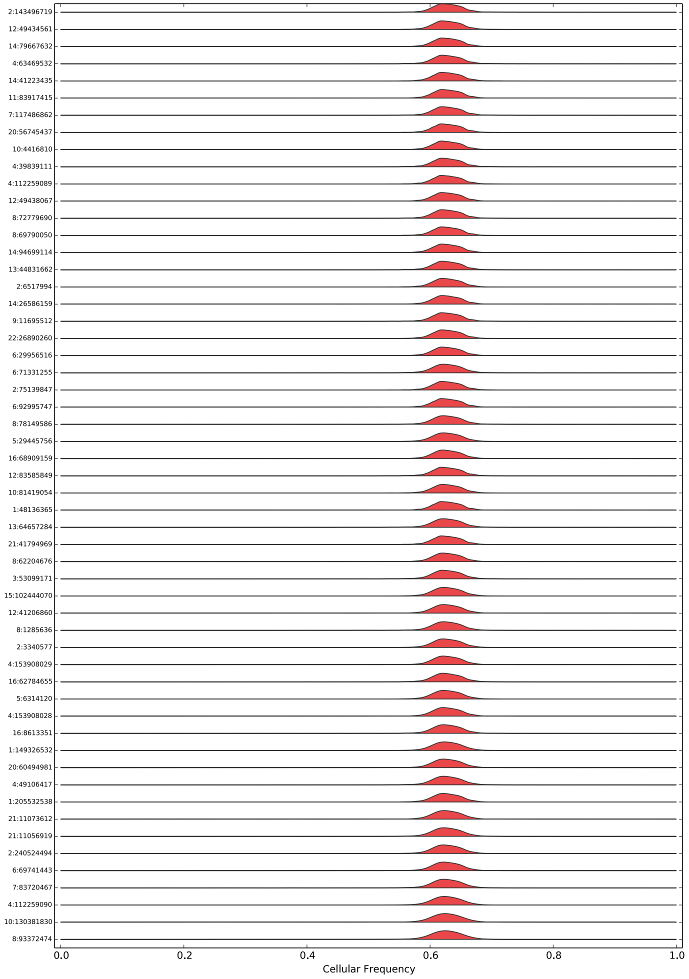

2:143496719
12:49434561
14:79667632
4:63469532
14:41223435
11:83917415
7:117486862
20:56745437
10:4416810
4:39839111
4:112259089
12:49438067
8:72779690
8:69790050
14:94699114
13:44831662
2:6517994
14:26586159
9:11695512
22:26890260
6:29956516
6:71331255
2:75139847
6:92995747
8:78149586
5:29445756
16:68909159
12:83585849
10:81419054
1:48136365
13:64657284
21:41794969
8:62204676
3:53099171
15:102444070
12:41206860
8:1285636
2:3340577
4:153908029
16:62784655
5:6314120
4:153908028
16:8613351
1:149326532
20:60494981
4:49106417
1:205532538
21:11073612
21:11056919
2:240524494
6:69741443
7:83720467
4:112259090
10:130381830
8:93372474
0.0
0.2
0.4
0.6
0.8
1.0
Cellular Frequency

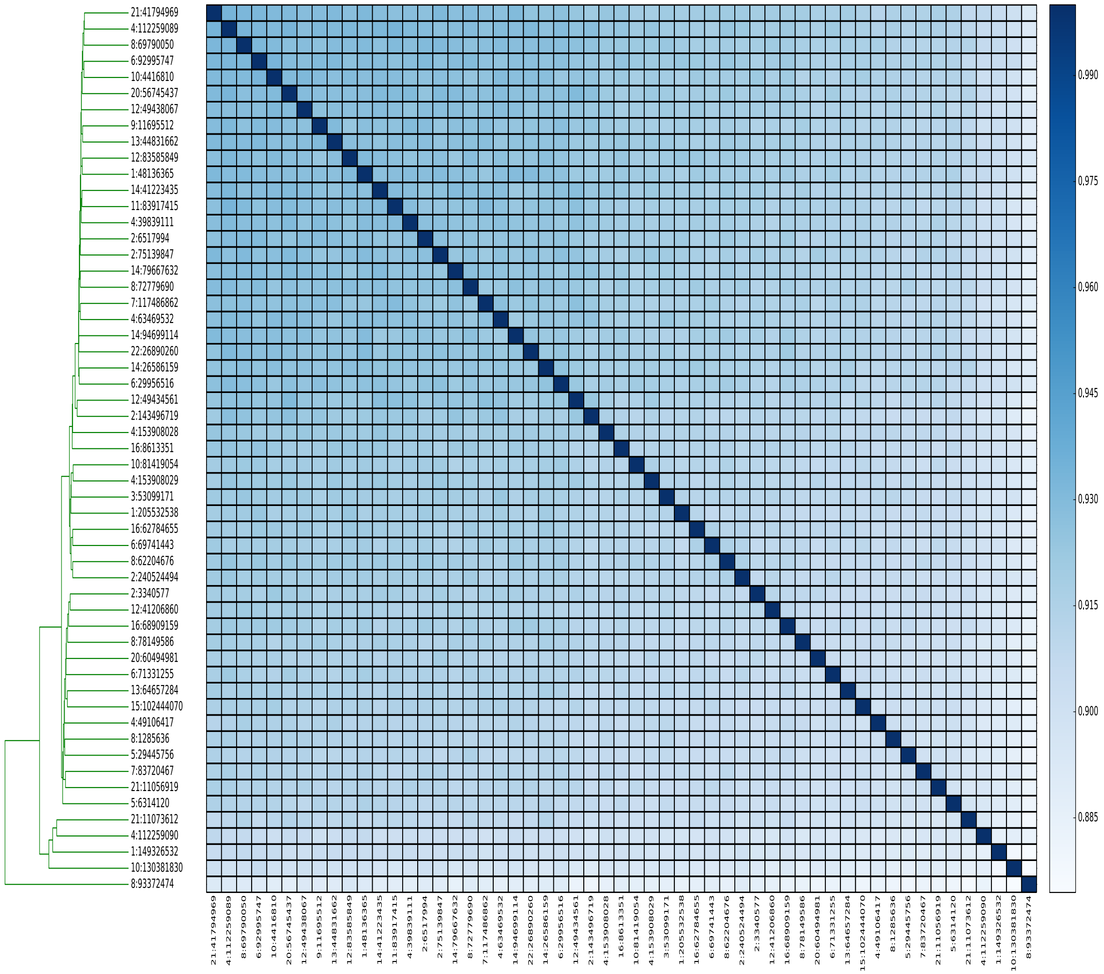

21:41794969
4:112259089
8:69790050
6:92995747
10:4416810
20:56745437
12:49438067
9:11695512
13:44831662
12:83585849
1:48136365
14:41223435
11:83917415
4:39839111
2:6517994
2:75139847
14:79667632
8:72779690
7:117486862
4:63469532
14:94699114
22:26890260
14:26586159
6:29956516
12:49434561
2:143496719
4:153908028
16:8613351
10:81419054
4:153908029
3:53099171
1:205532538
16:62784655
6:69741443
8:62204676
2:240524494
2:3340577
12:41206860
16:68909159
8:78149586
20:60494981
6:71331255
13:64657284
15:102444070
4:49106417
8:1285636
5:29445756
7:83720467
21:11056919
5:6314120
21:11073612
4:112259090
1:149326532
10:130381830
8:93372474
0.990
0.975
0.960
0.945
0.930
0.915
0.900
0.885

4121

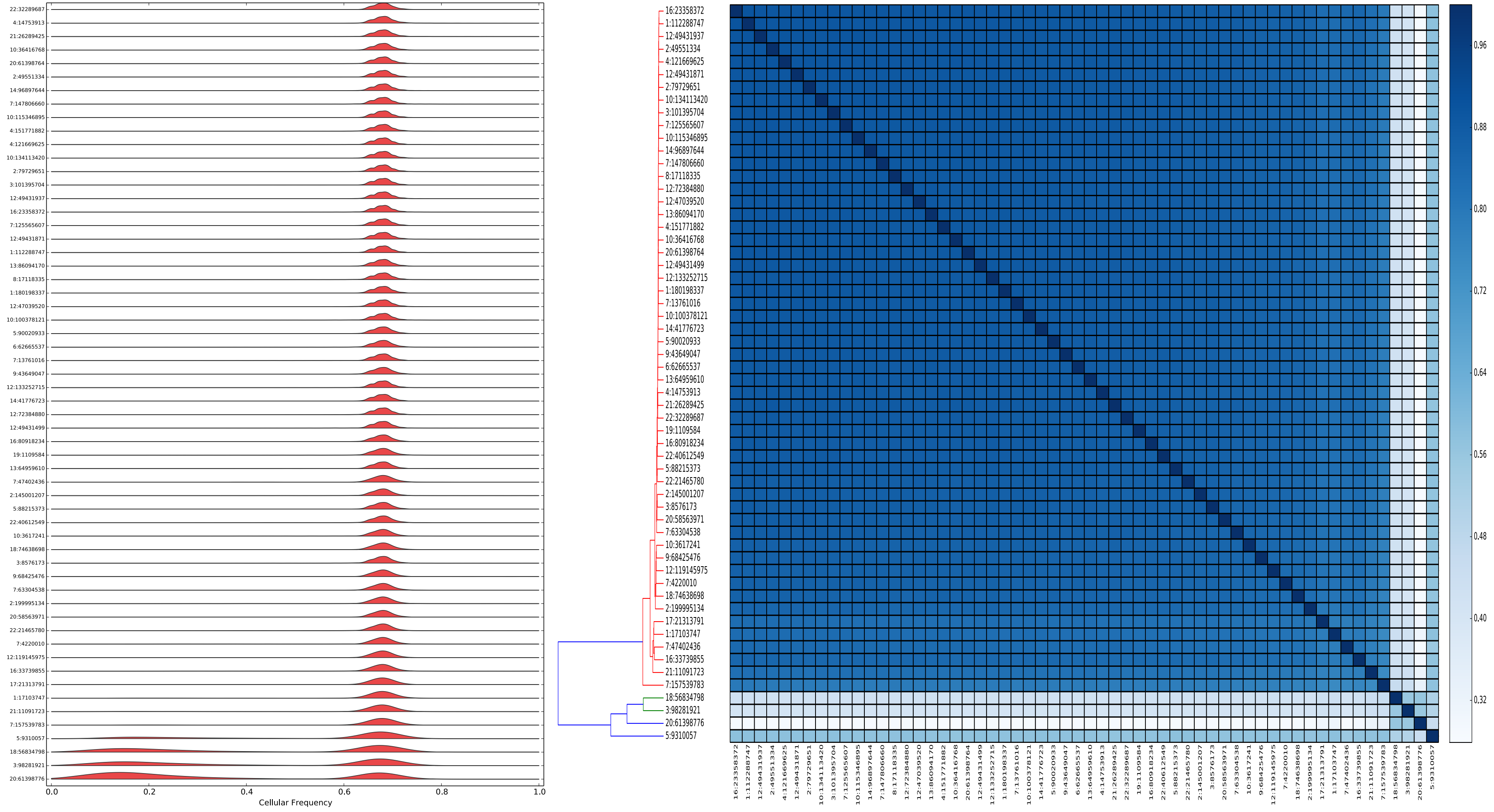

0.0
0.2
0.4
0.6
0.8
1.0
Cellular Frequency
0.96
0.88
0.80
0.72
0.64
0.56
0.48
0.40
0.32

635

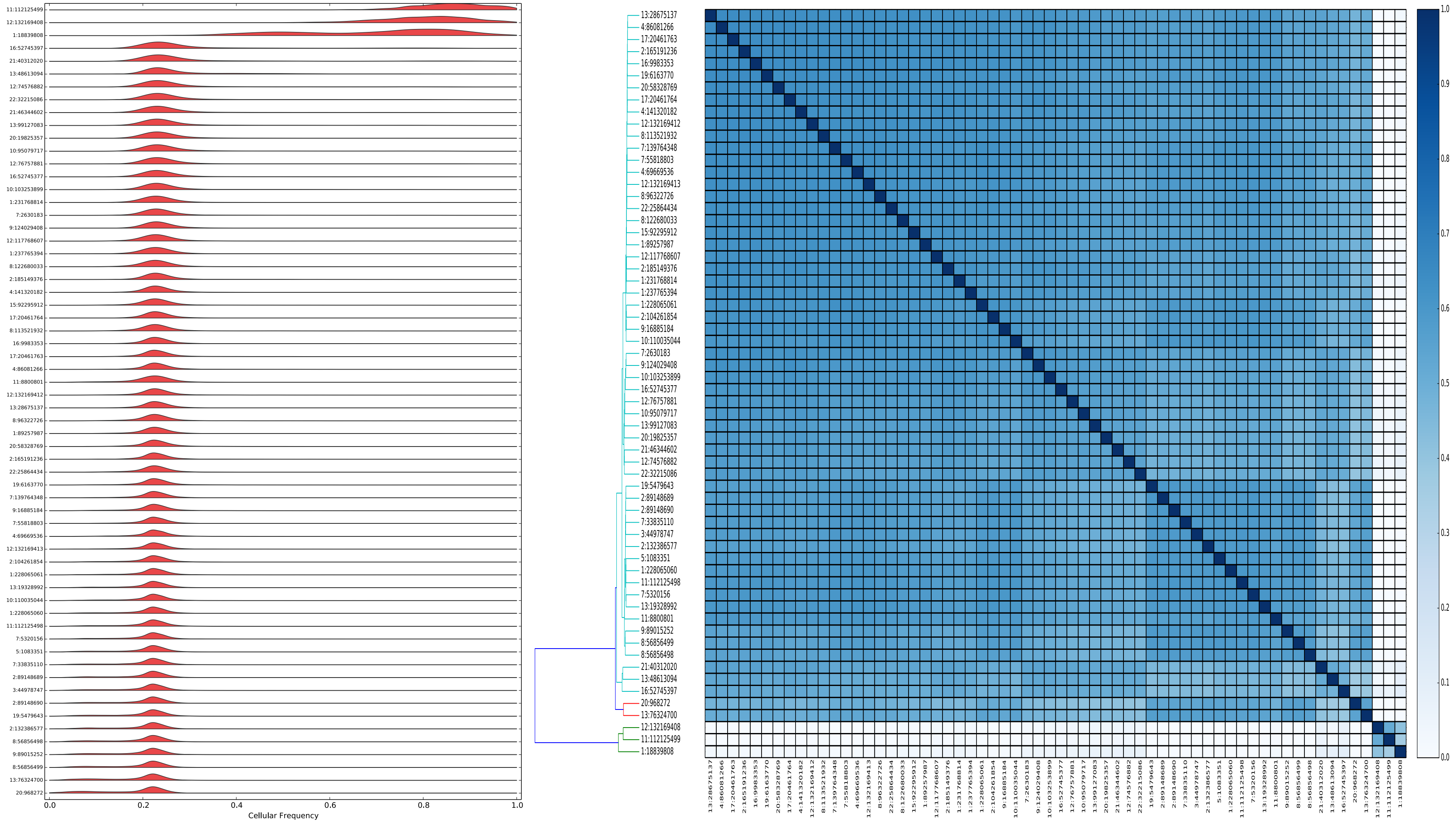

Cellular Frequency
0.0
0.2
0.4
0.6
0.8
1.0
13:28675137
4:86081266
17:20461763
2:165191236
16:9983353
19:6163770
20:58328769
17:20461764
4:141320182
12:132169412
8:113521932
7:139764348
7:55818803
4:69669536
12:132169413
8:96322726
22:25864434
8:122680033
15:92295912
1:89257987
12:117768607
2:185149376
1:231768814
1:237765394
1:228065061
2:104261854
9:16885184
10:110035044
7:2630183
9:124029408
10:103253899
16:52745377
12:76757881
10:95079717
13:99127083
20:19825357
21:46344602
12:74576882
22:32215086
19:5479643
2:89148689
2:89148690
7:33835110
3:44978747
2:132386577
5:1083351
1:228065060
11:112125498
7:5320156
13:19328992
11:8800801
9:89015252
8:56856499
8:56856498
21:40312020
13:48613094
16:52745397
20:968272
13:76324700
12:132169408
11:112125499
1:18839808
1.0
0.9
0.8
0.7
0.6
0.5
0.4
0.3
0.2
0.1
0.0

709

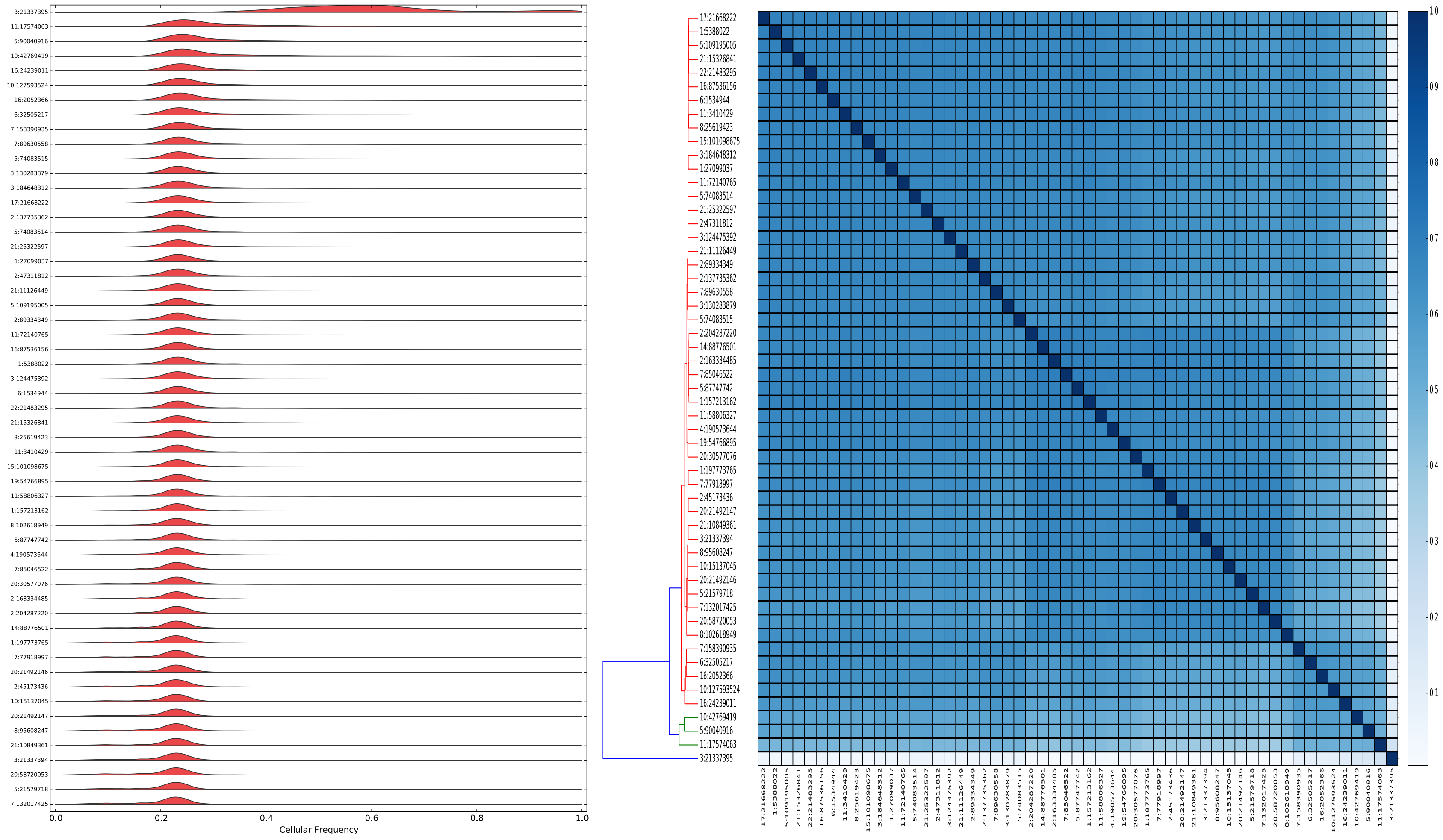
3:21337395
11:17574063
5:90040916
10:42769419
16:24239011
10:127593524
16:2052366
6:32505217
7:158390935
7:89630558
5:74083515
3:130283879
3:184648312
17:21668222
2:137735362
5:74083514
21:25322597
1:27099037
2:47311812
21:11126449
5:109195005
2:89334349
11:72140765
16:87536156
1:5388022
3:124475392
6:1534944
22:21483295
21:15326841
8:25619423
11:3410429
15:101098675
19:54766895
11:58806327
1:157213162
8:102618949
5:87747742
4:190573644
7:85046522
20:30577076
2:163334485
2:204287220
14:88776501
1:197773765
7:77918997
20:21492146
2:45173436
10:15137045
20:21492147
8:95608247
21:10849361
3:21337394
20:58720053
5:21579718
7:132017425
0.0
0.2
0.4
0.6
0.8
1.0
Cellular Frequency
17:21668222
1:5388022
5:109195005
21:15326841
22:21483295
16:87536156
6:1534944
11:3410429
8:25619423
15:101098675
3:184648312
1:27099037
11:72140765
5:74083514
21:25322597
2:47311812
3:124475392
21:11126449
2:89334349
2:137735362
7:89630558
3:130283879
5:74083515
2:204287220
14:88776501
2:163334485
7:85046522
5:87747742
1:157213162
11:58806327
4:190573644
19:54766895
20:30577076
1:197773765
7:77918997
2:45173436
20:21492147
21:10849361
3:21337394
8:95608247
10:15137045
20:21492146
5:21579718
7:132017425
20:58720053
8:102618949
7:158390935
6:32505217
16:2052366
10:127593524
16:24239011
10:42769419
5:90040916
11:17574063
3:21337395
1.0
0.9
0.8
0.7
0.6
0.5
0.4
0.3
0.2
0.1

745

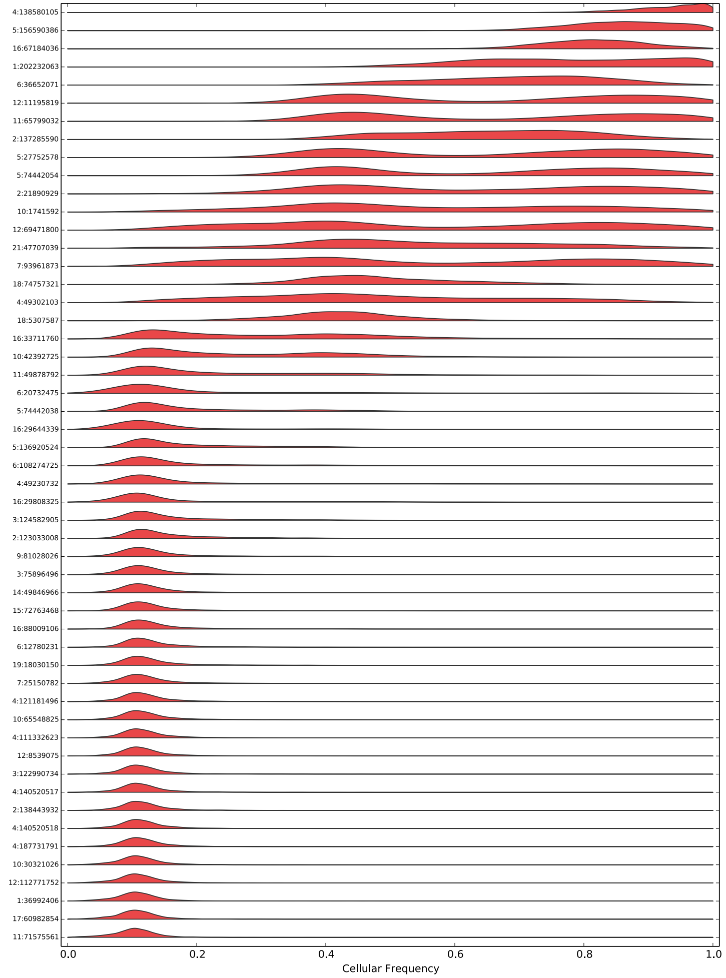
4:138580105
5:156590386
16:67184036
1:202232063
6:36652071
12:11195819
11:65799032
2:137285590
5:27752578
5:74442054
2:21890929
10:1741592
12:69471800
21:47707039
7:93961873
18:74757321
4:49302103
18:5307587
16:33711760
10:42392725
11:49878792
6:20732475
5:74442038
16:29644339
5:136920524
6:108274725
4:49230732
16:29808325
3:124582905
2:123033008
9:81028026
3:75896496
14:49846966
15:72763468
16:88009106
6:12780231
19:18030150
7:25150782
4:121181496
10:65548825
4:111332623
12:8539075
3:122990734
4:140520517
2:138443932
4:140520518
4:187731791
10:30321026
12:112771752
1:36992406
17:60982854
11:71575561
0.0
0.2
0.4
0.6
0.8
1.0
Cellular Frequency

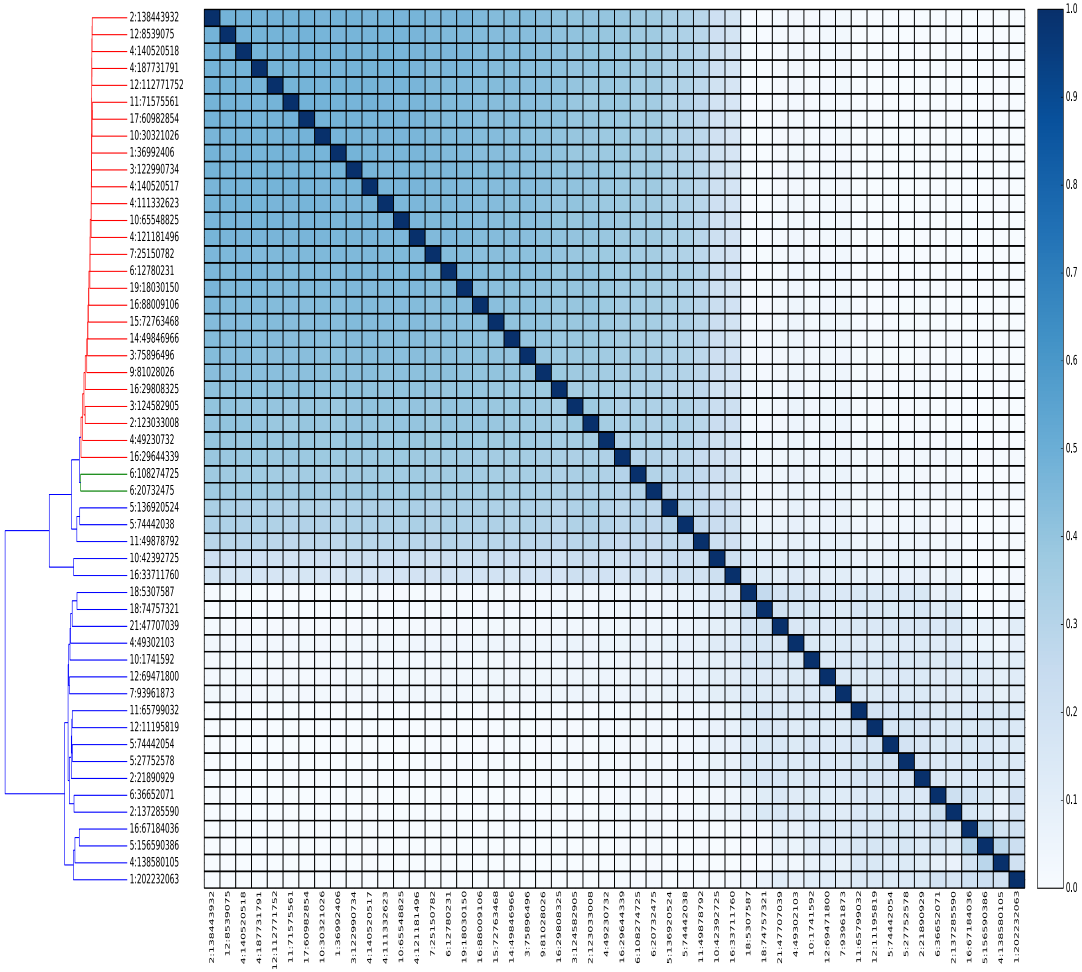
2:138443932
12:8539075
4:140520518
4:187731791
12:112771752
11:71575561
17:60982854
10:30321026
1:36992406
3:122990734
4:140520517
4:111332623
10:65548825
4:121181496
7:25150782
6:12780231
19:18030150
16:88009106
15:72763468
14:49846966
3:75896496
9:81028026
16:29808325
3:124582905
2:123033008
4:49230732
16:29644339
6:108274725
6:20732475
5:136920524
5:74442038
11:49878792
10:42392725
16:33711760
18:5307587
18:74757321
21:47707039
4:49302103
10:1741592
12:69471800
7:93961873
11:65799032
12:11195819
5:74442054
5:27752578
2:21890929
6:36652071
2:137285590
16:67184036
5:156590386
4:138580105
1:202232063
1.0
0.9
0.8
0.7
0.6
0.5
0.4
0.3
0.2
0.1
0.0

799

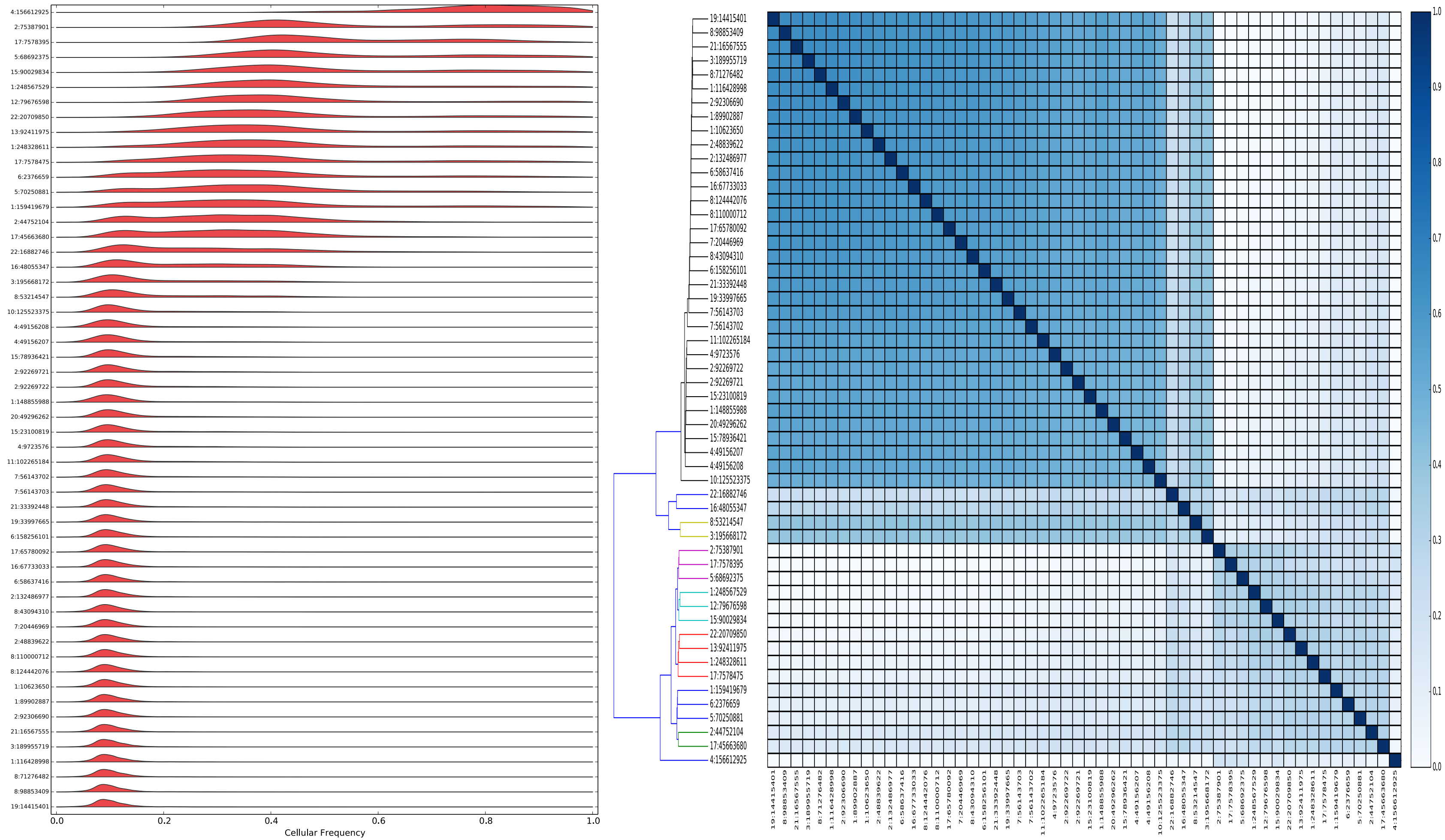

Cellular Frequency
0.0
0.2
0.4
0.6
0.8
1.0
19:14415401
8:98853409
21:16567555
3:189955719
8:71276482
1:116428998
2:92306690
1:89902887
1:10623650
2:48839622
2:132486977
6:58637416
16:67733033
8:124442076
8:110000712
17:65780092
7:20446969
8:43094310
6:158256101
21:33392448
19:33997665
7:56143703
7:56143702
11:102265184
4:9723576
2:92269722
2:92269721
15:23100819
1:148855988
20:49296262
15:78936421
4:49156207
4:49156208
10:125523375
22:16882746
16:48055347
8:53214547
3:195668172
2:75387901
17:7578395
5:68692375
1:248567529
12:79676598
15:90029834
22:20709850
13:92411975
1:248328611
17:7578475
1:159419679
6:2376659
5:70250881
2:44752104
17:45663680
4:156612925

3010

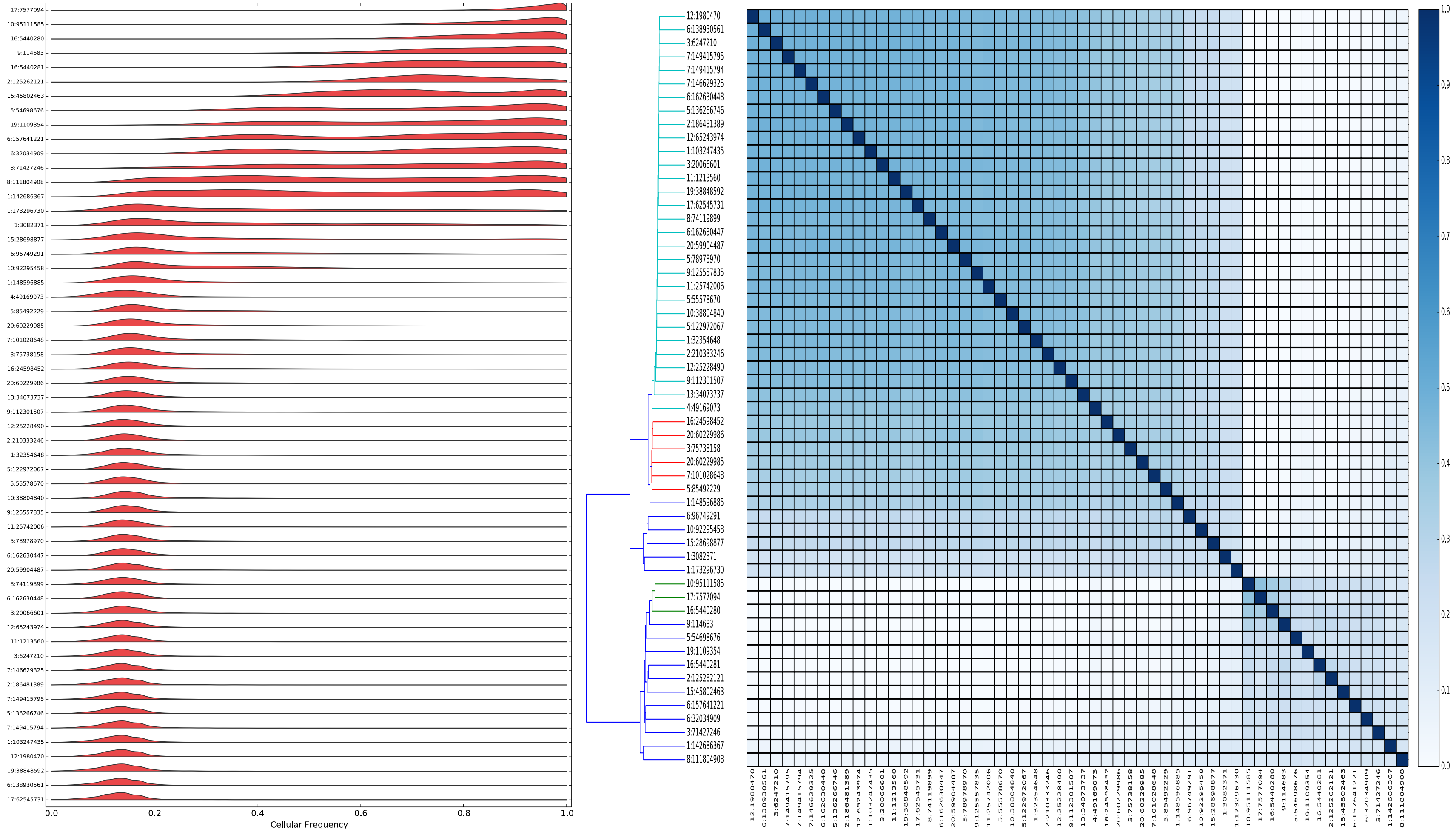

12:1980470
6:138930561
3:6247210
7:149415795
7:149415794
7:146629325
6:162630448
5:136266746
2:186481389
12:65243974
1:103247435
3:20066601
11:1213560
19:38848592
17:62545731
8:74119899
6:162630447
20:59904487
5:78978970
9:125557835
11:25742006
5:55578670
10:38804840
5:122972067
1:32354648
2:210333246
12:25228490
9:112301507
13:34073737
4:49169073
16:24598452
20:60229986
3:75738158
20:60229985
7:101028648
5:85492229
1:148596885
6:96749291
10:92295458
15:28698877
1:3082371
1:173296730
10:95111585
17:7577094
16:5440280
9:114683
5:54698676
19:1109354
16:5440281
2:125262121
15:45802463
6:157641221
6:32034909
3:71427246
1:142686367
8:111804908
0.0
0.2
0.4
0.6
0.8
1.0
Cellular Frequency

451

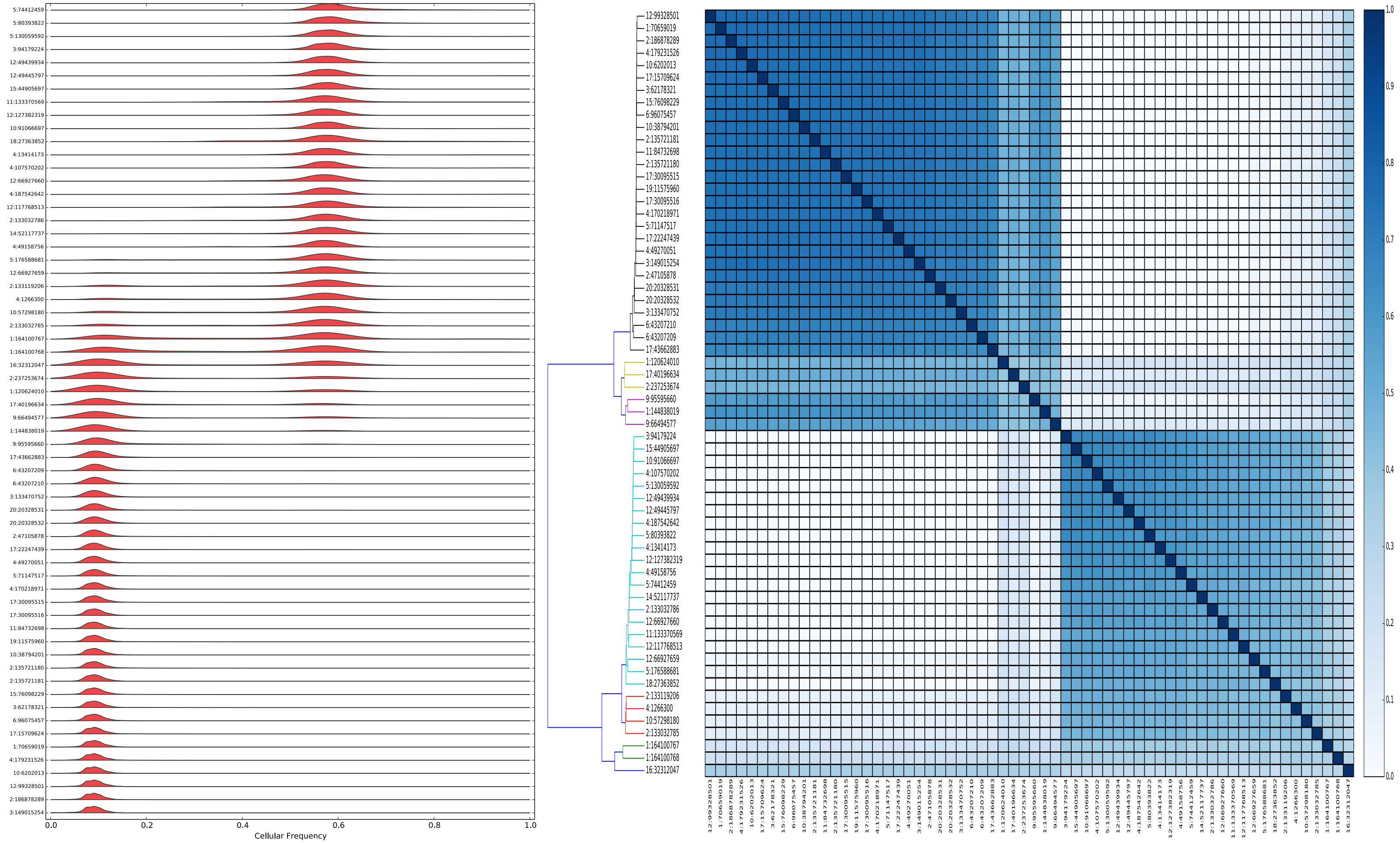
0.0
0.2
0.4
0.6
0.8
1.0
Cellular Frequency
0.0
0.1
0.2
0.3
0.4
0.5
0.6
0.7
0.8
0.9
1.0

615

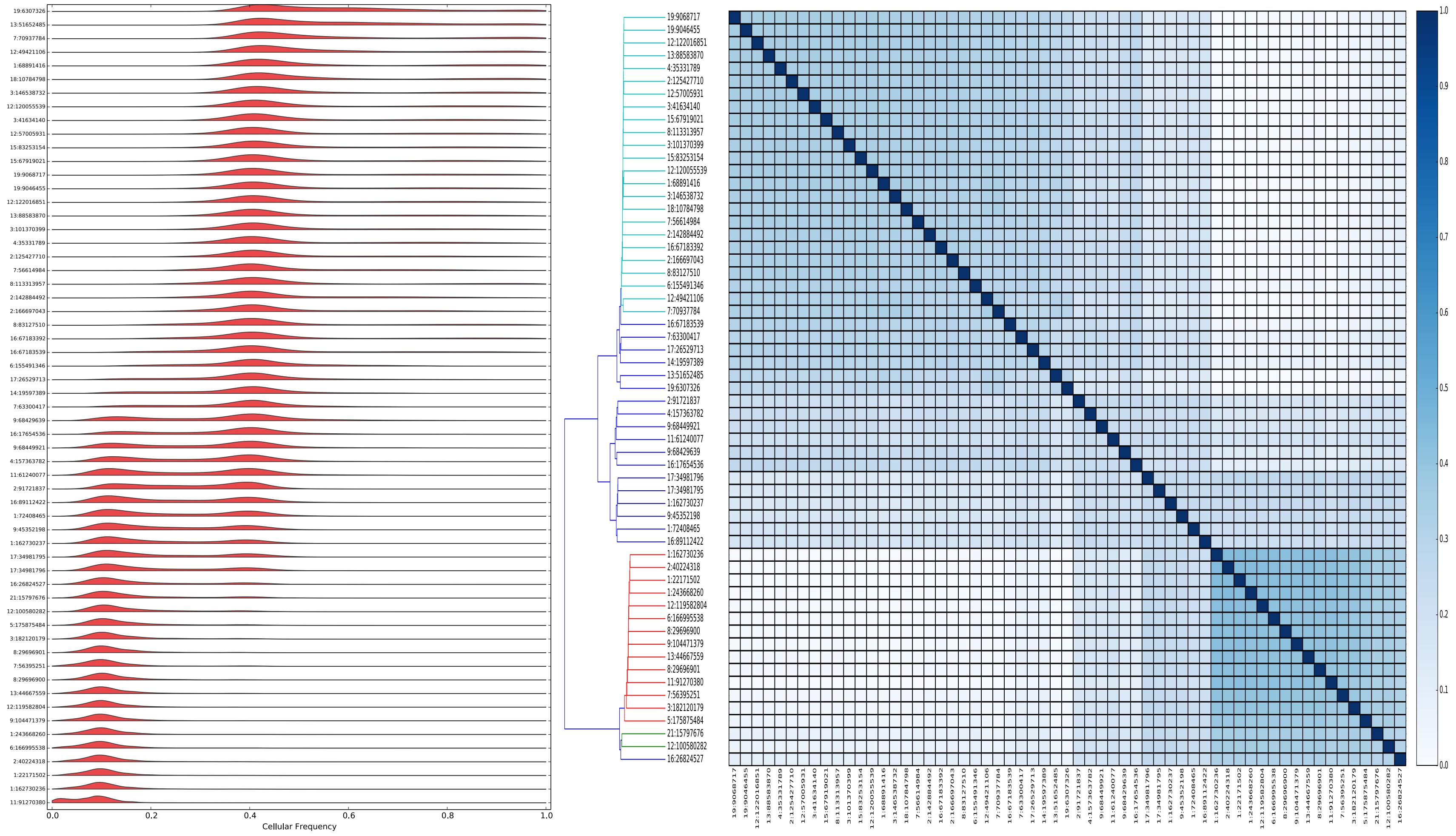

2010

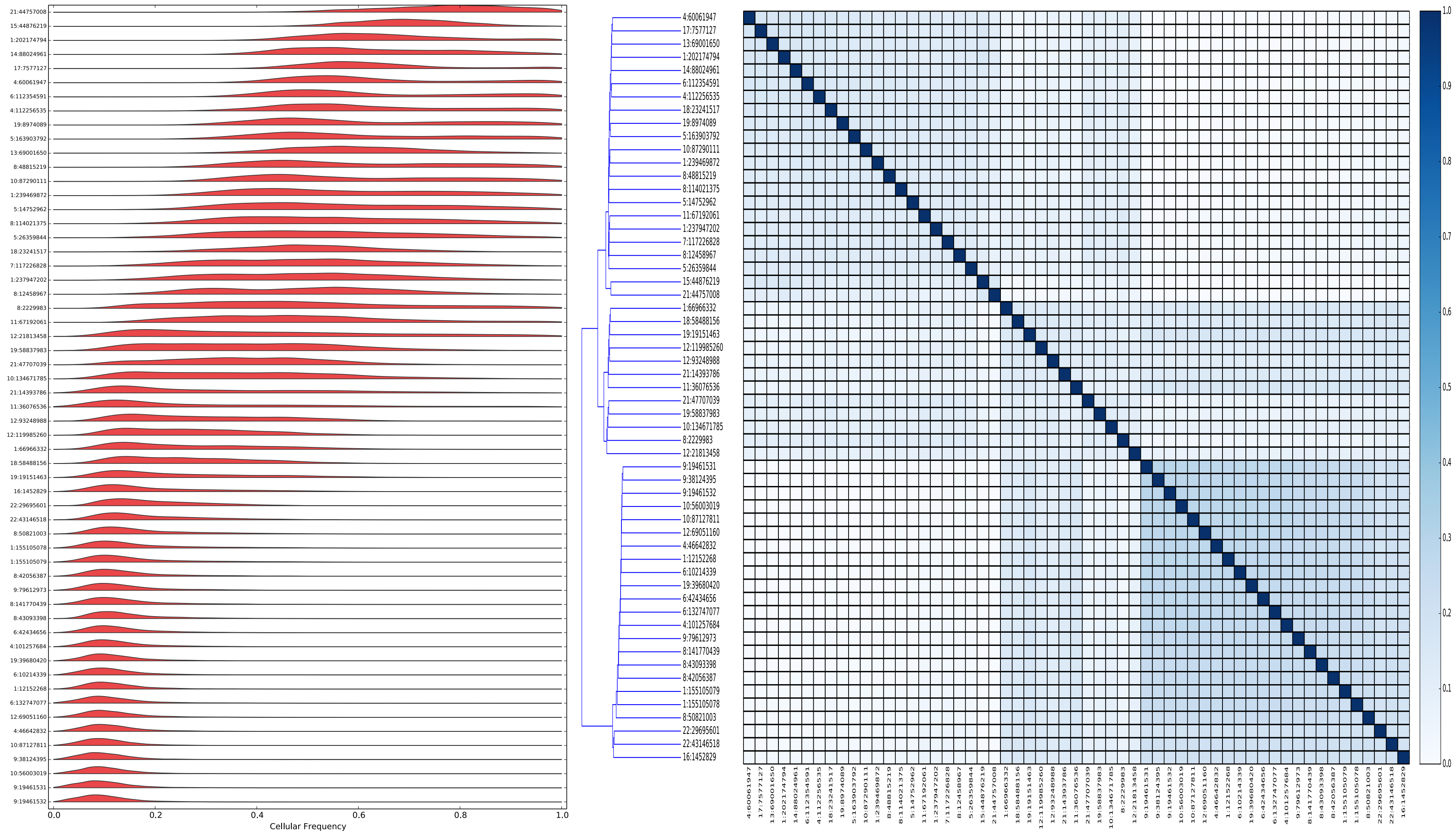

Cellular Frequency
0.0
0.2
0.4
0.6
0.8
1.0
4:60061947
17:7577127
13:69001650
1:202174794
14:88024961
6:112354591
4:112256535
18:23241517
19:8974089
5:163903792
10:87290111
1:239469872
8:48815219
8:114021375
5:14752962
11:67192061
1:237947202
7:117226828
8:12458967
5:26359844
15:44876219
21:44757008
1:66966332
18:58488156
19:19151463
12:119985260
12:93248988
21:14393786
11:36076536
21:47707039
19:58837983
10:134671785
8:2229983
12:21813458
9:19461531
9:38124395
9:19461532
10:56003019
10:87127811
12:69051160
4:46642832
1:12152268
6:10214339
19:39680420
6:42434656
6:132747077
4:101257684
9:79612973
8:141770439
8:43093398
8:42056387
1:155105079
1:155105078
8:50821003
22:29695601
22:43146518
16:1452829

3008

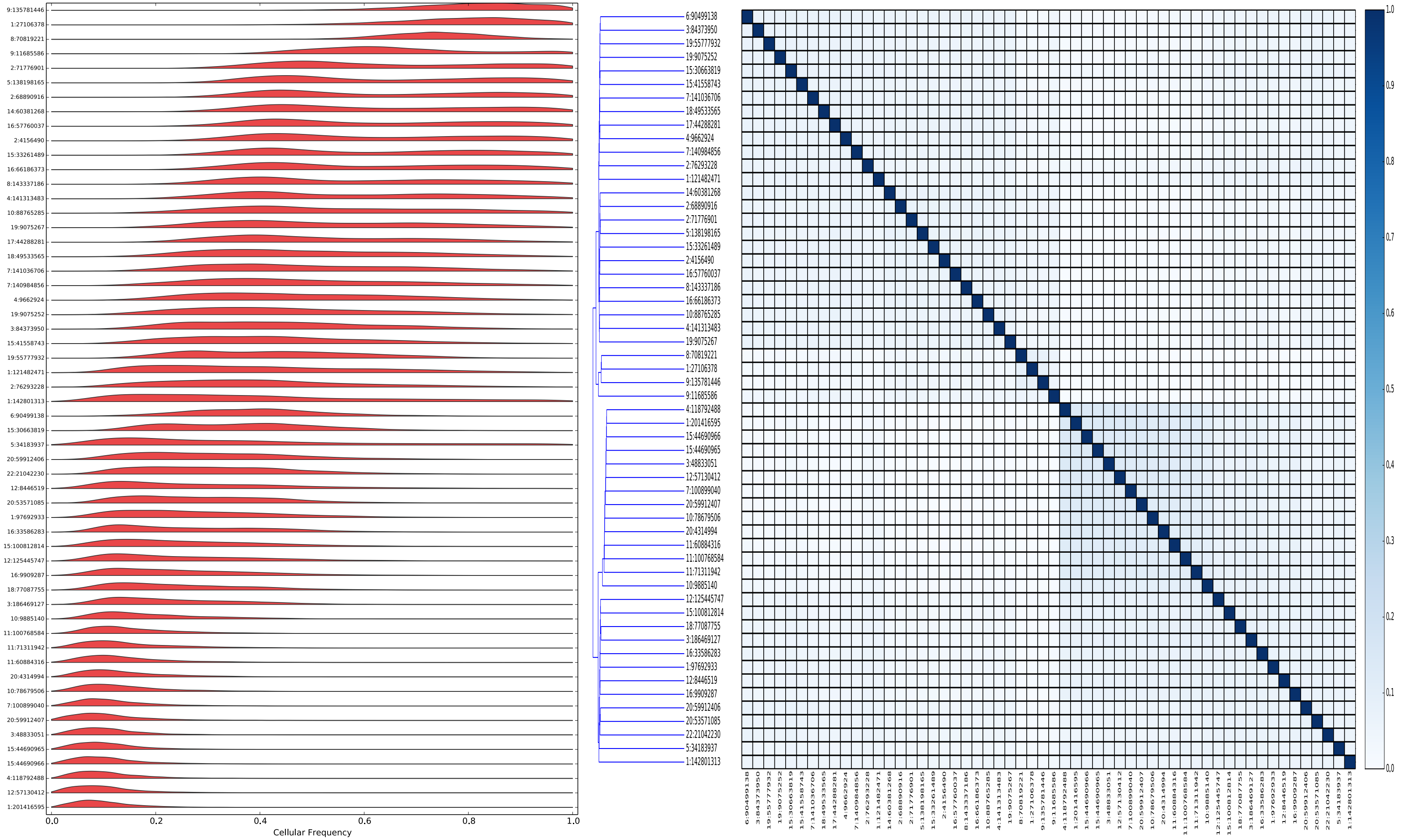

3034

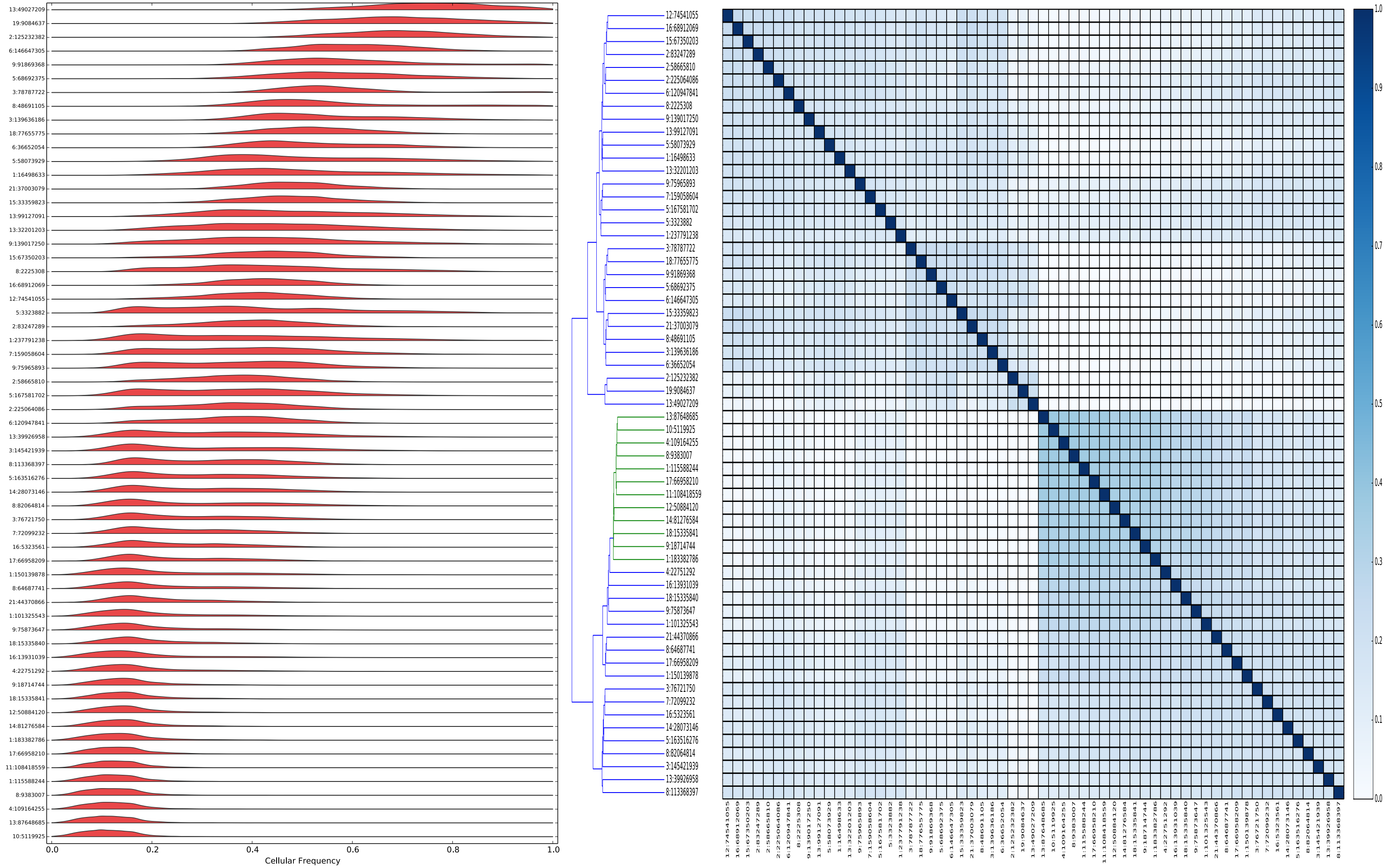

Supplement: Supplementary Data 3 — Pyclone analysis [file ncomms4756-s4.pdf]
